# Scalable and Customizable Single‐Atom Coatings for pH‐Universal H_2_O_2_ Electrosynthesis

**DOI:** 10.1002/adma.202521237

**Published:** 2026-02-27

**Authors:** Yu Li, Linguo Lu, Kunsheng Hu, Minjia Yan, Xi‐Lin Wu, Zhongfang Chen, Xiaoguang Duan

**Affiliations:** ^1^ College of Geography and Environmental Science Zhejiang Normal University Jinhua China; ^2^ Department of Chemistry University of Puerto Rico San Juan Puerto Rico USA; ^3^ School of Chemical Engineering Adelaide University Adelaide South Australia Australia

**Keywords:** catalyst coating, electrocatalysis, electrochemical H_2_O_2_ production, gas‐diffusion electrode, single‐atom catalysts

## Abstract

Achieving scalable fabrication of robust and uniform single‐atom catalyst‐based gas‐diffusion electrodes (SAC‐GDEs) remains challenging. Here, a universal one‐step soot‐deposition route was developed to convert various metal‐containing paraffins into conformal single‐atom catalyst (SAC) coatings on diverse electrodes (1D fibers, 2D plates, and 3D foams). The process provides multiscale control, from precursor‐defined molecular coordination to micropore wettability and macroscopic geometry, to collectively engineer hierarchical coating films that couple intensified mass transfer and high intrinsic catalytic activity for efficient H_2_O_2_ electrosynthesis. As a device‐level demonstration, Pd‐SAC‐GDE delivers pH‐universal H_2_O_2_ production under an industrial‐level current (500 mA cm^−2^) for 100 h, achieving a record‐high H_2_O_2_ yield of 16.9 mol g^−1^ h^−1^. A tip‐enhanced mechanism was proposed based on constant‐potential calculations. The results reveal that the curvature‐enhanced localized electric field promotes O_2_ polarization and activation at the Pd‐O_3_ sites, thereby facilitating both ^*^OOH generation and adsorption and ultimately leading to highly selective H_2_O_2_ production. This facile, broadly applicable fabrication strategy significantly advances the scalable manufacture of SAC‐coated GDEs for environmental and sustainable catalysis.

## Graphical abstract

A universal one‐step soot‐deposition route was developed to convert various metal‐containing paraffins into conformal single‐atom catalyst (SAC) coatings on diverse electrodes, yielding robust, efficient SAC‐coated gas diffusion electrodes for H_2_O_2_ electrosynthesis.

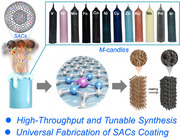

## 1 Introduction

Single‐atom catalysts (SACs) are featured by atomically dispersed metal sites with well‐defined local coordination environments, which maximizes metal atom efficiency and often leads to extraordinary reactivity and selectivity [1, 2, 3, 4]. Their performances are dictated not only by the local coordination environment and medium‐to‐strong metal‐support electronic interactions, but also by the density of accessible active sites [5, 6, 7]. Increasing metal loadings and enlarging the accessible surface area of the support are typical approaches for accelerating reaction kinetics [8, 9]. However, increasing the loading typically promotes metal‐atom migration and aggregation, thereby increasing metal consumption and ultimately compromising both performance and cost.

Various synthetic strategies, including wet impregnation, atomic layer deposition, galvanic replacement, chemical vapor deposition, and high‐temperature pyrolysis [10, 11], have driven significant advances in SAC fabrication. However, these methods often require high‐temperature pyrolysis [12], high vacuum [13], inert‐gas protection [14], and specialized equipment [13, 15, 16], which limit scalability and complicate continuous and low‐cost manufacturing. Moreover, most SAC materials are prepared as fine powders that require post‐processing for catalytic applications, such as loading onto a substrate scaffold, packing into columns, or depositing a slurry/paste onto electrodes in practical implementation [17, 18]. Such steps often result in densely packed layers that impair both electron and mass transport and risk structural collapse or detachment under demanding operational conditions, posing nontrivial barriers to both electrode and system scale‐up [19].

These challenges are especially critical for gas‐involving electrocatalytic reactions (e.g., water splitting, O_2_ reduction, CO_2_ reduction, and N_2_ reduction) [19, 20], where most conventional gas diffusion electrodes (GDEs) are prepared with catalyst pastes rather than in situ fabrication [21, 22], leading to poor interfacial kinetics and unsatisfactory long‐term stability under industrially relevant conditions [22, 23, 24]. Progress toward robust and high‐performance SAC‐coated GDEs is constrained by (i) preserving the catalytically active structure and structural integrity of SAC coatings on electrodes, and (ii) tailoring key surface properties, such as hydrophobicity and porosity of GDEs, that collectively control gas and electrolyte transport in multiphase electrochemical reactions. Consequently, there is a pressing need for facile, scalable strategies to directly fabricate robust, uniform, and tunable SAC coatings on technologically relevant electrodes.

Here, we present a soot‐deposition strategy that couples the one‐step synthesis of an SAC library (incorporating up to 12 metal species) with universal, direct coating of diverse electrode substrates. This mild, rapid, and scalable route enables precise, multiscale control over SAC coatings at the atomic (coordination and composition), microscopic (porosity and surface wettability), and macroscopic (coating thickness and morphology) levels. The resulting SAC‐coated GDEs exhibit excellent catalytic activity and selectivity in electrochemical H_2_O_2_ production and U(VI) recycling, while maintaining exceptional mechanical integrity and operational durability, thereby advancing state‐of‐the‐art SACs toward practical electrocatalysis for environmental applications.

## 2 Results and Discussion

### 2.1 Tunable Synthesis and Characterization

**FIGURE 1:**
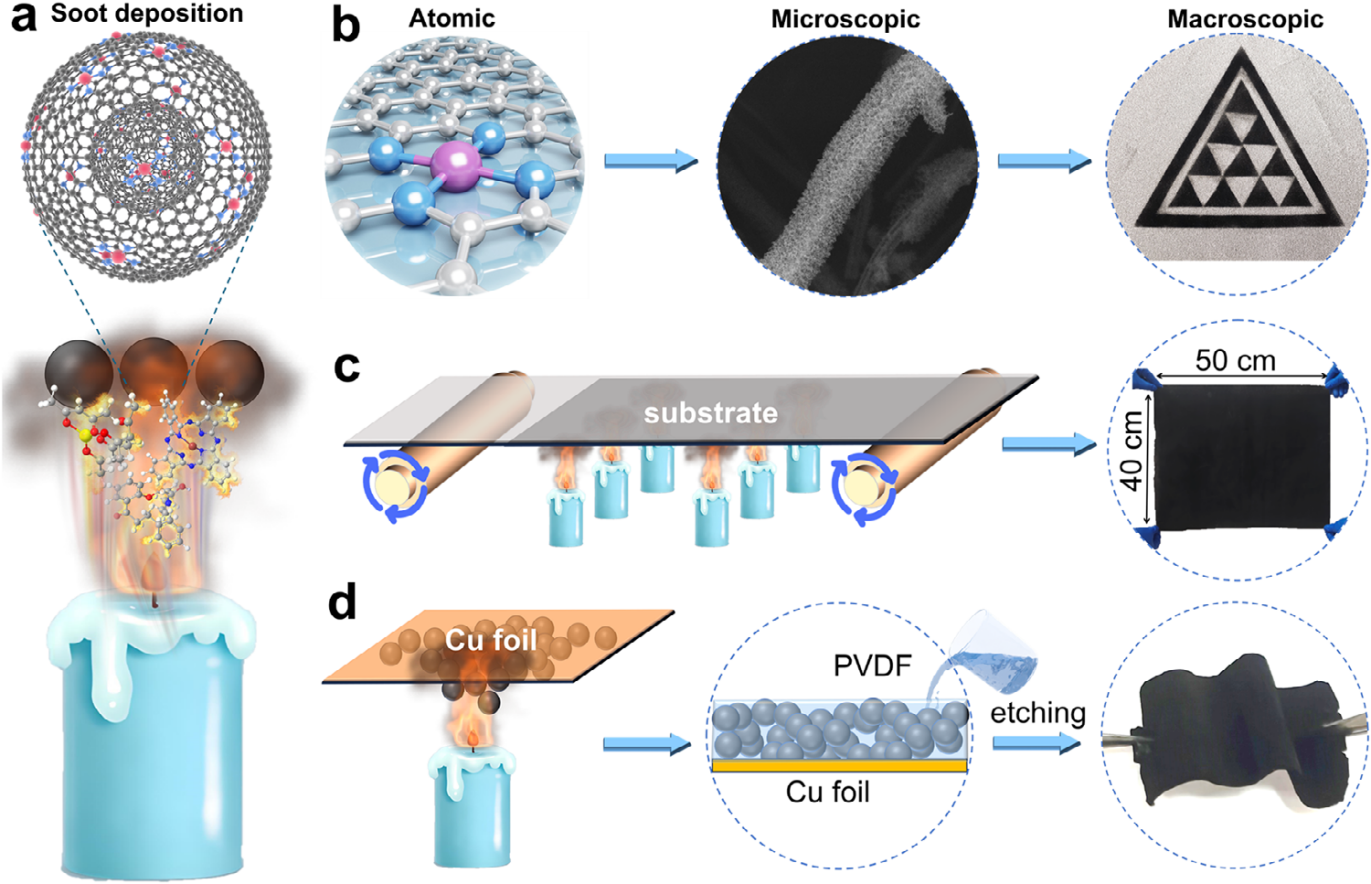
Schematic illustrating the tunable fabrication of SAC‐coating. (a) One‐step soot deposition synthesis of SACs. (b) Multiscale regulation of SAC coating: atomic (metal identity/coordination), microscopic (porosity/wettability), and macroscopic (thickness, patterning). (c, d) Scalable fabrication on large‐area electrodes (c) and flexible membranes (d).

Achieving high‐performance SAC‐based GDEs requires not only intrinsically active single sites but also a versatile fabrication route with multiscale tunability. To this end, we introduce a one‐step soot‐deposition strategy in which a metal precursor co‐melted with paraffin wax is molded into an M‐candle (Figure S1). Upon ignition, the M‐candle generates single‐atom metal (SA‐M) sites confined within candle soot (CS) and directly deposits them onto electrode substrates uniformly with atomic‐to‐macroscopic precision (Figure 1a,b).

During the combustion process, the high‐temperature candle flame melts paraffin wax and metal precursors, subsequently subliming and decomposing them into ions, free radicals, polyacetylenes, and polycyclic aromatic hydrocarbons [25]. These crucial intermediates ultimately participated in the nucleation and growth of CS, while the metal species catalyzed soot formation [26]. Notably, the flame combustion approach enables the rapid synthesis of well‐defined SA‐M‐CS nanoparticles, along with straightforward control over the coordination environment and reactivity of single‐atom metal sites. As examples, cobalt acetylacetonate, cobalt phthalocyanine (CoPc), and vitamin B12 precursors generate Co–O_4_, Co–N_4_, and Co–N_5_ sites, respectively, each associated with distinct electrocatalytic activities (Figure S2).

At the microscale, the SAC coating forms a hierarchical porous network on the electrode surface, which facilitates gas–liquid transport during three‐phase reactions, and maximizes the exposure of accessible single‐atom catalytic sites. At the device level, the coating area, shape, and compliance can be readily tailored to fit the target substrate (Figure 1; Figure S3). Notably, this strategy facilitates SACs depositing onto large‐area electrode substrates (Figure 1c) and flexible membranes (Figure 1d), both of which function effectively as GDEs for gas‐involving electrocatalysis [22, 27]. Moreover, the wettability of the SAC coating is tunable via the thickness of soot deposition, as confirmed by water contact angle measurements (Figure S3). Collectively, this multiscale control, from coordination, porosity, and geometry to surface wettability, provides a straightforward approach to customize and manufacture SAC‐based GDEs for gas‐involving electrocatalysis [28].

**FIGURE 2:**
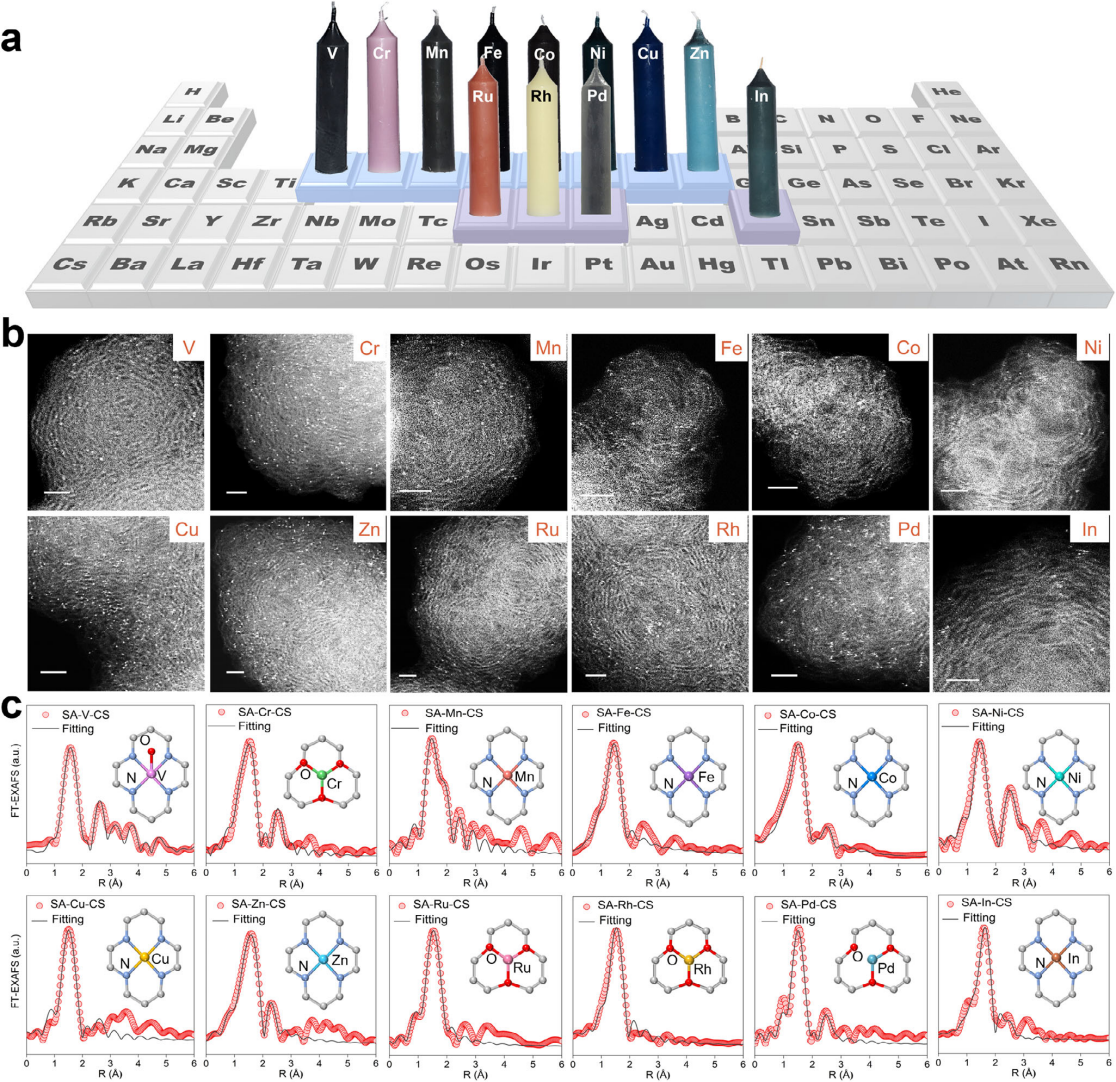
The M‐candles and characterizations of the SACs library. (a) Schematic of the metal‐paraffin “M‐candle” used for one‐step soot deposition of SA–M–CS coatings. (b) HAADF‐STEM images of SA–M–CS SACs showing atomically dispersed metal sites on graphitic carbon nanospheres: scale bar, 2 nm. (c) FT‐EXAFS spectra (gray) with corresponding fitting (red) of SA–M–CS SACs, confirming single‐atom coordination environments; insets depict the dominant coordination motifs.

This one‐step soot‐deposition strategy affords a versatile SAC library encompassing twelve metals (M = V, Cr, Mn, Fe, Co, Ni, Cu, Zn, Ru, Rh, Pd, In) (Figure 2a). The metal loading is readily tunable: increasing precursor content in the candle enhances metal density, whereas excessive precursor leads to cluster or nanoparticle formation, as confirmed by X‐ray diffraction (XRD) and high‐angle annular dark‐field (HAADF) scanning transmission electron microscopy (STEM) (Figure S4). To prevent the single‐atom metals from aggregating, the metal loadings of SA‐M‐CS SACs were controlled at approximately 0.5 wt.%–0.6 wt.% (Table S1). Moreover, the coated carbon matrix is highly conductive and exhibits a porous structure arising from the stacking of carbon spheres. XRD and Raman confirm a high degree of graphitization (Figure S4), which lowers charge‐transfer resistance. The N_2_ adsorption‐desorption measurements indicate that the Brunauer‐Emmett‐Teller (BET) surface areas and pore sizes of the SA‐M‐CS SACs range from 50 to 70 m^2^ g^−1^ and 6–12 nm, respectively (Figure S4 and Table S2). This suggests a mesopore‐dominated structure, which is conducive to the transport of multiphase gases and electrolytes. The slight variations in BET surface areas and pore sizes may be attributed to differences in the combustion and catalytic properties of the various metal precursors, which could influence the nucleation and growth of the candle soot.

Correlative HAADF–STEM images (Figure 2b; Figures S5–) revealed that the single‐atom metal sites are embedded throughout the onion‐like graphitic carbon layers of SA–M–CS nanospheres with an average size of ∼40 nm, with no discernible nanoparticles or clusters. Quantitative XAFS spectra and fitting results (Figure 2c; Table S3) demonstrated that the atomic configuration of the SA–M–CS catalysts correlates with the original coordination of their corresponding parent metal precursors: M–phthalocyanines yield predominantly M–N_4_ sites, whereas M–acetylacetonates favor M–O_4(3)_ coordination. These observations confirm that the soot‐deposition approach preserves atomic dispersion while transferring precursor‐defined coordination structures to the support, enabling direct control of the coordination chemistry of active sites alongside the macroscopic porous coating architecture. Thus, this approach provides a versatile platform for the advanced design and regulation of SAC coatings from the molecule‐level catalytic center to the macroscopic electrode interfacial structures.

### 2.2 Universal Fabrication of SAC Coatings

**FIGURE 3:**
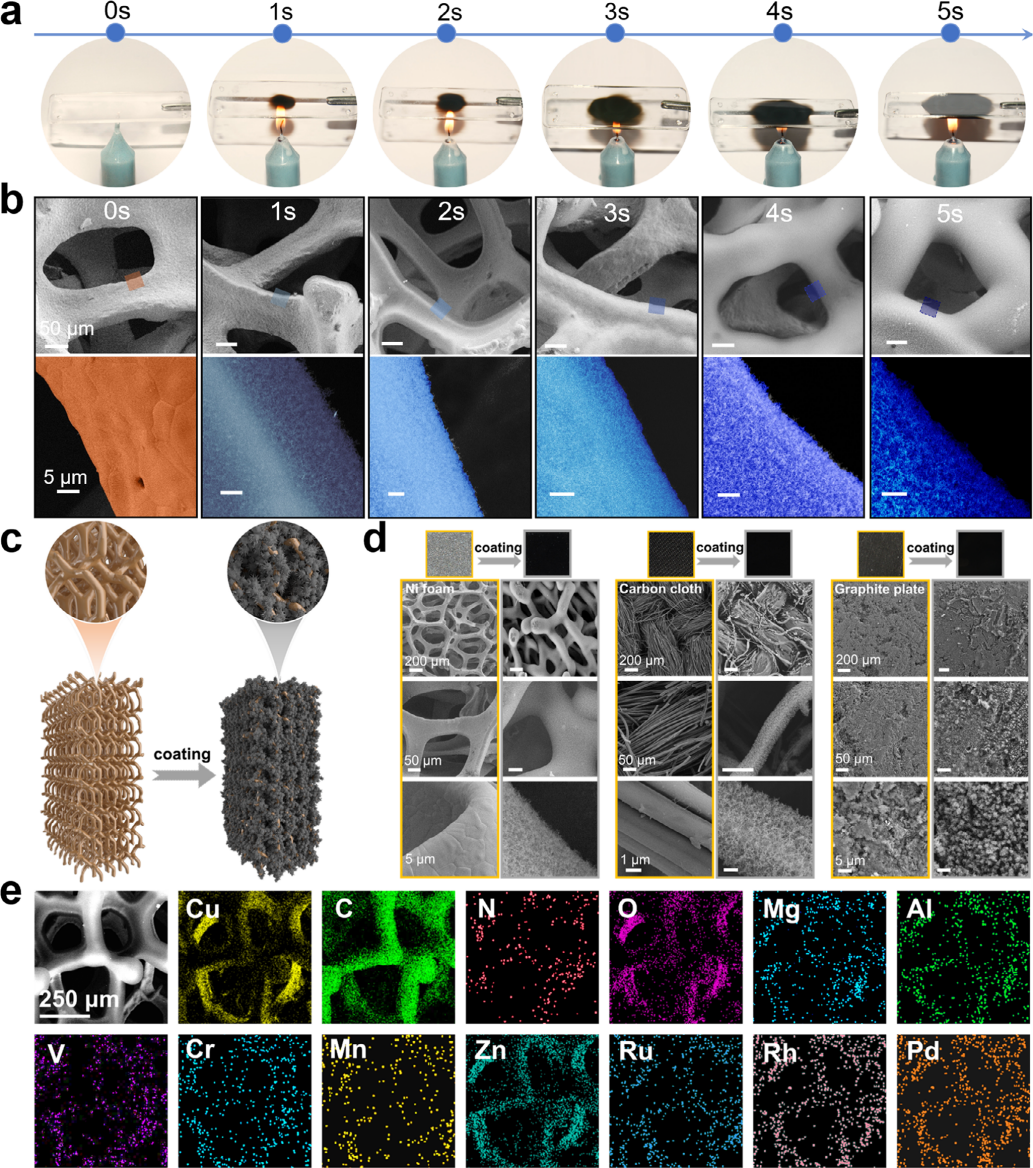
Universal fabrication of SACs coating. (a) Digital images showing rapid film formation by soot deposition. (b) Time‐dependent SEM images on Cu foam reveal progressive scaffold infilling and growth of a porous coating with deposition time. (c) Schematic of the conformal SAC coating on a porous electrode, forming a hierarchical mesoporous–macrporous architecture. (d) Conformal coatings on diverse substrates: Ni foam, carbon cloth, and graphite plate. (e) SEM‐EDS elemental mapping of multi‐metal coating on Cu foam, demonstrating uniform dispersion of nine single‐atom metals.

Furthermore, this soot‐deposition strategy can be applied across diverse substrates to deposit well‐defined SAC coatings. On glass, a uniform black coating layer forms within seconds, affording rapid SAC soot deposition (Figure 3a). On Cu foam, time‐dependent SEM (Figure 3b) revealed progressive scaffold infilling: the initially rough surface became increasingly conformal as soot accumulated, while close‐up views confirmed a fluffy, porous, and low‐density layer, with thickness increasing with extended deposition duration. The higher‐resolution SEM images confirm the presence of fluffy carbon nanoparticles with an interconnected porous structure, forming a uniform coating on the substrate surface. Unlike conventional slurry coating methods, which typically form a dense, nonuniform catalyst layer, this protocol enables the in situ deposition of a uniform, porous, and highly reactive SAC layer on diverse substrates. Importantly, the mesoporous SA–M–CS conforms to the macroscopic foam network (Figure 3c), forming a hierarchical macro‐mesoporous architecture that provides continuous diffusion pathways for gas and ion transport [29]. These advantages facilitate three‐phase mass transfer throughout the macroporous electrode framework and reactions at the mesoporous SAC interface [30].

To demonstrate the broad applicability of this soot‐deposition strategy for universal electrode fabrication, the SA–M–CS coatings were loaded on representative substrates ranging from 1D microfibers to 3D foams. Following the coating processes, the surfaces of nickel foam (silvery), copper foam (reddish‐brown), and carbon paper (gray) all became uniformly smooth and black (Figure 3d; Figures S17 and S18). To assess the uniformity of the SAC coating on the large‐area electrode, scanning electron microscopy (SEM) images of representative regions were analyzed (Figure S19). The results indicate that the coating thickness in the sampled areas is consistent, demonstrating uniform coverage across the entire electrode surface. Likewise, carbon foam, carbon cloth, indium tin oxide (ITO) glass, and graphite plates can be coated with a fluffy and porous SA–M–CS layer, as confirmed by SEM analysis. Furthermore, this approach is compositionally versatile, enabling the spontaneous deposition of multi‐metal (up to nine species) SAC coatings, as confirmed by the energy‐dispersive X‐ray spectroscopy (EDS) mapping (Figure 3e; Figure S20). Consequently, this coating strategy facilitates rapid, tunable, and scalable fabrication of SAC‐coated electrodes, providing a cost‐effective approach for developing versatile and programmable GDEs for electrocatalysis.

### 2.3 Performances of SAC Coatings

SAC‐coated GDE exhibits low interfacial resistance and large electrochemically accessible areas. Electrochemical impedance spectroscopy (EIS) showed that 12 SAC‐coated Cu foams with different metals possess significantly lower resistance than Cu foams coated with commercial powder catalysts, such as Pt/C, Pd/C, and RuO_2_ (Figure S21); this is due to the great integrity of the in situ deposited SAC coating that minimizes interfacial charge‐transfer resistance. The electrochemical surface areas (ECSA) of the SA–M–CS catalysts are measured via cyclic voltammetry (CV) (Figure S22). The resulting ECSA values are in the range of 2–42 mF cm^−2^ (Figure S23), comparable to or even greater than those of state‐of‐the‐art SACs (Table S4), demonstrating the outstanding accessibility to the intrinsic catalytic atomic sites.

The coatings also deliver superior activity across various electrochemical reactions. As cathodic catalysts, SA–Ni–CS, SA–Cu–CS, SA–Ru–CS, and SA–Rh–CS match or surpass the commercial 20% Pt/C for hydrogen evolution reaction (HER), while SA–Ni–CS and SA–Rh–CS outperform benchmark RuO_2_ in oxygen evolution reaction (OER). Especially, among the tested SACs, SA–Pd–CS with a Pd‐O_3_ coordination exhibits the highest ring current (0.57 mA), recorded by linear sweep voltammetry (LSV) and 98% selectivity for the two‐electron (2e^−^) oxygen reduction reaction (ORR) (Figure S24), establishing it as the most promising SACs for electrochemical H_2_O_2_ production.

**FIGURE 4:**
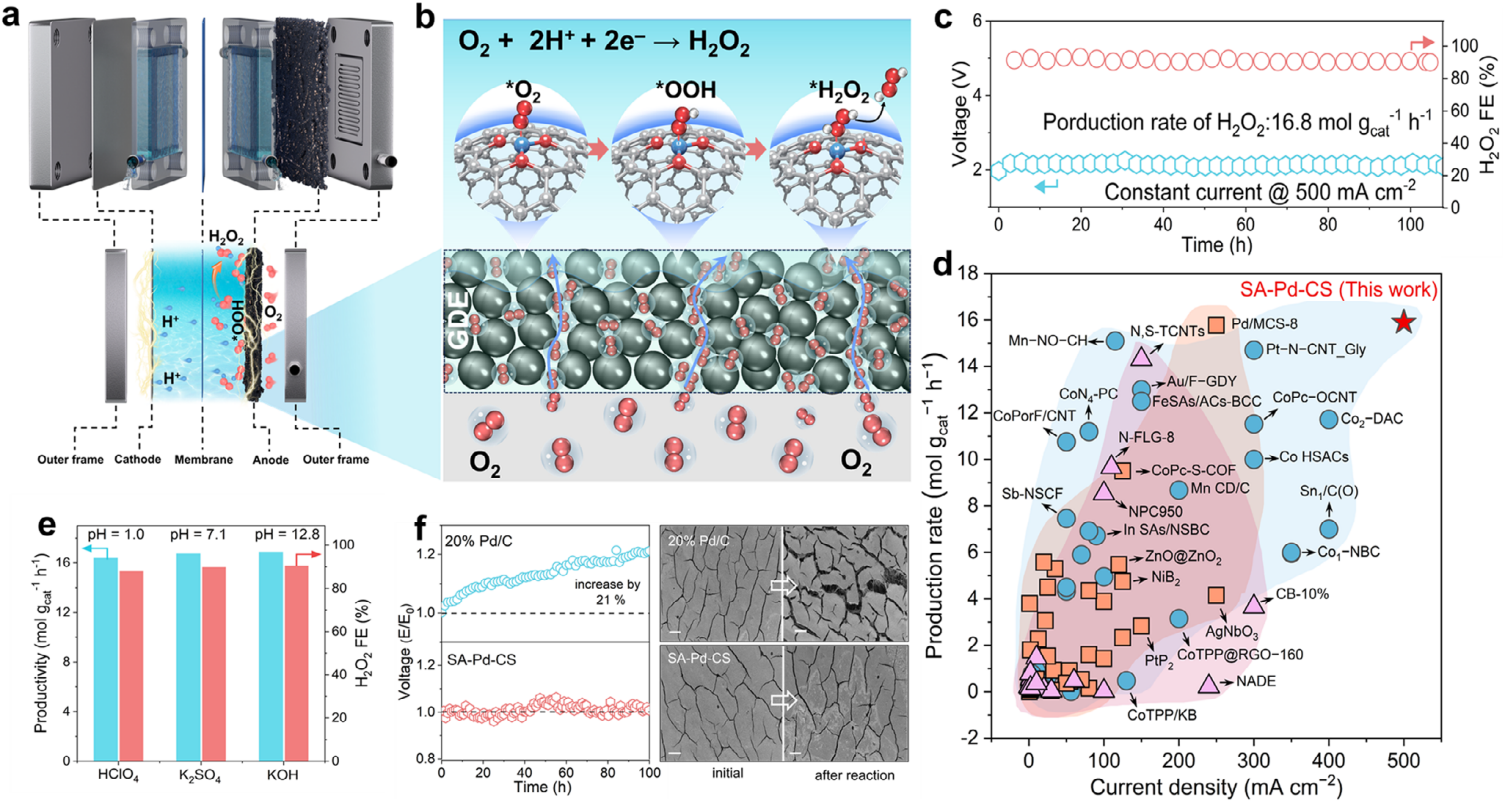
Device‐level performance of SA‐Pd‐CS‐coated GDE for electrochemical H_2_O_2_ production. (a) Schematic of the microfluidic flow cell reactor in an O_2_‐saturated electrolyte. (b) Illustration of hierarchical mass transport that accelerates *OOH formation and H_2_O_2_ production. (c) Long‐term performance of the SA‐Pd‐CS‐coated GDE for H_2_O_2_ production at 500 mA cm^−2^. (d) Benchmarking against reported catalyst‐coated electrodes for H_2_O_2_ electrosynthesis. (e) H_2_O_2_ production rate and Faraday efficiency (FE_H2O2_) in acidic (0.1 m HClO_4_), neutral (0.5 m K_2_SO_4_), and alkaline (0.1 m KOH) electrolytes. (f) Stability comparison of SA‐Pd‐CS‐GDE and commercial Pd/C‐coated GDE at 500 mA cm^−2^ in 1 m KOH, and the corresponding SEM images before and after long‐term operation. Scale bar, 300 µm.

To evaluate the practical performance of SA–Pd–CS–coated GDE, a microfluidic flow cell was constructed using a SA–Pd–CS–coated carbon paper cathode in O_2_‐saturated electrolyte, and operated at an industrially relevant current density of 500 mA cm^−2^ (Figure 4a). The hierarchically porous architecture of the engineered electrode provides abundant diffusion channels, thus accelerating three‐phase transport of both gases and liquids to the accessible single‐atom sites (Figure 4b). The enhanced mass transfer enabled rapid O_2_ accumulation and adsorption, promoting subsequent ^*^OOH and H_2_O_2_ production via O_2_ reduction.

Furthermore, we quantify the mass‐transfer advantage via limiting‐current analysis (Figure S25). Compared to the powder‐coated SA–Pd–CS electrode, the in situ soot‐deposited SA–Pd–CS electrode exhibited higher current densities across various O_2_ flow rates, underscoring accelerated reaction kinetics due to improved mass transfer [31, 32]. The negligible current response for H_2_O_2_ reduction under N_2_ implies prohibited H_2_O_2_ decomposition over the cathode, giving rise to the high 2e^−^ ORR selectivity (Figure S26). Collectively, these results demonstrate that the hierarchical mesoporous–microporous architecture of the hydrophobic GDE enhances O_2_ transport and replenishment at the SAC interface for selective 2e^−^ ORR, meanwhile facilitating H_2_O_2_ diffusion into the solvent without consumption by the cathode. This overcomes the key limitation of typical transition metal‐based 2e^−^ ORR systems, where SAC sites (e.g., Co, Fe, Mn) would further consume the generated H_2_O_2_ via direct decomposition (into O_2_), Fenton‐like reaction (into ^•^OH), or cathode reduction, prohibiting the accumulation of H_2_O_2_ [32].

The SA–Pd–CS‐GDE maintains excellent H_2_O_2_ production performance over 100 h at 500 mA cm^−2^ (Figure 4c). Furthermore, when benchmarked against reported state‐of‐the‐art SACs and nanocatalysts (Figure 4d; Tables S5–S8), SA–Pd–CS‐GDE achieves the highest production rates at the elevated current density. Notably, SA–Pd–CS‐GDE adapts to various pH conditions, achieving record‐high H_2_O_2_ productivities of 16.4, 16.8, and 16.9 mol g_cat_^−1^ h^−1^ at industry‐relevant current density (500 mA cm^−2^) with high Faradaic efficiencies of 87.9%, 89.8% and 90.8% in acidic (pH 1.0), neutral (pH 7.1), and alkaline (pH 12.8) electrolytes (Figure 4e), respectively. Unlike state‐of‐the‐art catalysts, which suffer from poor pH tolerance and low Faradaic efficiency under high current densities (Table S4), the SA–Pd–CS–GDE exhibits broad pH applicability and high Faradaic efficiency, attributable to suppressed side reactions, excellent atomic coordination stability, and a hydrophobic mesoporous microenvironment. These results collectively underscore the excellent robustness and broad pH applicability of the SAC coatings for electrochemical H_2_O_2_ production.

To assess the durability under industrially relevant conditions, SA–Pd–CS was benchmarked against a commercial 20 wt.% Pd/C catalyst for 100 h of continuous H_2_O_2_ production. SEM images indicate that, unlike the powdery Pd/C‐coated electrode with significant catalyst aggregation, the SA–Pd–CS‐coated electrode maintains a uniform, porous surface (Figure S27), mitigating charge‐transfer resistance and suppressing catalyst delamination. During extended operation at 500 mA cm^−2^, the applied voltage for the SA‐Pd‐CS‐coated electrode remained constant, whereas that of the Pd/C‐coated electrode rose by approximately 21% (Figure 4f). Macroscopic SEM images corroborate this divergence: the surface of post‐reaction SA–Pd–CS‐coated electrode stays intact, while the post‐reaction powdery Pd/C‐coated electrode develops extensive cracking (Figure 4f), highlighting the superior stability and mechanical integrity of the SA–Pd–CS coating. Furthermore, TEM images reveal no obvious aggregation of metal species on the SA–Pd–CS‐coated electrode before and after reaction (Figure S28), corroborating the excellent stability of the SA–Pd–CS SAC. Therefore, this soot‐coating strategy offers highly robust and efficient SAC‐GDEs for electrochemical H_2_O_2_ production.

### 2.4 Mechanistic Insights Into Curvature‐Enhanced Activity

To elucidate the origins of the excellent 2e^−^ ORR activity of SA–Pd–CS, density functional theory (DFT) computations were conducted using the fully converged constant‐potential grand canonical method coupled with VASP [32]. Based on the curved microenvironment of SAC sites on candle flame‐derived nanospheres, two representative Pd‐O_3_ motifs were constructed: one on a planar graphitic patch to mimic SAC embedded in a conventional graphene substrate and the other on a bent patch to represent SAC anchored on the curved onion‐like carbon spheres (Figure S28). Bader analysis reveals a greater positive charge accumulated on Pd for the bent model (Figure S29), implying stronger electronic metal‐support interactions between the out‐of‐plane Pd with surrounding oxygen atoms. The strong local polarization of the curved Pd site facilitates adsorption and activation of molecular oxygen, thereby enabling efficient electron transfer to drive the subsequent oxygen reduction reaction.

**FIGURE 5:**
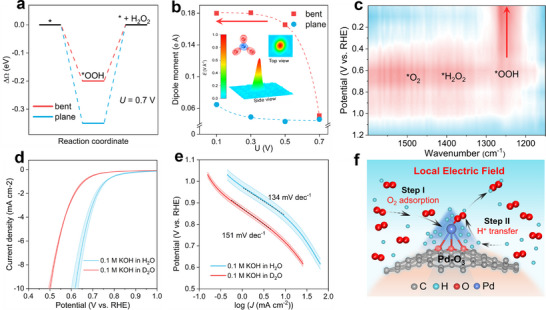
Curvature‐enhanced mechanisms for electrochemical H_2_O_2_ production. (a) Landau free energy diagrams for ^*^OOH adsorption on bent and planar Pd‐O_3_ sites at U = 0.7 V (vs. RHE). (b) Dipole moment of O_2_ adsorbed on bent and planar Pd‐O_3_ sites as a function of applied potential. The inset picture shows the simulated localized electric‐field distribution around an out‐of‐plane Pd‐O_3_ site on a curved carbon sphere. (c) In situ ATR‐SEIRAS spectra of the SA‐Pd‐CS cathodic electrode under various applied potentials. (d) ORR polarization curves of the SA‐Pd‐CS catalyst in alkaline H and D electrolytes, collected with five replicates. (e) The corresponding Tafel plots. The coloured error bands in (d) and (e) represent the standard deviation from the average of five replicates. (f) Illustration of interfacial mass transport and tip‐enhanced localized electric fields that accelerate ^*^OOH formation and H_2_O_2_ production.

We further calculated the Landau free energies for ^*^OOH adsorption at equilibrium potential (*U* = 0.7 V). Both bent and planar Pd‐O_3_ models exhibit thermodynamic binding with ^*^OOH (Figure 5a). However, the bent Pd‐O_3_ site exhibits a higher adsorption affinity for ^*^OOH, leading to higher selectivity for 2e^−^ ORR than the planar counterpart. The calculated H_2_O_2_ overpotential on the bent Pd‐O_3_ SAC is 0.20 V at pH = 0, in close agreement with the experimentally measured value (0.17 V) at pH = 1.1, providing quantitative support for the proposed curvature‐enhanced mechanism and the constant‐potential modeling strategy.

The electric field distribution over the bent Pd‐O_3_ site was also simulated, revealing a tip‐enhanced field localized at the out‐of‐plane Pd‐O_3_ site on the curved carbon sphere (Figure 5b, inset). The variations in the O_2_ dipole moment typically suggest its activation tendency; Figure 5b shows that the O_2_ dipole moment on the bent Pd‐O_3_ site increases sharply to the maximum with the negative shift of the applied potentials (from 0.7 to 0.1 V), whereas the O_2_ dipole moment is much smaller and remains nearly constant on the planar Pd‐O_3_ site, indicating that bent Pd‐O_3_ site can more efficiently activate the adsorbed molecular oxygen. Additionally, the strong local electric field also facilitates proton enrichment, owing to stronger electrostatic interactions [33, 34]. Consequently, the out‐of‐plane Pd‐O_3_ on curved carbon sphere induces a tip‐enhanced local electric field that facilitates both efficient O_2_ activation and interfacial H^+^ accumulation, collectively promoting the formation of the key intermediate of ^*^OOH [34].

To identify and track the evolution of intermediates, we performed in situ attenuated total reflectance surface‐enhanced infrared absorption spectroscopy (ATR–SEIRAS) during electrochemical H_2_O_2_ production using SA‐Pd‐CS‐based GDE (Figure 5c). The ATR‐SEIR spectra revealed a prominent band near 1200 cm^−1^, assigned to the O─O stretching vibration of the adsorbed ^*^OOH intermediate [35, 36]. Not surprisingly, the ^*^OOH signals enhanced with decreasing potentials, which aligns well with the above simulated electric field‐dipole effects, supporting a ^*^OOH‐accumulation pathway toward sustained H_2_O_2_ formation via proton‐coupled electron transfer (PCET, ^*^OOH + H^+^ + e^−^ → H_2_O_2_) [37].

To further probe the PCET processes, we measured ORR polarization curves for the SA‐Pd‐CS catalyst in deuterated (D_2_O) and protic (H_2_O) electrolytes (Figure 5d). Obviously, the ORR polarization curve in D_2_O is shifted to more negative potentials relative to that in H_2_O. Meanwhile, the Tafel slope (TS) is increased from 134 mV dec^−1^ in H_2_O to 151 mV dec^−1^ in D_2_O (Figure 5e). These distinct differences reveal a TS isotope effect during electrochemical H_2_O_2_ production [38], providing direct evidence for the PCET regime. The calculated charge transfer coefficient (*α*) in H_2_O is much greater than in D_2_O over the potential window (Figure S30), further supporting this conclusion. Collectively, the above discussions demonstrate that curvature‐enhanced electric fields promote H_2_O_2_ production over SA‐Pd‐CS by enhancing interfacial mass transfer processes (O_2_ adsorption and local proton concentration) and accelerating interfacial electrochemical reactions (O_2_ activation and ^*^OOH‐accumulation via PCET) (Figure 5f). This proposed mechanism is consistent with the field‐promoted electrocatalysis reported for CO_2_ reduction [39, 40] and hydrogen evolution reactions [33].

### 2.5 Application of SA–Pd–CS GDE

**FIGURE 6:**
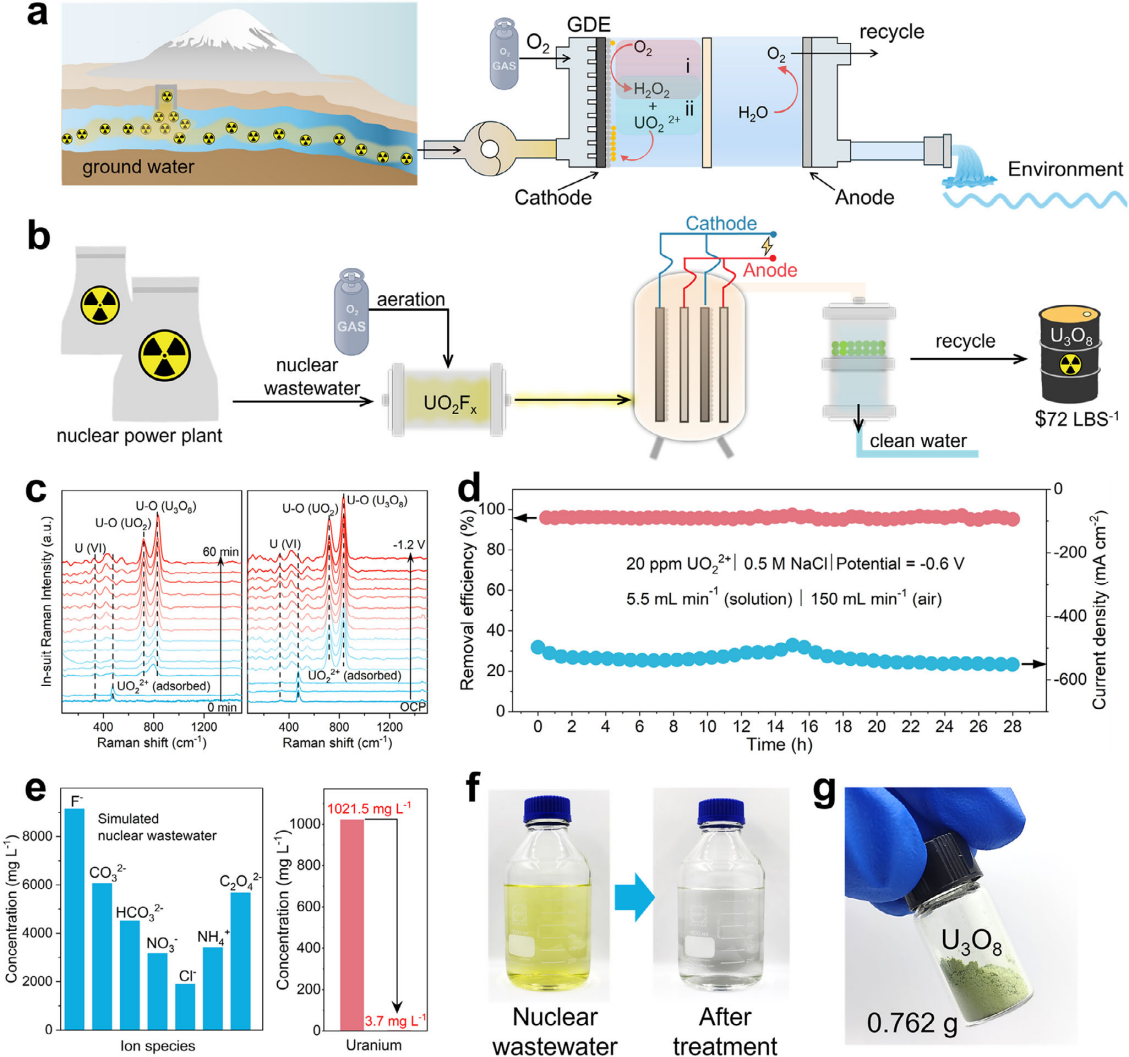
Electrochemical U(VI) removal and recovery using SA‐Pd‐CS coated cathode. (a, b) Schematic of electrochemical cells for treating simulated uranium‐containing ground water (a) and nuclear wastewater (b). (c) In situ Raman spectra during U(VI) removal in 0.5 m NaCl with 20 ppm UO_2_(NO_3_)_2_, tracking potential‐ and time‐dependent evolution of U‐O vibrations. (d) Long‐term continuous‐flow operation for treatment of uranium wastewater at an industrially relevant current density (∼550 mA cm^−2^). (e) Treatment of simulated nuclear wastewater containing high U(VI) and co‐existing ions, with outlet concentration profiles. (f) Photographs of the wastewater before and after treatment. (g) Uranous product recovered from the cathode, purified to uranium oxide (U_3_O_8_).

Coupling with in situ H_2_O_2_ electrosynthesis, the SA–Pd–CS cathode proves effective for treating uranium‐containing waters, including both groundwater (up to tens of mg L^−1^) [39, 40] to nuclear wastewater (up to hundreds of mg L^−1^) [41] (Figure 6a,b). In a two‐electrode system, more than 95% of UO_2_^2+^ (10 ppm) ions were reduced and deposited as a yellow solid on the cathode surface, as evidenced by XRD and SEM‐EDS measurements (Figure S31).

To probe the reaction mechanism for U(VI) reduction, Raman spectra were collected, monitoring signals at different reaction times and applied potentials (Figure 6c). In situ Raman spectra show that U(VI) reduction initiates immediately, even under a very low applied potential (−0.1 V vs. Ag/AgCl), with U‐O vibrational features intensifying over time and with increasing potentials, which is consistent with efficient reduction kinetics. Two complementary pathways are implicated: (i) direct electrochemical reduction of UO_2_^2+^ to UO_2_ on the cathode surface [42], and (ii) H_2_O_2_‐assisted precipitation of U(VI) as insolution (UO_2_)O_2_·4H_2_O via U(VI) + H_2_O_2_ + 4H_2_O → (UO_2_)O_2_·4H_2_O↓ + 2H^+^, enabled by the high‐rate and localized H_2_O_2_ generation at the SA–Pd–CS GDE [43].

We further constructed a flow cell equipped with the SA–Pd–CS‐coated GDE for pilot‐scale wastewater treatment (Figure S31). As shown in Figure 6d, the U(VI) removal efficiency remained above 95% during long‐term continuous operation at a high current density of ∼550 mA cm^−2^, demonstrating robustness for practical remediation. In a single‐cell device treating simulated nuclear wastewater containing high concentrations of U(VI) and co‐existing ions [41], the U(VI) concentrations dropped from 1021.5 to 3.7 mg L^−1^ (Figure 6e), accompanied by a visible color transition from yellow to transparent (Figure 6f). Finally, the collected cathodic precipitate was purified to yield U_3_O_8_ with a 70.4% uranium recovery (Figure 6g), highlighting the potential for resource recovery alongside remediation. Overall, the soot‐deposited SAC GDE platform provides a cost‐effective, durable system for on‐demand H_2_O_2_ production, U(VI) removal, and recycling, thereby enhancing the environmental and sustainability relevance of this technology.

## 3 Conclusions

In summary, we introduced a universal, candle‐soot deposition method that converts atomic metal precursors directly into a conformal SAC coating on diverse electrodes and substrates. This one‐step, scalable route produces a 12‐metal library (and their mixtures) and affords multiscalable control, from precursor‐defined coordination to porosity, wettability, and macroscopic geometry, tailored for gas‐involving electrocatalysis. Engineered hydrophobicity and hierarchical pores enable rapid gas/liquid transport, which curvature‐driven single‐atom motifs concentrate local fields as the active site. As a device‐level validation, SA–Pd–CS GDEs achieve high‐rate, pH‐universal H_2_O_2_ electrosynthesis at industrial current densities, with high selectivity and long‐term stability, outperforming both commercial and SAC powder catalysts. Beyond H_2_O_2_ production, SA–Pd–CS GDEs sustain continuous‐flow U(VI) removal at industrially relevant potentials and enable downstream recovery of solid uranium products, illustrating a novel integrated route to in situ electrosynthesis for simultaneous heavy metal removal and reclamation. Taken together, this soot‐deposited SAC strategy provides a blueprint for robust, high‐catalyst‐loading electrodes, accelerating the translation of single‐atom catalysis from benchtop discovery to deployable devices and processes.

## 4 Experimental Section

### 4.1 SACs Patterning and Coating

Details for chemicals used in this study are provided in the Supporting Information. In the preparation of the M‐candle, 25 g of paraffin wax was first melted in a beaker. Subsequently, a solution of 0.175 mmol of the metal precursor (metallophthalocyanine or metal acetylacetonate) in 2 mL of cyclohexane was added to the molten wax. The mixture was stirred continuously until homogeneous, then transferred into a pre‐wicked candle mold. The filled mold was then cooled to room temperature and demolded to obtain the M‐candle. SA‐M‐CS‐coated electrodes were prepared via a simple one‐step soot‐deposition process. Specifically, electrode substrates (including nickel foam, copper foam, carbon foam, carbon paper, carbon cloth, ITO glass, and graphite plates) were cut into the desired size. The M‐candle was ignited, and the electrode was positioned horizontally approximately 1 cm above the flame for a few seconds. By adjusting the coating duration, SA‐M‐CS‐coated electrodes with varying catalyst loading amounts and thicknesses were obtained. For the fabrication of the SACs pattern, the Cu masking tape was first cut to the desired shape and adhered to the surface of the electrodes (Cu foil, Ni foil, etc.) to form the mask. Subsequently, the masked electrode was subjected to soot deposition over a lit M‐candle. After peeling off the masking tape, a layer of the patterned SAC coating was formed on the electrode surface. For large‐scale fabrication of the SACs coating, multiple groups of lit M‐candles were uniformly placed under the electrodes to deposit SA‐M‐CS SACs across the entire electrode. Cautions: To prevent black smoke emissions into the air, the combustion and soot deposition processes were conducted in a custom‐designed chamber or fume hood fitted with a flue gas purifier. Meanwhile, a mask (N95, N99, or N100) capable of filtering PM2.5 should be worn for protection.

### 4.2 Characterizations and Electrochemical Measurements

XRD patterns were collected using an X‐ray diffractometer (Bruker D8 Advance) at a scan rate of 0.1°s^−1^ in the 2θ range of 5°–90°. SEM images were taken on a ZEISS Gemini SEM 300 at 20 kV. To observe the morphology, SEM analysis was conducted in Secondary Electron Imaging mode. Atomic‐resolution HAADF‐STEM images and the corresponding EDS mappings were taken on a JEOL Grand ARM (JEM‐ARM300F) STEM with double correctors operated at 300 keV. TEM and HR‐TEM images were collected by a JEM‐2100F instrument operated at 200 kV. Raman spectroscopy (Renishaw inVia‐Reflex) was conducted to determine the phase of the material. The metal contents in the SA‐M‐CS cathodes were analyzed using inductively coupled plasma mass spectrometry (Thermo Fisher Scientific). N_2_‐adsorption isotherm measurements were performed on an Autosorb‐I‐MP adsorption instrument (Quantachrome Instruments). XAFS were performed with Si(311) at the b14W1 beamlines at the Shanghai Synchrotron Radiation Facility (SSRF) (Shanghai, China). Details for in situ spectroscopic measurements and electrochemical measurements (CV, EIS, LSV for ORR, HER, and OER) were provided in the Supporting Information.

### 4.3 H_2_O_2_ Yield in the Flow Cell

A flow‐cell device was constructed using a two‐chamber configuration separated by a Nafion 117 membrane. The SA‐Pd‐CS‐coated carbon paper electrode was fabricated via soot deposition. After each coating cycle, 100 µL of ethanol‐Nafion solution (prepared by mixing 980 µL of ethanol with 20 µL of Nafion solution) was applied. The coating process was repeated until the total catalyst mass loading reached 0.5 mg cm^−2^ (electrode area was 1 × 1 cm^2^). After vacuum drying for 12 h, the prepared electrode was applied as the GDE and integrated into the gas‐diffusion flow‐cell system. A platinum electrode (1 × 1 cm^2^) and an Hg/HgO electrode in saturated 0.1 m KOH served as the counter and reference electrodes, respectively. The electrolyte (0.1 m KOH, 100 mL) was circulated through both compartments at a flow rate of 10 mL min^−1^, controlled by a peristaltic pump (ChuXi YZ15). The catalyst was initially activated by cyclic voltammetry (CV) over a potential range of 0.2 to −1 V (vs. Hg/HgO). For the long‐term operation (up to 100 h), the electrolyte (0.1 m KOH) was refreshed every 25 h. The H_2_O_2_ production efficiency of SA‐Pd‐CS‐coated GDE in the flow cell was evaluated in various electrolytes (0.1 m HClO_4_, 0.5 m K_2_SO_4_, and 0.1 m KOH) over a 1‐h operation. The long‐term stability of SA‐Pd‐CS and commercial Pd/C catalysts was evaluated at a current density of 500 mA cm^−2^, using 1 m KOH as the electrolyte. The Faradaic efficiency of the H_2_O_2_ production (*FE_H2O2_*) was calculated using the following equation:

(1) $$FE_{H_{2}}O_{2}=2\mathrm{FCV}/Q$$

where *F* is the Faraday constant (96485 C mol^−1^), *C* is the concentration (mol L^−1^) of produced H_2_O_2_, *V* refers to the volume of electrolyte (*L*), and *Q* represents the consumed charge quantity (*C*). In the chronopotentiometry process, *Q* was calculated according to the following equation:

(2) Q=It

The produced H_2_O_2_ was quantified using the Ce(SO_4_)_2_ titration method. Generally, the yellow Ce^4+^ ions are reduced to colorless Ce^3+^ upon reaction with H_2_O_2_, as described by the following equations:

(3) $$2Ce^{4+}+H_{2}O_{2}\to 2Ce^{3+}+2^{H+}+O_{2}$$

(4) $$\left[H_{2}O_{2}\right]=1/2\Delta \left[Ce^{4+}\right]$$

The concentration of Ce^4+^ ion was determined using UV–vis spectroscopy (Shimadzu, UV 2550). The calibration curve is plotted by fitting the concentration of Ce^4+^ versus its absorbance at 320 nm (Figure S32). By measuring the changes in Ce^4+^ concentrations before and after adding aliquots of the electrolyte collected at specific time intervals, the concentrations of produced H_2_O_2_ in the flow cell can be calculated according to Equation (4).

### 4.4 Electrochemical Uranium Extraction

Electrochemical uranium extraction from wastewater was conducted using a single‐cell (100 mL), three‐electrode system using the SA‐Pd‐CS‐coated Cu foam (catalyst loading: 5 mg cm^−2^, area: 3.5 cm^2^) as the working electrode under a constant potential of −0.6 V vs. Ag/AgCl. A Pt sheet electrode (1.5 × 1.5 cm^2^) served as the counter electrode, and an Ag/AgCl electrode (3.5 m KCl) was used as the reference electrode. For the treatment of 15 L of U(VI) wastewater, a flow cell system was constructed, using the SA‐Pd‐CS‐coated carbon paper as a working electrode with an area of 1 × 1 cm^2^. The counter and reference electrodes were a Pt electrode and an Ag/AgCl electrode, respectively. The electrolyte (0.5 m NaCl) containing 10 ppm U(VI) was circulated at a controlled flow rate of 10 mL min^−1^ using a peristaltic pump (ChuXi YZ15). At desired time intervals, aliquots of the treated solution were collected and analyzed using UV–vis spectroscopy. The concentration of U(VI) was determined with the Arsenazo III spectrophotometric method at a wavelength of 650 nm [44]. The market price of reclaimed uranium was sourced from https://zh.tradingeconomics.com/commodity/uranium on 13 August 2025.

### 4.5 Computational Details

Density functional theory (DFT) calculations were performed using the Vienna Ab initio Simulation Package (VASP) [45, 46]. The electron‐ion interactions were described through the projector augmented‐wave (PAW) method [47], while exchange‐correlation effects were addressed using the generalized gradient approximation (GGA) in the Perdew‐Burke‐Ernzerhof (PBE) form [48]. To improve calculation accuracy, spin‐polarized calculations were performed with on‐site Coulomb interactions incorporated through Hubbard U parameters (U = 2.54) derived from the linear response approach, shown in Figure S33 [49, 50].

The computational models were constructed using a (6 × 6 × 1) graphene‐based supercell. Brillouin zone integrations for structural relaxations were conducted with a Gamma‐centered 3 × 3 × 1 k‐point mesh. The plane‐wave energy cutoff was set to 550 eV, while the convergence criteria for electronic energy and atomic forces were 1×10^−5^ eV and 0.01 eV/Å, respectively. A vacuum space of 25 Å was introduced along the out‐of‐plane direction to suppress image‐image interactions in the periodic cell. Long‐range dispersion forces were described using the DFT‐D3 scheme with Becke‐Johnson damping [51], a method widely employed in recent electrocatalysis studies [52]. To enhance the computational reliability, the “accurate” precision setting was selected to reduce wrap‐around errors.

To simulate electrocatalytic processes under constant potential, we employed the fully converged constant‐potential grand canonical (FCP‐GC) method coupled with VASP [53]. The electrode potential was regulated by changing the electron numbers in the system, while the corresponding compensation charges were treated as point charges using the VASPsol module [54, 55]. In VASPsol, water was chosen as the implicit solvent with a relative permittivity of 78.4. The Debye screening length was set to 3.0 Å, corresponding to an ionic strength of 1 m. Within the constant‐potential framework, the electrochemical potential of electrons is defined by:

(5) $$\mu_{e}=\mu -\left|e\right|U$$

where µ_*e*_ is the electron chemical potential at the potential *U*, and µ is the work function of the standard hydrogen electrode (RHE).

As energy conservation shifts under the grand canonical ensemble, the energy descriptor becomes the Landau potential Ω, expressed as:

(6) $$\Omega =E_{DFT}+ZPE-TS-\mu_{e}N+E_{vib}+E_{tran}+E_{rot}$$

Here, *E_DFT_* is the total DFT energy, and *ZPE* is the zero‐point energy. *TS* corrects for thermal entropy contributions at room temperature (*T* = 298.15 K), while the term µ_*e*_ *N* accounts for the electron contribution. The *S* denotes entropy, and *N* is the number of electrons in the system. *E_vib_*, *E_tran_*, and *E_rot_* are the thermal corrections are due to the vibrational, translational, and rotational energy contributions for free molecules, and only the vibrational contribution is involved in adsorbed intermediates. The second, third, and fifth to seventh terms are mathematically post‐processed using the VASP toolkit [56].

## Author Contributions

X.W. and X.D formulated and supervised the project. Y.L. synthesized and characterized the materials and measured the performances. Y.L. and X.W wrote the original manuscript. K.H. contributed to the atomic phase characterizations. L.L. and Z.C. contributed to the theoretical simulations and mechanisms studies. All authors contributed to the manuscript revision.

## Conflicts of Interest

The authors declare no conflicts of interest.

## Supporting information




**Supporting File**: adma72656‐sup‐0001‐SuppMat.docx.

## Data Availability

The data that support the findings of this study are available from the corresponding authors upon reasonable request. Source data are provided with this paper.
