# Supplementary material for: Scalable and Customizable Single‐Atom Coatings for pH‐Universal H2O2 Electrosynthesis
Source: Adv Mater. 2026 Feb 27;38(18):e21237. doi: 10.1002/adma.202521237 (PMC13014026; doi:10.1002/adma.202521237)
Supplement: Supplementary file 1 — Supporting File: adma72656‐sup‐0001‐SuppMat.docx. [file ADMA-38-e21237-s001.docx]

*Supporting Information*

**Scalable and Customizable Single-Atom Coatings for pH-Universal H_2_O_2_ Electrosynthesis**

*Yu Li^1^, Linguo Lu^2^, Kunsheng Hu^2^,* *Minjia Yan^1^ Xi-Lin Wu^1*^, Zhongfang Chen^2,*^ & Xiaoguang Duan^3,*^*

^1^College of Geography and Environmental Science, Zhejiang Normal University, Jinhua, 321004, China

^2^Department of Chemistry, University of Puerto Rico, Rio Piedras Campus, San Juan, Puerto Rico 00931, USA

^3^School of Chemical Engineering, Adelaide University, Adelaide, SA 5005, Australia

Corresponding authors: Xi-Lin Wu, Zhongfang Chen, Xiaoguang Duan.

Emails: [dbwxl@zjnu.cn](mailto:dbwxl@zjnu.cn); [zhongfang.chen1@upr.edu](mailto:zhongfang.chen1@upr.edu); [xiaoguang.duan@adelaide.edu.au](mailto:xiaoguang.duan@adelaide.edu.au)

**This Supporting Information file includes:**

**58 pages, 35 Figures, 8 Tables, and 115 references**

# **Supplementary Methods**

**Materials**

Vanadium(IV) oxide phthalocyanine (90%), chromium(III) acetylacetonate (97%), iron(II) phthalocyanine (96%), cobalt(II) phthalocyanine (97%), nickel(II) phthalocyanine (95%), copper(II) phthalocyanine (95%), magnesium(II) phthalocyanine (97%), indium(III) phthalocyanine chloride (95%), aluminum phthalocyanine chloride (96%), zinc(II) phthalocyanine (97%), palladium (II) acetylacetonate (98%), cobalt(III) acetylacetonate (98%), Nafion per-fluorinated resin solution (5 wt.% in mixture of lower aliphatic alcohols and water, contains 45% water) were purchased from Sigma Aldrich. Manganese (II) phthalocyanine (98%), ruthenium (III) acetylacetonate (98%), rhodium (III) acetylacetonate (97%), paraffin wax (99%) was purchased from Macklin, Fluorochem, J&K and Merck, respectively. Vitamin B12 (98%), UO_2_(NO_3_)_2_🞄6H_2_O (99%), arsenazo III (99%) were purchased from Aladdin. NaCl (99.8%), NaSO_4_ (99.5%), KOH (85%), HClO_4_ (70.0~72.0%), H_2_O_2_ (70%), Na_2_CO_3_ (99.8%), K_2_SO_4_ (99%), H_2_SO_4_ (98%), K_3_Fe(CN)_6_ (99%), KCl (99.8%), HNO_3_ (65%~68%), HCl (36%~38%), HF (40%), NH_4_F (96%), NaF (99%), NaHCO_3_ (99.5%), Na_2_(C_2_O_4_) (99.8%), Ce(SO_4_)_2_ (99%), chloroacetic acid (99%), sodium acetate (99%) were purchased from Sinopharm Chemical Reagent Co., Ltd.. Commercial Pt/C (20% Pt), Pd/C (20% Pd), ruthenium (IV) oxide (99%), poly(vinylidene fluoride) (PVDF), 2,4-Dinitrophenol (96%) were purchased from Innochem. Carbon foam, nickel foam, Cu foam, carbon paper, carbon cloth, indium tin oxide glass and graphite plate were purchased from Kunshan Lvchuang Electronics Technology Co., Ltd.. Quartz glass sheet purchased from Lianyungang Yunshengda Quartz Technology Co. LtD. Ag/AgCl electrode (3.5 M KCl), Hg/HgO electrode (1 M KOH), Pt sheet electrode RRDE (Ø = 5.61 mm, PINE Research Instrumentation, USA) were purchased from Wuhan Gaoss Union Technology Co., Ltd.. Proton exchange membrane (PEM, Nafion N117), customized flow-cell reactor was purchased from Shanghai Chuxi Industrial Co., Ltd..

**XAFS measurements**

V K-edge, Cr K-edge, Ni K-edge and Zn K-edge XAFS spectroscopy was carried out using the *RapidXAFS* 2M (Anhui Absorption Spectroscopy Analysis Instrument Co., Ltd) by fluorescence mode at 30 kV and 40 mA using the Ge (620) spherically bent crystal analyzer with a radius of curvature of 500 mm. Mn K-edge, Fe K-edge, Co K-edge and Cu K-edge analysis was performed with Si(111) crystal monochromators at the BL11B beamlines and Ru K-edge, Rh K-edge, Pd K-edge and In K-edge analysis was performed with Si(311) at the b14W1 beamlines at the Shanghai Synchrotron Radiation Facility (SSRF) (Shanghai, China). Before the analysis, samples were pressed into thin sheets with 1 cm in diameter and sealed using Kapton tape film. The XAFS spectra were recorded at room temperature using a 4-channel Silicon Drift Detector (SDD, Bruker 5040). Extended X-ray absorption fine structure (EXAFS) spectra were recorded in fluorescence mode. Negligible changes in the line shape and peak position of XANES spectra were observed between two scans taken for a specific sample. The XAFS spectra of standard samples were recorded in transmission mode. XAFS data were processed using the Athena and Artemis programs of three IFEFFIT packages based on FEFF 6^1^. Normalized extended XAFS (EXAFS) data were obtained directly from the Athena program of the IFEFFIT package. The k^2^-weighted WT-EXAFS spectra were generated using Hama software (Morlet function).

***In situ* ATR-SEIRAS and Raman spectroscopy**

*In situ* ATR-SEIRAS measurements were conducted using a Nicolet iS50 FTIR spectrometer equipped with a narrow-band MCT-A detector and an *in-situ* IR optical accessory (SPEC-I, Shanghai Yuanfang Tech.) at an incidence angle of approximately 60°. A CHI 760 electrochemical workstation (CH Instruments, Inc.) controlled the applied potential and measured the current, using a Pt counter electrode and an Ag/AgCl reference electrode. Catalysts were deposited onto a gold-coated silicon wafer, which served as the working electrode. The ATR-SEIRAS spectra were collected stepwise under the applied potential varying from 1.2 V to 0 V *vs.* RHE in an O_2_-saturated 0.1 M KOH electrolyte. For each potential, 70 scans were captured with a spectral resolution of 4 cm^‒1^, and the spectrum at the initial potential of 1.2 V was used for background subtraction.

*In situ* Raman spectroscopy was accomplished with a Raman microscope (Zolix, RTX mini) and a CHI 760 workstation equipped with an electrochemical *in situ* Raman cell. The cell was made of polytetrafluoroethylene with a quartz glass plate to protect the objective lens. To study the conversion process of uranium ions, a working electrode was prepared with SA-Pd-CS, an Ag/AgCl electrode was used as the reference electrode, a platinum wire wrapped around the cell served as the counter electrode, and the electrolyte was 20 ppm UO_2_^2+^ + 0.5 M NaCl. In this case, the working electrode was a glassy carbon electrode with a diameter of 1 cm, and prepared by dropwise addition of prepared SA-Pd-CS ink (5 mg SA-Pd-CS + 1 mL ethanol + 12 μL Nafion, made by sonication for 30 min) to the electrode (20 μL at a time, repeated 5 times, dried with an infrared lamp). Oxygen was passed for 30 min before the reaction and uninterruptedly during the reaction to ensure the oxygen content of the solution. *In situ* Raman spectra were tested for different reaction times (0-60 min, working electrode potential of -0.6 V vs. Ag/AgCl) as well as at different potentials (data collection after 10 minutes of reaction).

**Electrochemical Measurements**

Electrochemical tests were conducted using a CHI 760E (Chenhua Instrument, Shanghai) electrochemical workstation equipped with a three-electrode system. The reference electrode was either an Ag/AgCl electrode or a Hg/HgO electrode, and a Pt sheet electrode served as the counter electrode. The SA-M-CS-coated substrate (10 mm × 10 mm × 1 mm) was applied as the working electrode. For comparison, Cu foam-supported catalysts, including commercial Pt/C (20 wt%), RuO_2_, and Pd/C (20 wt%), were prepared by drop-casting the catalyst ink. The ink was prepared by dispersing 5 mg of powdery catalyst in a mixture solution of ethanol (0.494 mL), water (0.494 mL) and Nafion (12 μL, 5 wt%) and sonicating for 1 h. A 100 μL aliquot of the ink was drop-casted onto a 1 cm^2^ Cu foam substrate, achieving a catalyst loading of 0.5 mg cm^‒2^. EIS measurements were conducted in 0.05 M NaSO_4_, with a frequency range from 10^6^ to 1 Hz. To accurately determine the charge transfer resistance (*R_ct_*), the impedance data were quantitatively analyzed using the equivalent circuit fitting method in “Z-View” software, with the fitting error for each component kept below 5%. The employed equivalent circuit is illustrated in the inset of Figure S21, and the corresponding circuit parameters are summarized in Table S9. This circuit employs the classic Randles–Ershler (RE) model, and the area-specific impedance of the equivalent circuit is given by the following equation:

Z_RE_(ω) = *R_s_* + (jω*C_d_ + 1/(R_ct_+* Z_w_(ω))^‒1^ (1)

Where *cd* is the area specific electric double layer capacitance, Z_w_(ω) is the area specific Warburg impedance *R_ct_* is the area specific charge transfer resistance while *R_s_* is the area specific solution resistance. ECSA was evaluated by determining the capacitance (*C_dl_*) of the catalyst via CV measurements. All potentials were converted to the reversible hydrogen electrode (RHE) scale using the Nernst equation: *E*_RHE_ = *E*_Ag/AgCl_ + 0.0591 × pH + 0.197 V, at 25°C, *E*_RHE_ = *E*_Hg/HgO_ + 0.0591 × pH + 0.098 V, at 25 °C^2^.

ORR Measurements

A RRDE with an active area of 0.2472 cm² was employed as the working electrode. The catalyst ink was prepared by dispersing 5 mg of catalyst in a mixture solution, consisting of 494 μL of water, 494 μL of ethanol and 12 μL of 5% Nafion solution, followed by ultrasonication for 1 h. Subsequently, 6 μL of the ink was applied twice onto the electrode surface, which was then air-dried at room temperature. Prior to the ORR measurements, the RRDE underwent an electrochemical cleaning process by 40 CV cycles at a scan rate of 50 mV s^‒1^ until stable CV profiles were achieved. The collection efficiency *N* was determined using a ferrocyanide/ferricyanide redox couple as described in prior literature^3^. Measurements were performed on the bare RRDE in an oxygen-free electrolyte (1 M KCl + 10 mM K_3_[Fe(CN)_6_]) at various rotation speeds, with the disk electrode potential scanned from 1.1 V to 0.4 V (vs. RHE) at 10 mV s^-1^ to reduce Fe^3+^ to Fe^2+^, while the Pt ring was held at 1.1 V (vs. RHE) to oxidize Fe^2+^ to Fe^3+^. As shown in **Figure S35**, the collection efficiency was calculated to be 0.36, independent of rotation speed, using *N* = –*I_ring_*/*I_disk_*. ORR polarization curves were obtained using linear sweep voltammetry (LSV) at a sweep rate of 50 mV s^‒1^ and a rotation speed of 1600 rpm in an O_2_-saturated electrolyte, with the Pt ring potential maintained at 1.2 V (vs. RHE). The H_2_O_2_ selectivity and the electron transfer number (*n*) were calculated based on the *I_disk_* and *I_ring_* via the following equations (N = 0.36)^4^:

 (2)

 (3)

The Tafel plots were calculated according to the diffusion-corrected kinetic current density (*J_k_*), which was determined through the Koutecky-Levich diffusion formulas as follows^5^:

 (4)

 (5)

In equation (3), *J* refers to the experimental current density, *J_k_* is the kinetic current density, and *J_d_* is the limiting diffusion current density. In equation (5), *n* is the total transfer electron number, *F* indicates the Faraday constant (96485 C mol^−1^), *C_0_* represents saturated O_2_ concentration in 0.1 M KOH, *D_O2_* refers to the diffusion coefficient of O_2_ (1.93×10^−5^ cm^2^ s^−1^), *υ* indicates the kinetic viscosity of the electrolyte (0.01 cm^2^ s^−1^), *ω* is the rotation speed (rad·s^−1^) of the RRDE electrode.

HER and OER Measurements

Prior to HER and OER measurements, the catalysts were first activated through CV at scan rate of 100 mV s^‒1^. Linear sweep voltammetry (LSV) was then performed at scan rate of 5 mV s^‒1^ in N_2_-saturated 0.1 M KOH to minimize the capacitive currents. The electrode potential was automatically iR-compensated with the ohmic resistance. The Tafel slopes were calculated by performing linear fits to the logarithmic current density versus overpotential plots, based on the Tafel equation^6^:

 (6)

where b is the Tafel slope, η is the overpotential, j is the current density, c is the intercept.


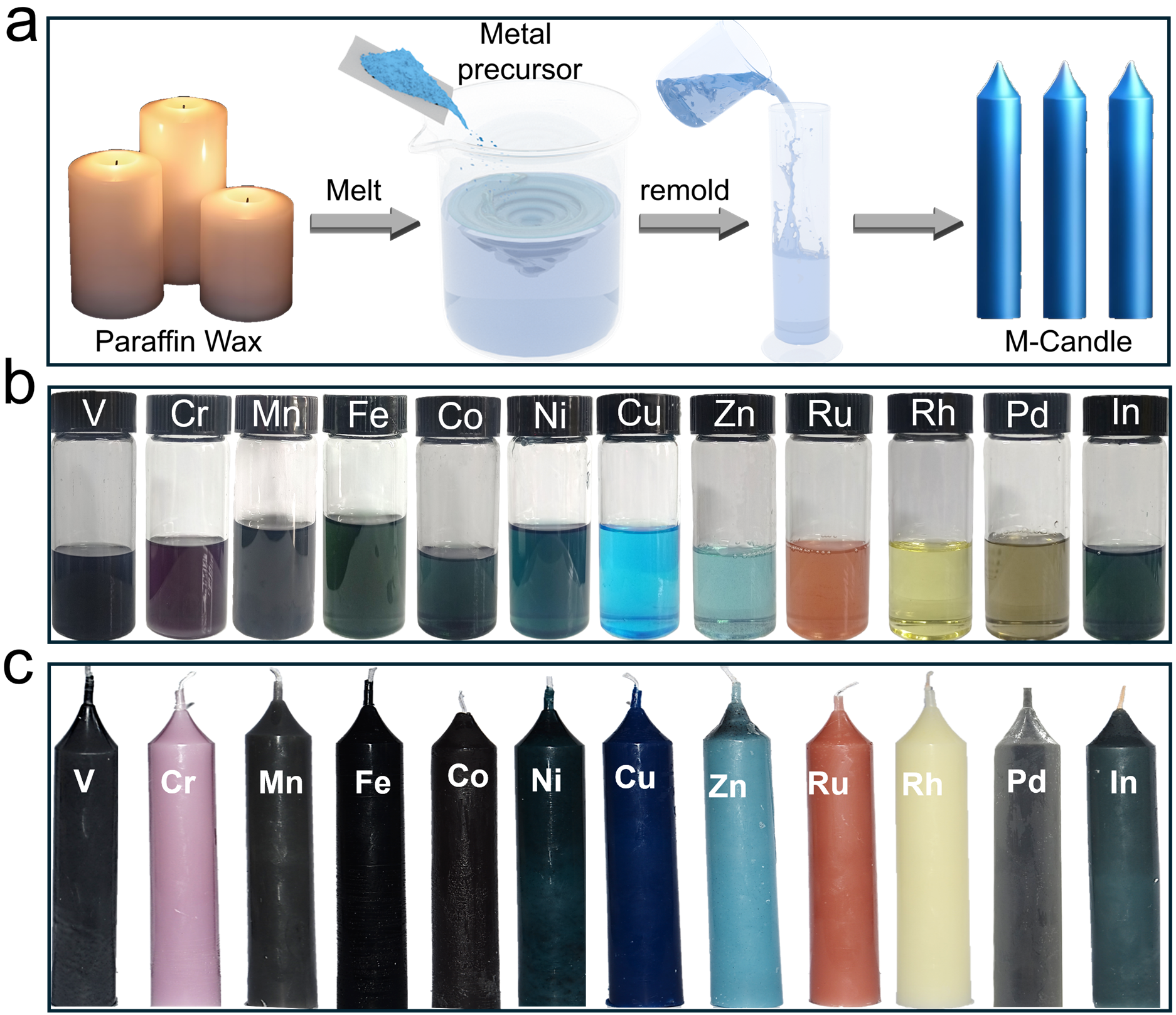


**Figure S****1. Preparation of the M-candles.** a) Schematic of the procedure, b,c) Digital pictures of metal and paraffin wax mixture (**b**) and M-candles (**c**).

**Figure S1a** provides a detailed overview of the M-candles preparation process. Specifically, 25 g of paraffin wax was weighed and heated in a beaker until fully melted. A predetermined amount of metal precursor was added, followed by ultrasonic treatment and stirring to ensure complete dissolution of the precursor in the molten wax, as depicted in **Figure S1b**. The mixture was then poured into candle molds with wicks inserted. After natural cooling, the candles were removed from the molds, yielding the final M-candles, as shown in **Figure S1c**.


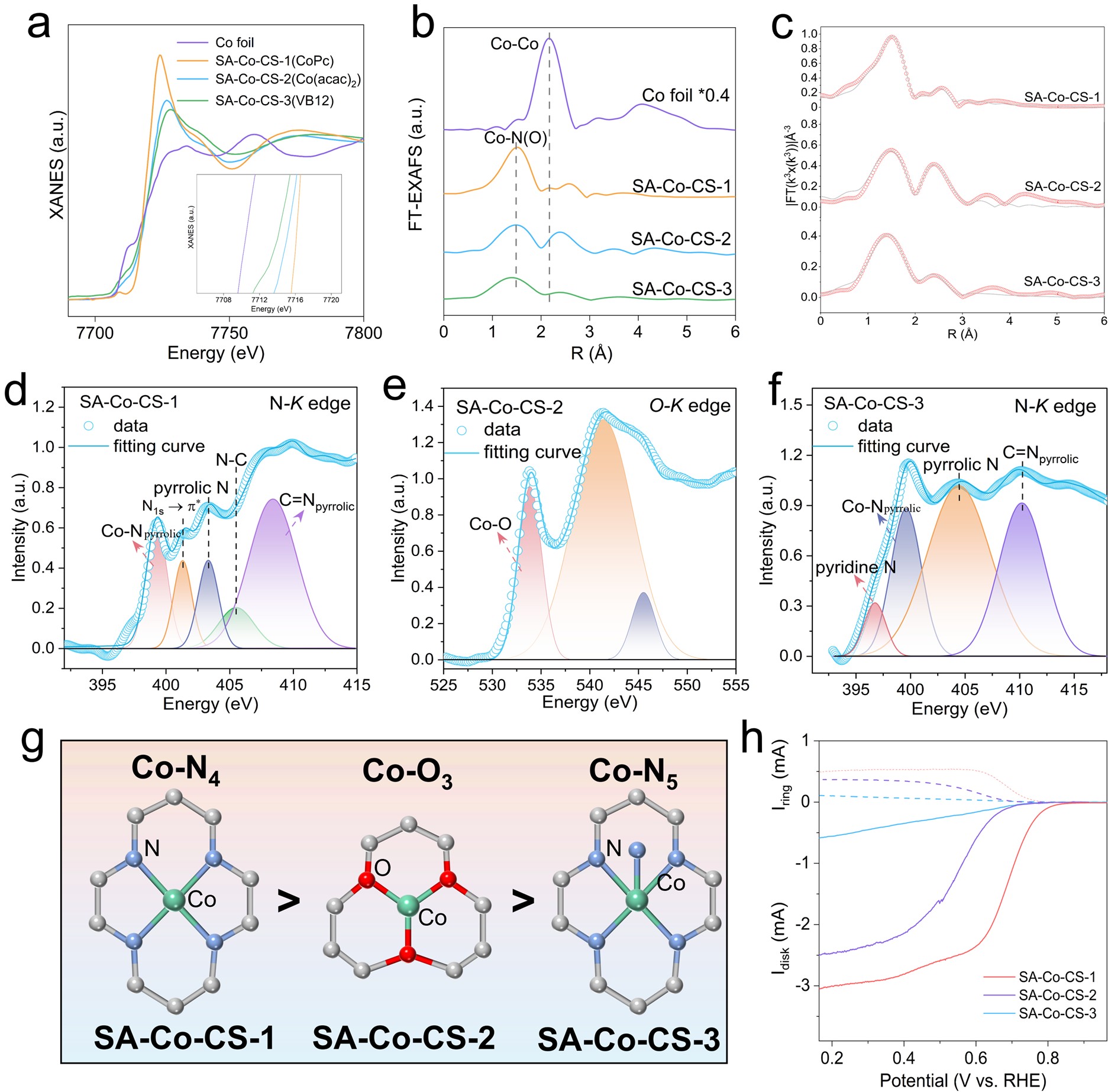


# **Figure S****2. Characterizations and electrochemical performance of the various SA-Co-CS SACs.** a) XAENS spectra, b) XAFS spectra, c) XAFS fitting of the SA-Co-CS SACs, d) N-K edge NEXAFS of SA-Co-CS-1, e) O-K edge NEXAFS of SA-Co-CS-2, f) N-K edge NEXAFS of SA-Co-CS-3, g) LSV curves of the SA-Co-CS SACs for electrochemical oxygen reduction reaction (ORR). h) Coordination structure of the various SA-Co-CS SACs.

SA-Co-CS was used as a representative example to investigate the effect of different metal precursors on the structure and performance of SA-M-CS. SA-Co-CS-1, SA-Co-CS-2, and SA-Co-CS-3 were synthesized using cobalt phthalocyanine (CoPc), cobalt acetylacetonate (CoAc), and vitamin B12 (VB12) as metal precursors, respectively, with a precursor-to-candle mass ratio of 0.007 mmol g^–1^. As shown in **Figure S2a**, the Co K-edge XANES spectra of the SA-Co-CS samples revealed distinct profiles, confirming variations in their valence states and coordination environments. **Figure S2b** demonstrates, through extended X-ray absorption fine structure (EXAFS) analysis, that all three samples exhibit Co-N or Co-O coordination, with no Co-Co metallic bonding detected, indicating the exclusive presence of cobalt in a single-atom form. Differences in R-space peaks further highlight their structural disparities. Fitting results presented in **Figure S2c** show that SA-Co-CS-1 adopts a Co-N_4_ coordination structure, SA-Co-CS-2 exhibits Co-O_3_ coordination, and SA-Co-CS-3 features Co-N_5_ coordination. Furthermore, N- and O-K-edge NEXAFS spectroscopy was employed to rigorously analyze the SA-Co-CS samples. The spectra (**Figures S2d-2f**) revealed distinct fitting peaks corresponding to Co–N(pyrrolic) and Co–O coordinations. This finding directly confirms that the single-atom Co sites successfully retain the coordination structure of their respective Co precursors. Schematic representations of these coordination structures are provided in **Figure S2g**. Finally, the rotating ring-disk electrode (RRDE) curves for the three SA-Co-CS samples, shown in **Figure S2h**, exhibit significant differences. Among them, SA-Co-CS-1, with its Co-N_4_ structure, demonstrates the highest ORR activity.


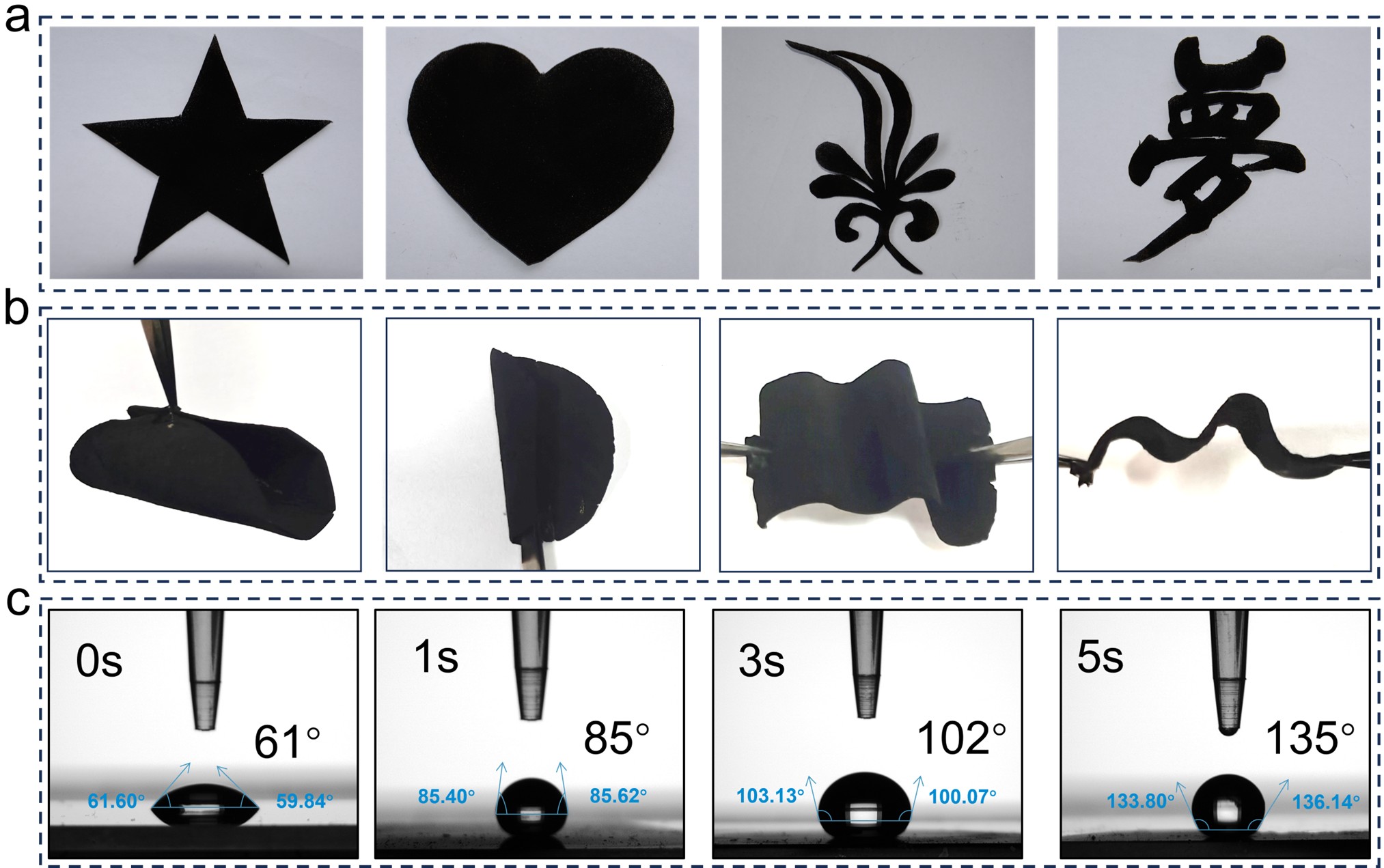


# **Figure S****3. Tunable fabrication of the SACs coating.** a) Cutting the SACs coating into various shapes. b) Flexible SACs coating on PVDF membrane. c) Tunable wettability of the SACs coating with increased soot deposition time.

As illustrated in **Figure S3a**, SA-M-CS coatings can be fabricated into electrodes of various shapes, including stars, hearts, flowers, and the Chinese character for "dream." This versatility makes them adaptable for a wide range of applications. **Figure S3b** presents a flexible membrane prepared using SA-M-CS. The fabrication process is as follows: SA-M-CS was uniformly deposited onto a copper foil substrate using a soot deposition strategy. A specific concentration of PVDF solution (0.05 g/mL) was then applied to fully infiltrate the coating. After vacuum drying for 12 hours, the copper substrate was etched away in 0.5 M H_2_SO_4_, resulting in a flexible, customizable membrane that can be bent and cut as needed.


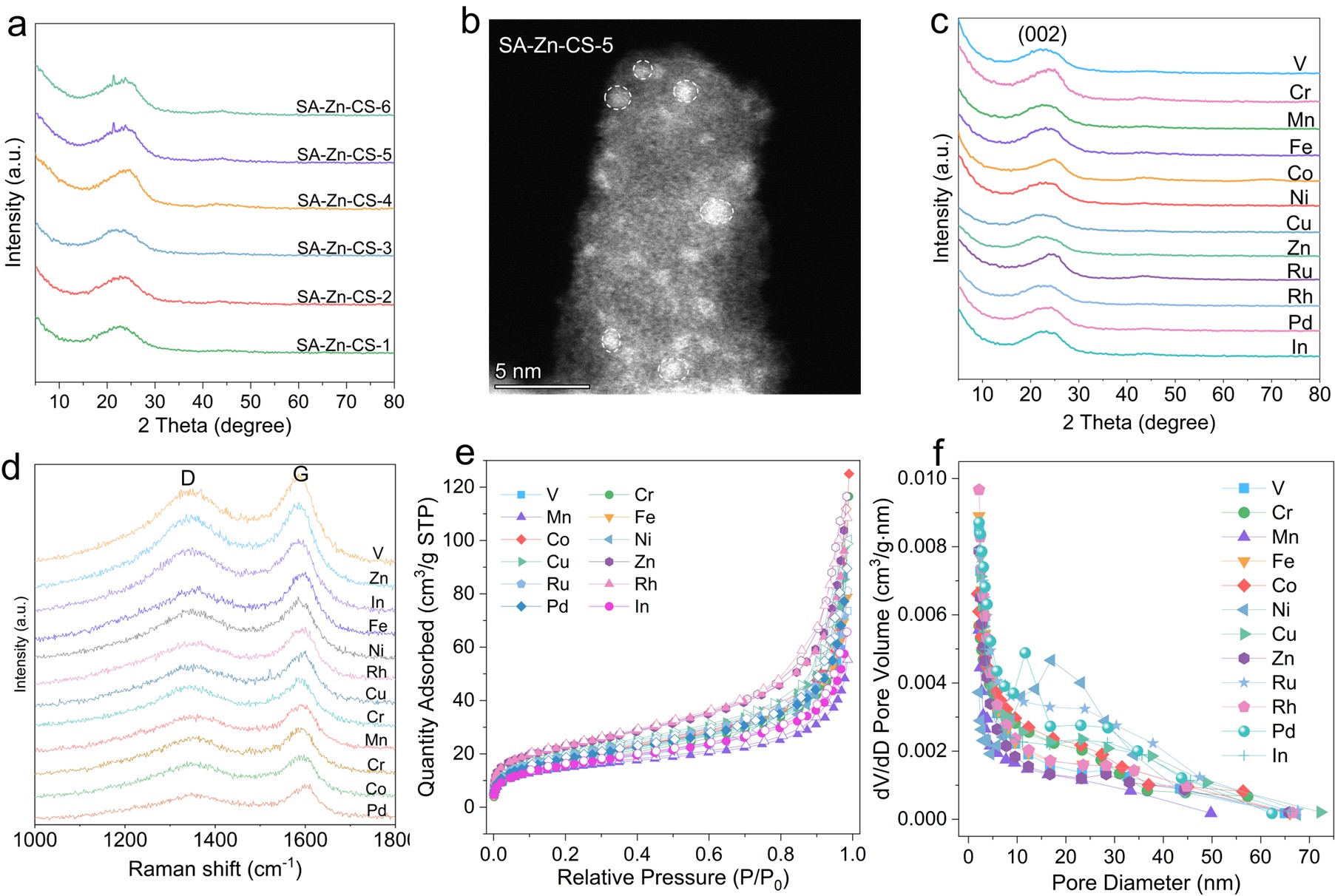


# **Figure S****4. Characterizations of the various SA-M-CS SACs.** a) XRD patterns of SA-Zn-CS samples with various Zn dosages (0.0012, 0.0023, 0.0047, 0.007, 0.0093, 0.0116 mmol g^‒1^ for 1-6 samples, respectively). b) HAADF-STEM image of the SA-Zn-CS-5. c) XRD patterns and d) Raman spectra of the SA-M-CS SACs. e) N_2_ adsorption-desorption curves of the SA-M-CS SACs. f) The corresponding pore size distribution calculated by the BJH method.

As shown in **Figure S4a**, SA-Zn-CS-5 exhibits distinct metallic peaks, and the aberration-corrected electron microscopy image of SA-Zn-CS-5 in **Figure S4b** reveals the presence of noticeable metal clusters. These findings indicate that a precursor concentration of 0.0093 mmol g^-1^ tends to result in metal clusters. Therefore, a lower precursor to candle ratio of 0.007 mmol g^-1^ was selected for the preparation of SA-M-CS. As illustrated in **Figure S4c**, the XRD patterns of 12 different SA-M-CS samples consistently displayed two peaks at approximately 23° and 44°, characteristic of graphitic carbon structures. Notably, no peaks associated with metallic species were detected, verifying the highly dispersed nature of single-atom metals in SA-M-CS. **Figure S4d** highlights the characteristic D and G bands of the graphite phase, which serve as indicators of the graphitization degree of SA-M-CS.


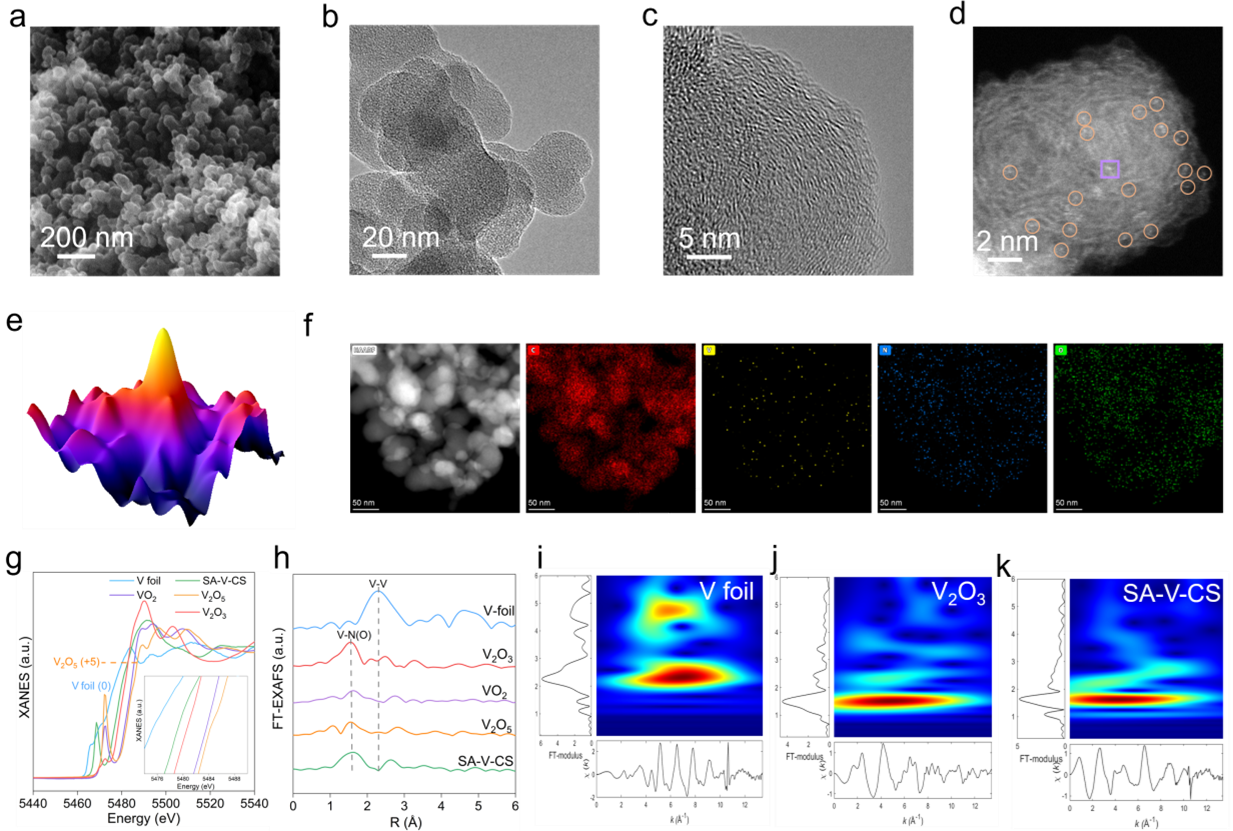


# **Figure S****5. Characterizations of the SA-V-CS.** a) SEM image, b-d) HAADF-STEM images, e) the corresponding atom-overlapping Gaussian-function fitting mapping of the selected square area, f) HAADF-STEM EDS elemental mappings. g) XANES spectra**,** h) XAFS spectra**,** i-k), Wavelet-transformed EXAFS.


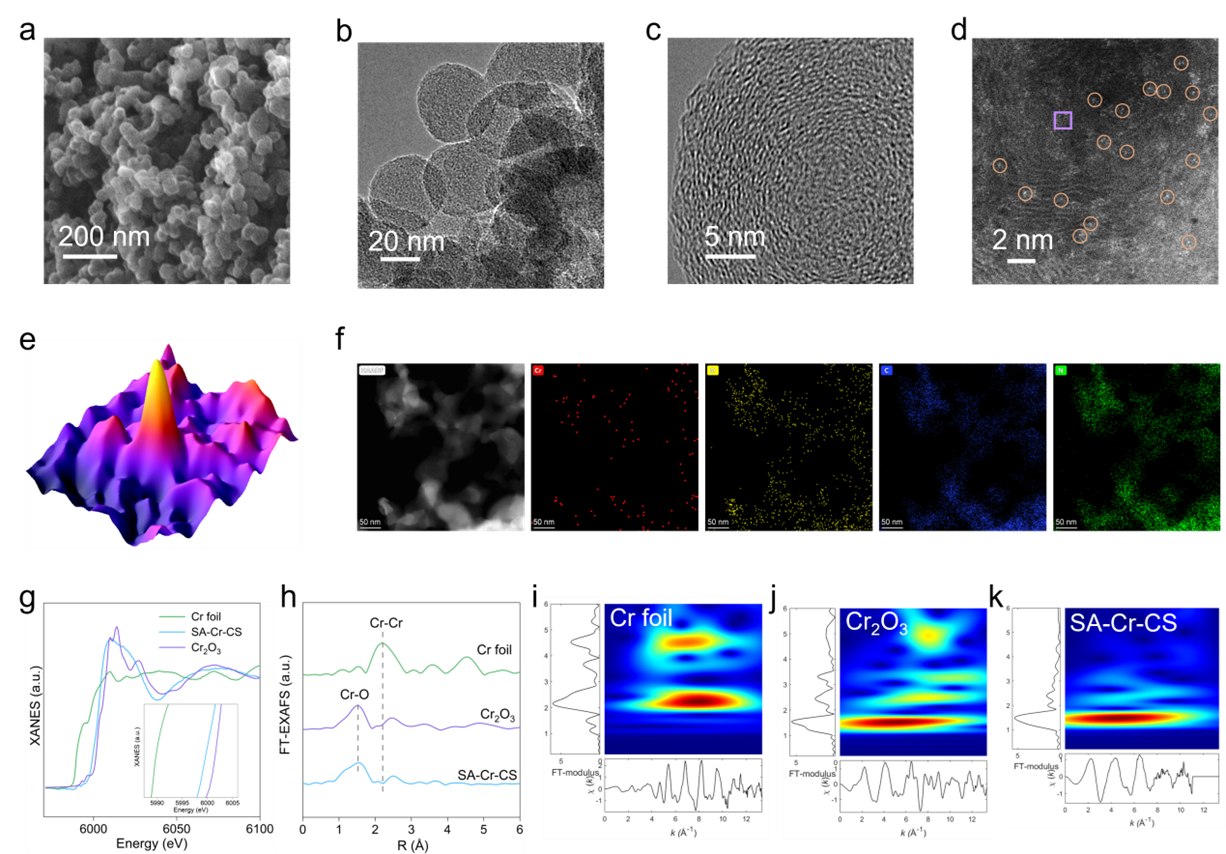


# **Figure S6.** **Characterizations of the SA-Cr-CS.** a) SEM image, b-d) HAADF-STEM images, e) the corresponding atom-overlapping Gaussian-function fitting mapping of the selected square area, f) HAADF-STEM EDS elemental mappings. g) XANES spectra**,** h) XAFS spectra**,** i-k), Wavelet-transformed EXAFS.


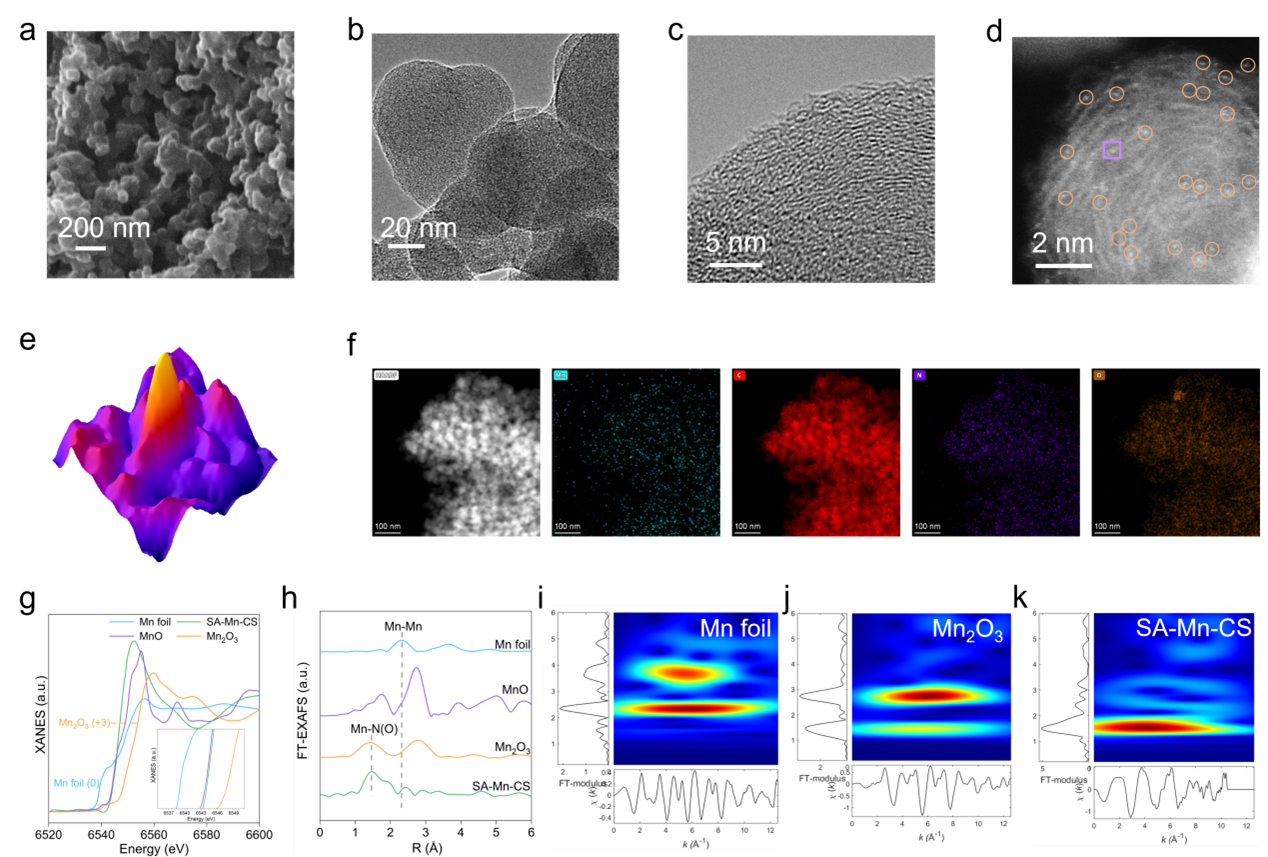


# **Figure S****7. Characterizations of the SA-Mn-CS.** a) SEM image, b-d) HAADF-STEM images, e) the corresponding atom-overlapping Gaussian-function fitting mapping of the selected square area, f) HAADF-STEM EDS elemental mappings. g) XANES spectra**,** h) XAFS spectra**,** i-k), Wavelet-transformed EXAFS.


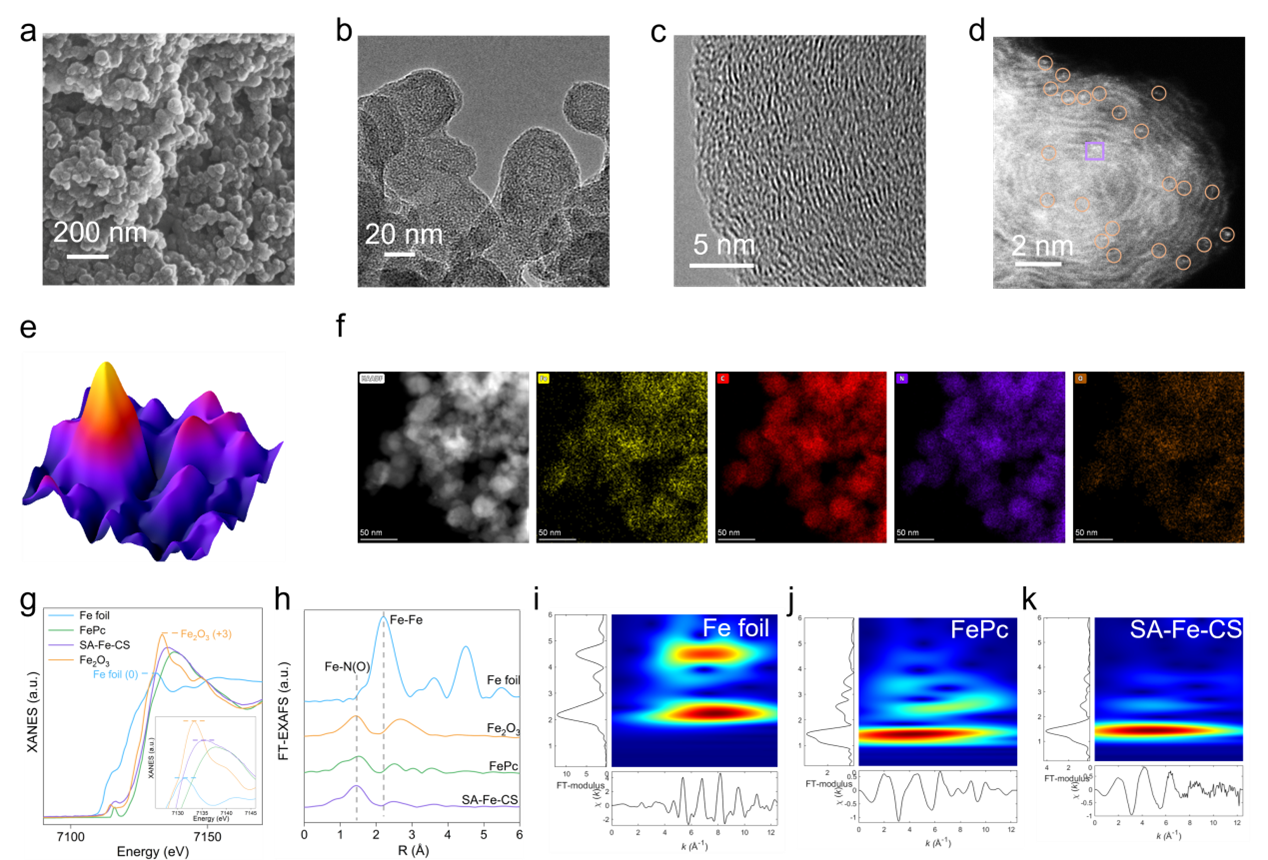


# **Figure S****8. Characterizations of the SA-Fe-CS.** a) SEM image, b-d) HAADF-STEM images, e) the corresponding atom-overlapping Gaussian-function fitting mapping of the selected square area, f) HAADF-STEM EDS elemental mappings. g) XANES spectra**,** h) XAFS spectra**,** i-k), Wavelet-transformed EXAFS.


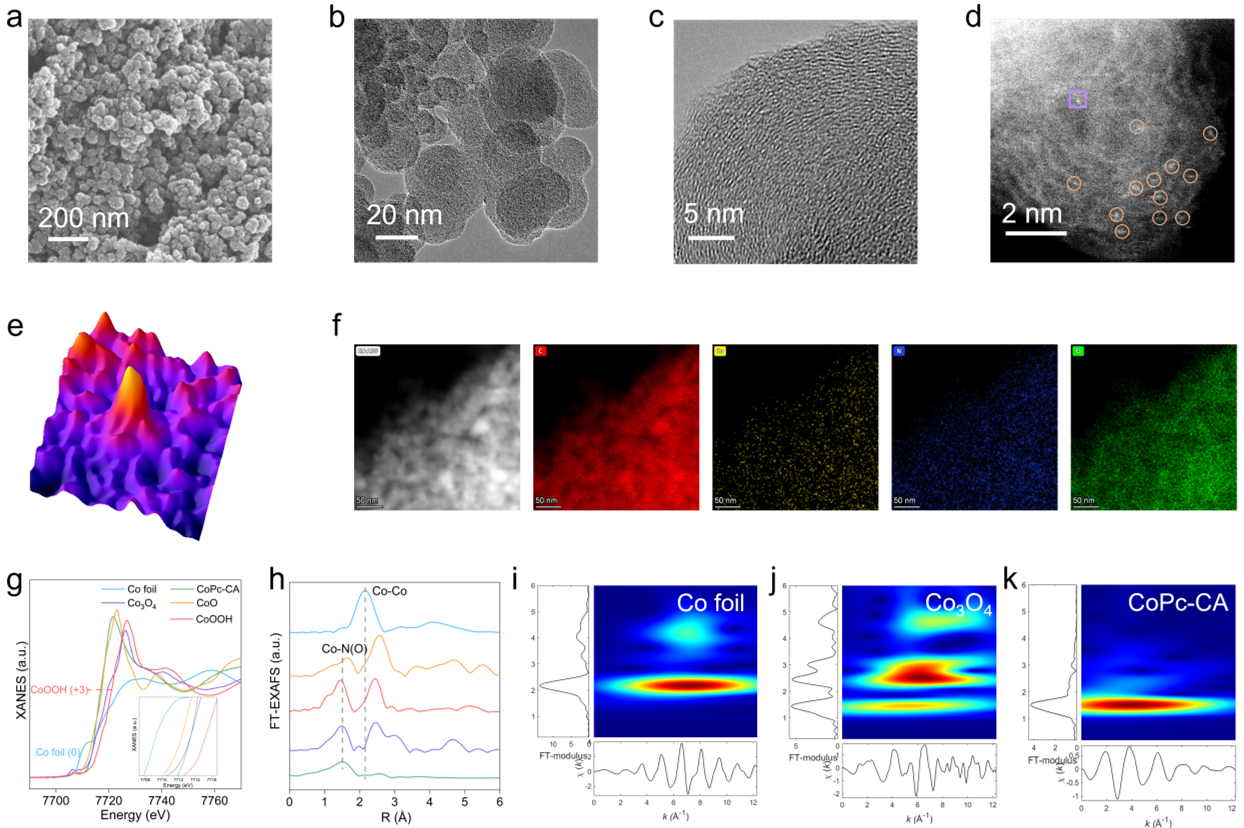


# **Figure S****9. Characterizations of the SA-Co-CS.** a) SEM image, b-d) HAADF-STEM images, e) the corresponding atom-overlapping Gaussian-function fitting mapping of the selected square area, f) HAADF-STEM EDS elemental mappings. g) XANES spectra**,** h) XAFS spectra**,** i-k), Wavelet-transformed EXAFS.


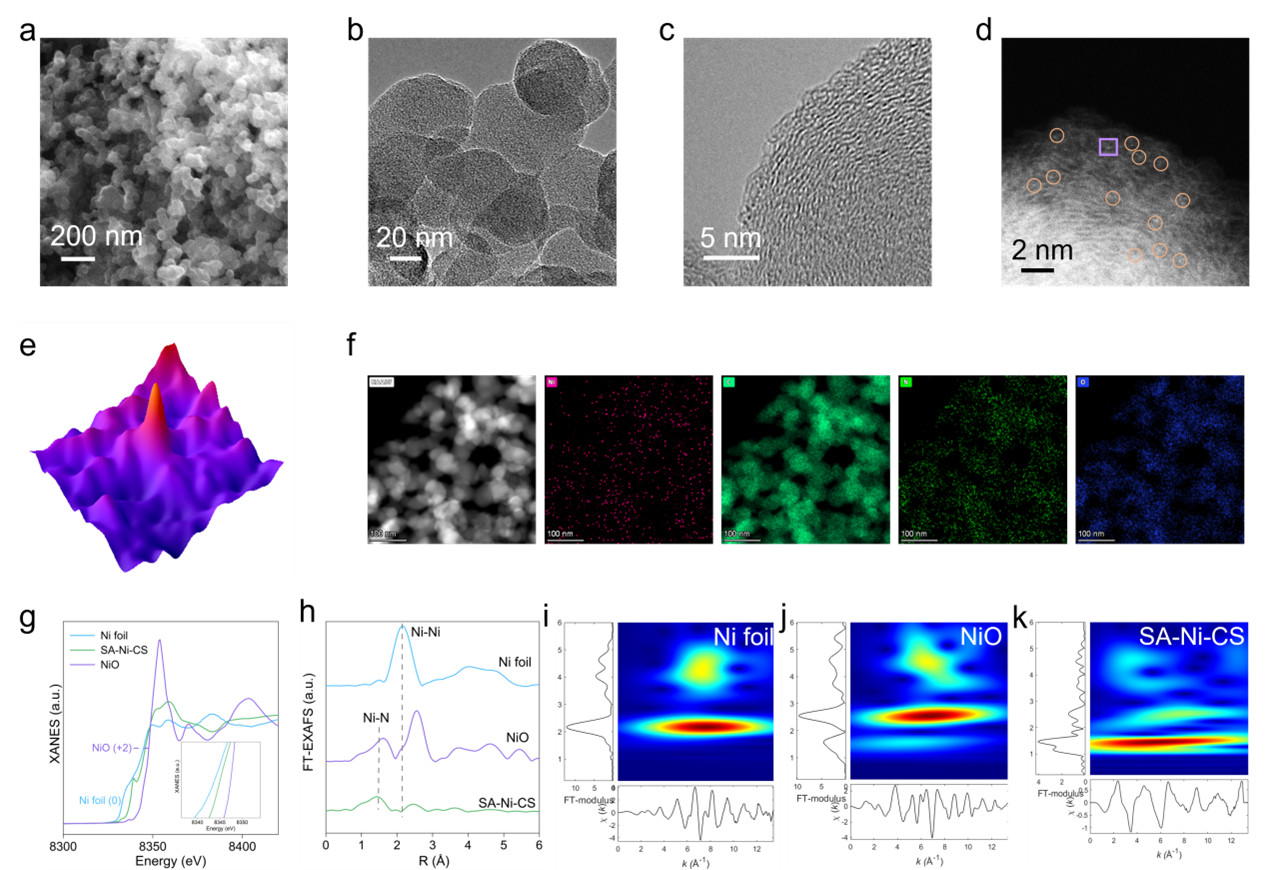


# **Figure S****10. Characterizations of the SA-Ni-CS.** a) SEM image, b-d) HAADF-STEM images, e) the corresponding atom-overlapping Gaussian-function fitting mapping of the selected square area, f) HAADF-STEM EDS elemental mappings. g) XANES spectra**,** h) XAFS spectra**,** i-k), Wavelet-transformed EXAFS.


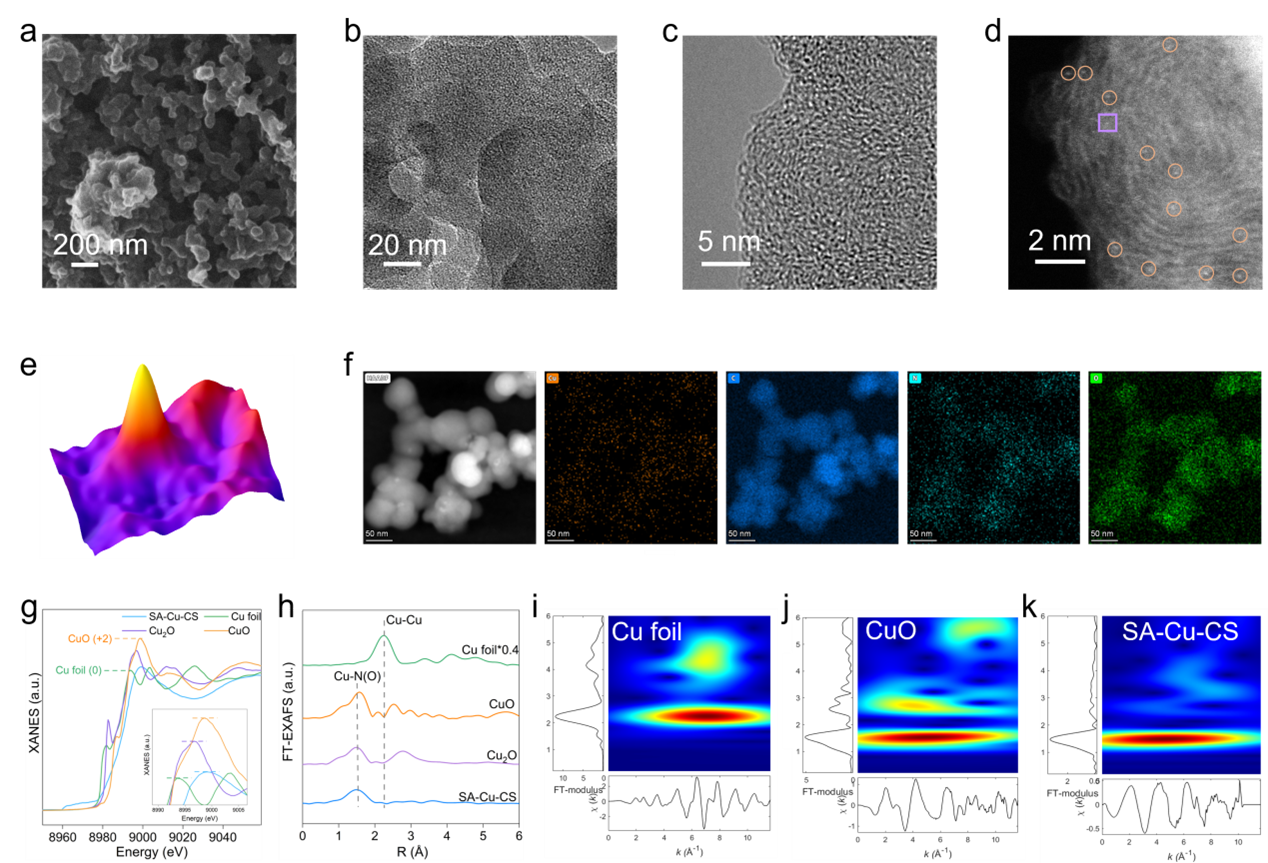


# **Figure S****11. Characterizations of the SA-Cu-CS.** a) SEM image, b-d) HAADF-STEM images, e) the corresponding atom-overlapping Gaussian-function fitting mapping of the selected square area, f) HAADF-STEM EDS elemental mappings. g) XANES spectra**,** h) XAFS spectra**,** i-k), Wavelet-transformed EXAFS.


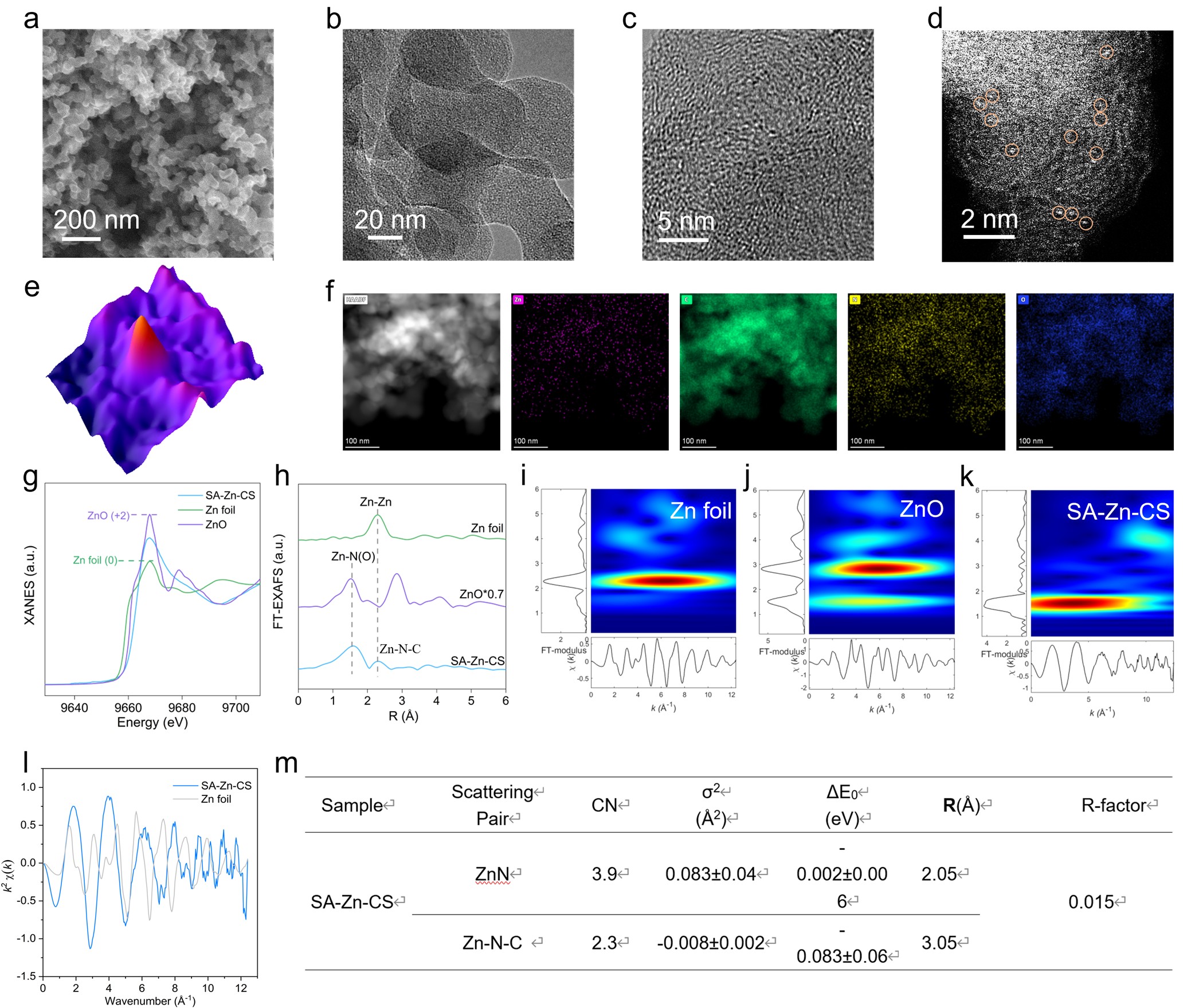


# **Figure S****12. Characterizations of the SA-Zn-CS.** a) SEM image, b-d) HAADF-STEM images, e) the corresponding atom-overlapping Gaussian-function fitting mapping of the selected square area, f) HAADF-STEM EDS elemental mappings. g) XANES spectra**,** h) XAFS spectra**,** i-k), Wavelet-transformed EXAFS. l) *k*^2^-weighted EXAFS χ(*k*) spectra, m) the corresponding EXAFS fitting.

The minor peak observed at approximately 2.2 Å (**Figure S12h**) can be attributed to second-shell scattering from the N–C bonds within the Zn–N–C structure. As shown in the *k*^2^-weighted EXAFS χ(*k*) spectra (**Figure S12l**), Zn foil exhibits a characteristic high-amplitude oscillation in the high-k region (k > 6 Å⁻¹), a feature absent in the SA-Zn-CS sample. Furthermore, EXAFS fitting reveals a Zn–N–C bond length of approximately 3.05 Å, close to that of the typical Zn–Zn bond length (3.247 Å) (**Figure S12m**). An excellent fit (*R*-factor = 1.5%) was achieved using only Zn–N (first shell) and Zn–N–C (second shell) scattering paths, with no Zn–Zn path required. These results collectively provide strong evidence for the presence of isolated single-atom Zn–N₄ sites.


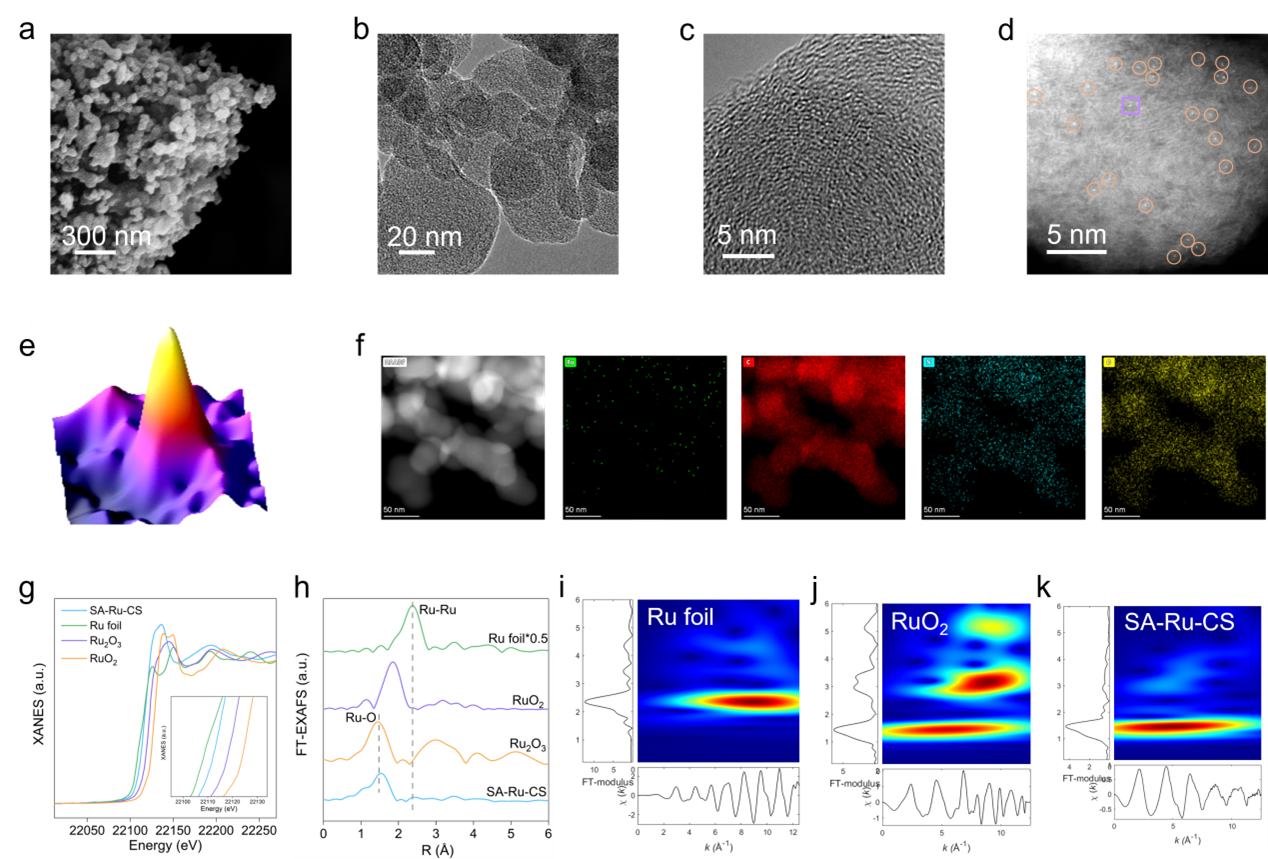


# **Figure S****13. Characterizations of the SA-Ru-CS.** a) SEM image, b-d) HAADF-STEM images, e) the corresponding atom-overlapping Gaussian-function fitting mapping of the selected square area, f) HAADF-STEM EDS elemental mappings. g) XANES spectra**,** h) XAFS spectra**,** i-k), Wavelet-transformed EXAFS.


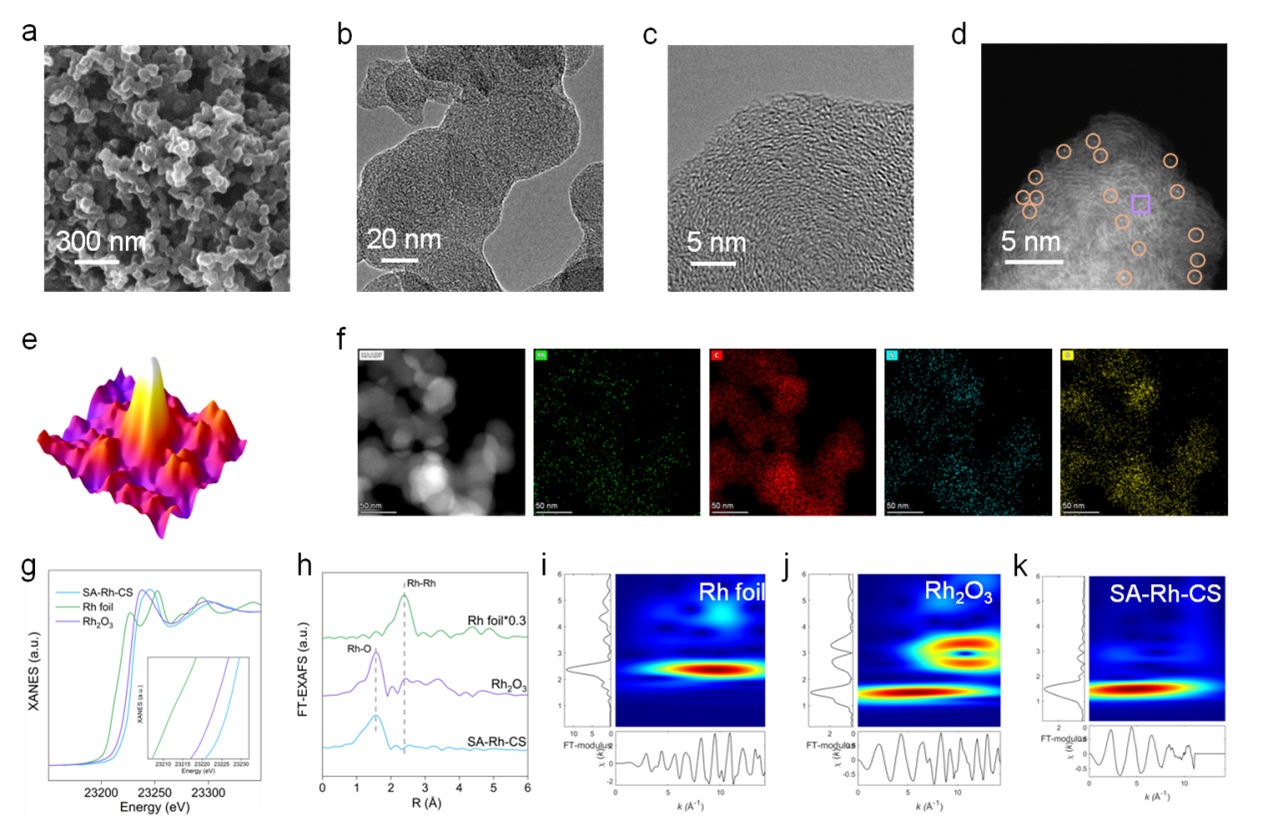


# **Figure S****14. Characterizations of the SA-Rh-CS.** a) SEM image, b-d) HAADF-STEM images, e) the corresponding atom-overlapping Gaussian-function fitting mapping of the selected square area, f) HAADF-STEM EDS elemental mappings. g) XANES spectra**,** h) XAFS spectra**,** i-k), Wavelet-transformed EXAFS.


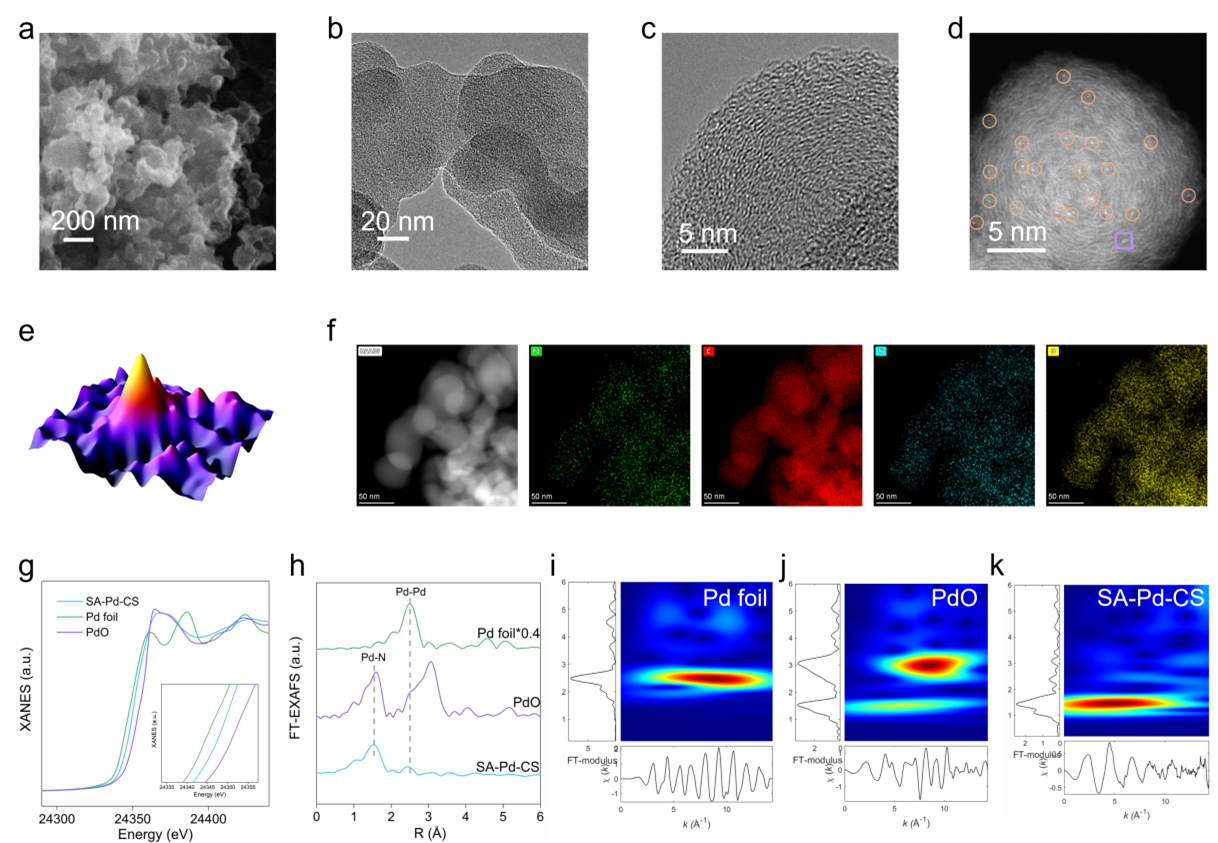


# **Figure S****15. Characterizations of the SA-Pd-CS.** a) SEM image, b-d) HAADF-STEM images, e) the corresponding atom-overlapping Gaussian-function fitting mapping of the selected square area, f) HAADF-STEM EDS elemental mappings. g) XANES spectra**,** h) XAFS spectra**,** i-k), Wavelet-transformed EXAFS.


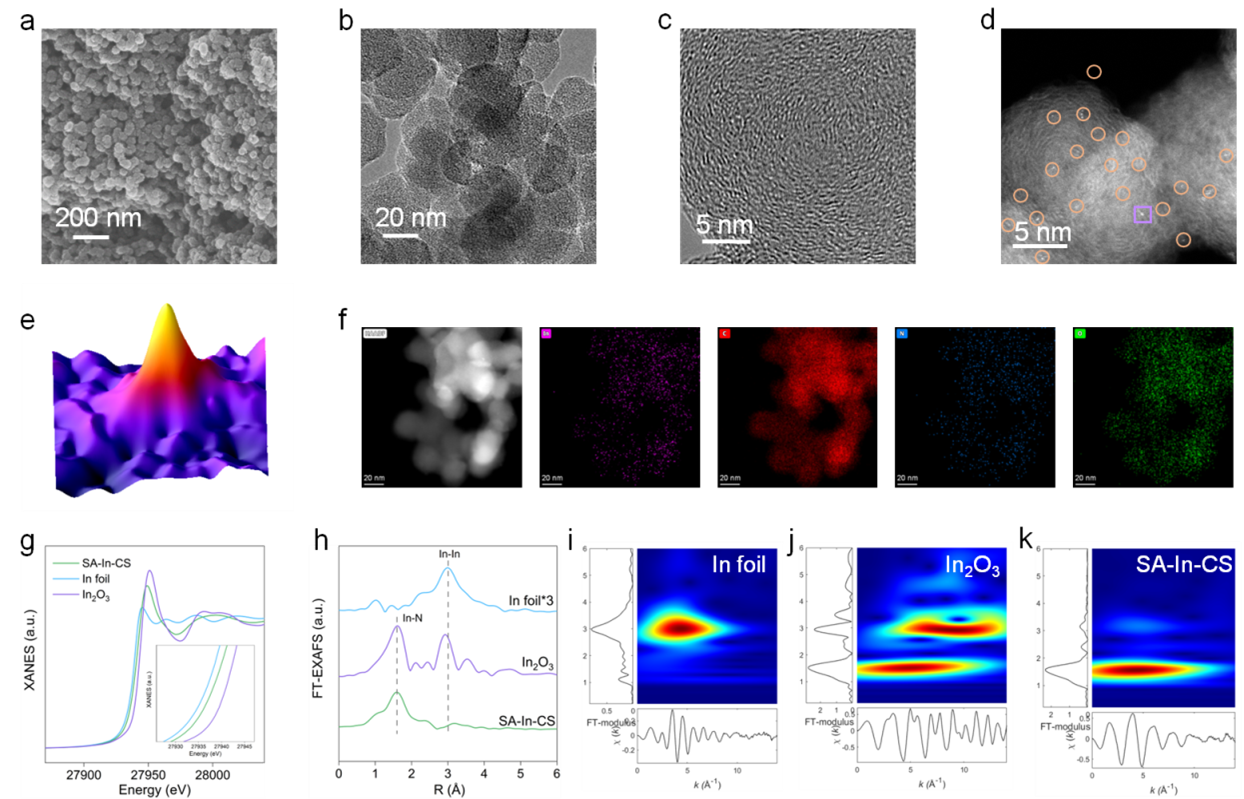


# **Figure S****16. Characterizations of the SA-In-CS.** a) SEM image, b-d) HAADF-STEM images, e) the corresponding atom-overlapping Gaussian-function fitting mapping of the selected square area, f) HAADF-STEM EDS elemental mappings. g) XANES spectra**,** h) XAFS spectra**,** i-k), Wavelet-transformed EXAFS.

**Figures S5a–16a** and **Figures S 5b–16b** present SEM and HAADF-STEM images of SA-M-CS, revealing densely packed nanoscale spheres with an approximate diameter of 40 nm, consistent with previous reports. **Figures S 5c–16c** and **Figures S5d–16d** show no evidence of metal nanoparticles or clusters in the HAADF-STEM images. Instead, a uniform graphite layer is observed, along with distinct atomic-scale bright spots, indicating the presence of metals in a single-atom form (3D visualizations of these atomic bright spots are shown in **Figures S5e–16e**). EDS elemental mappings (**Figures S5f–16f**) confirm the uniform distribution of metal atoms across the carbon support. Further analysis using XAFS provides insights into the coordination structure of SA-M-CS. **Figures S5g–16g** indirectly reveal the valence states of the metals, while **Figures S5h–16h** offer details on the coordination environment, although distinguishing between light elements such as O and N remains challenging. Wavelet transform analysis (**Figures S5i–16i**) enables further differentiation between N and O coordination.


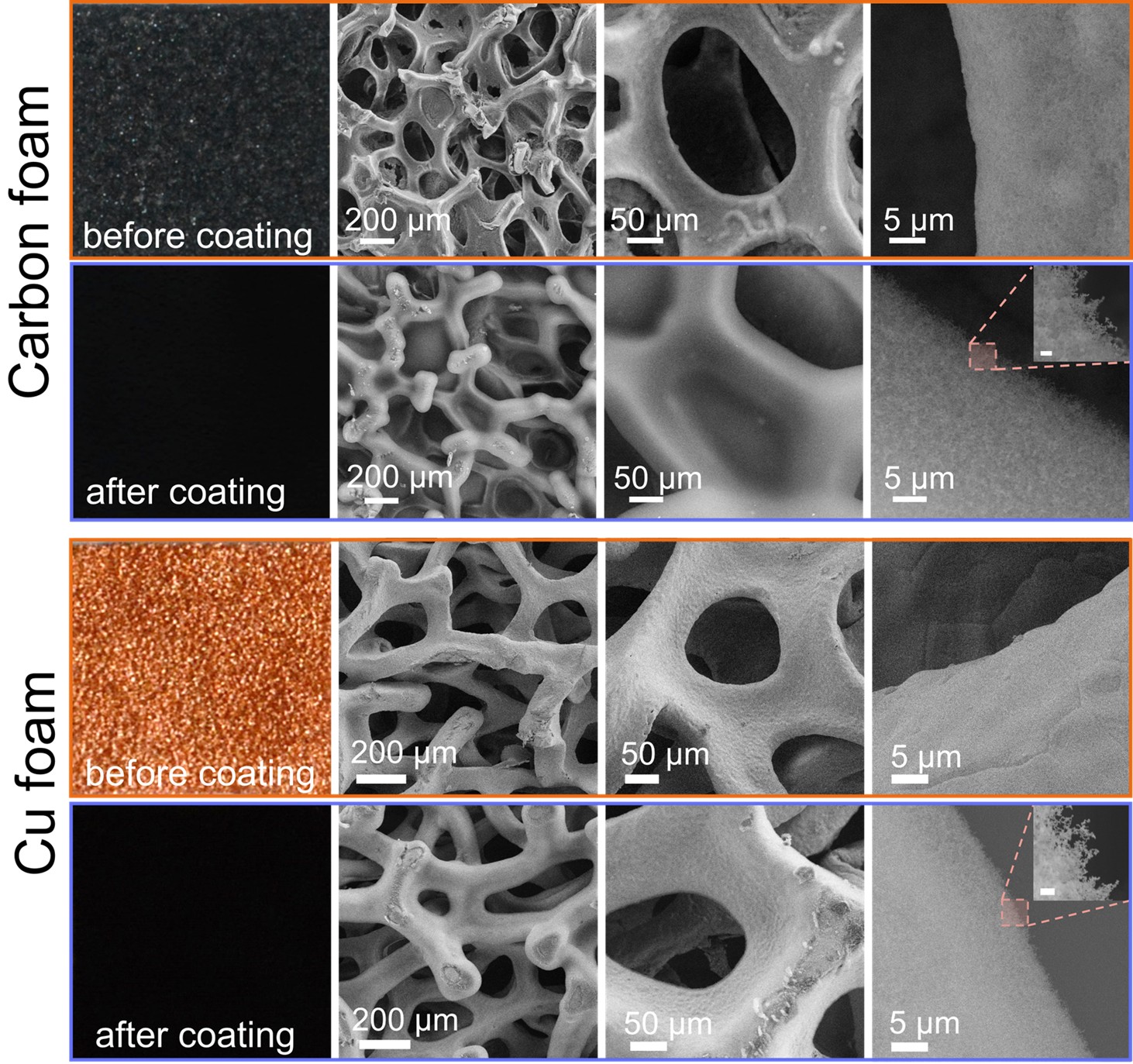


# **Figure S****17. Digital pictures and SEM images of the SACs coating on carbon foam and Cu foam.**


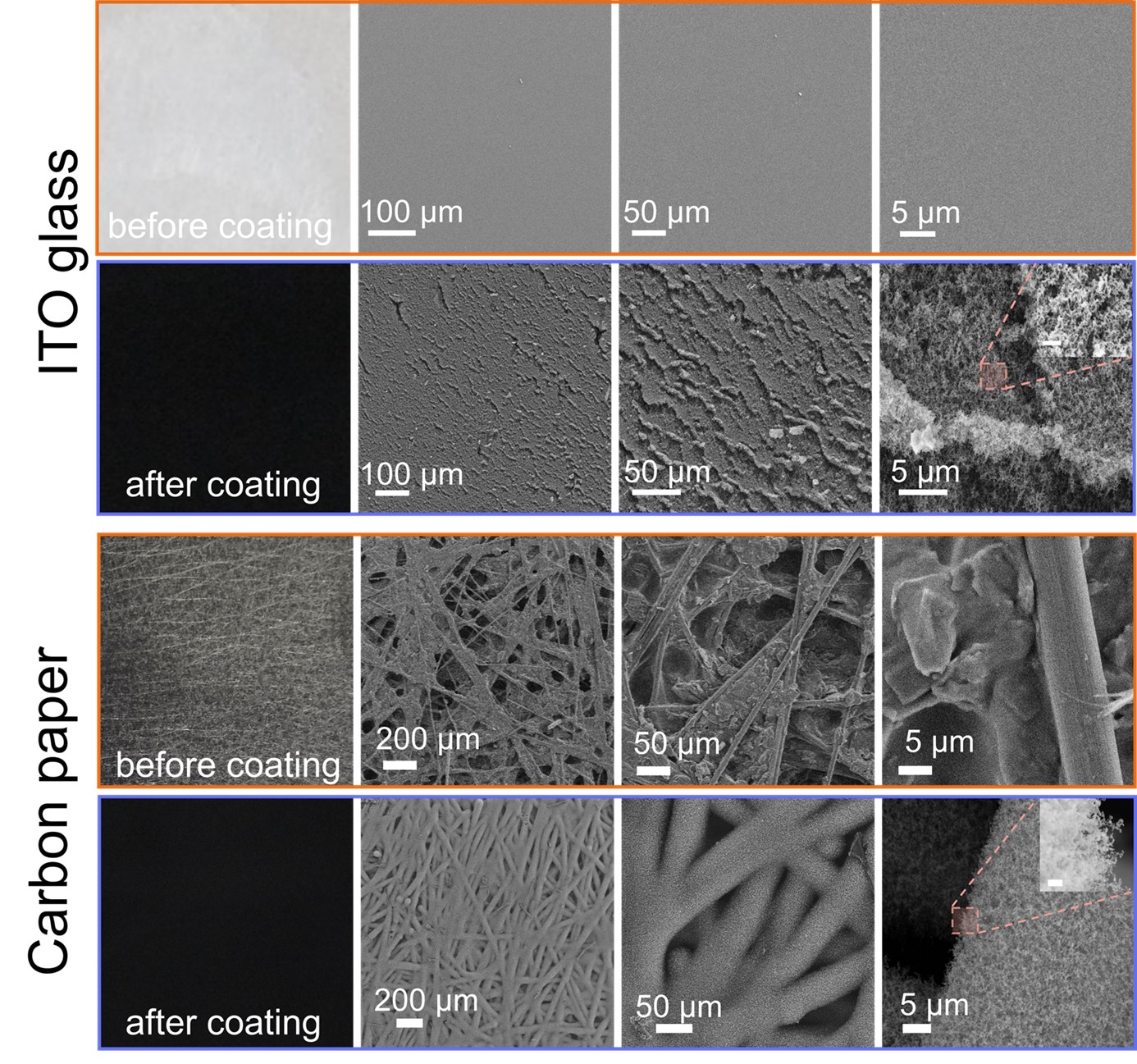


# **Figure S****18. Digital pictures and SEM images of the SACs coating on ITO glass and carbon paper.**


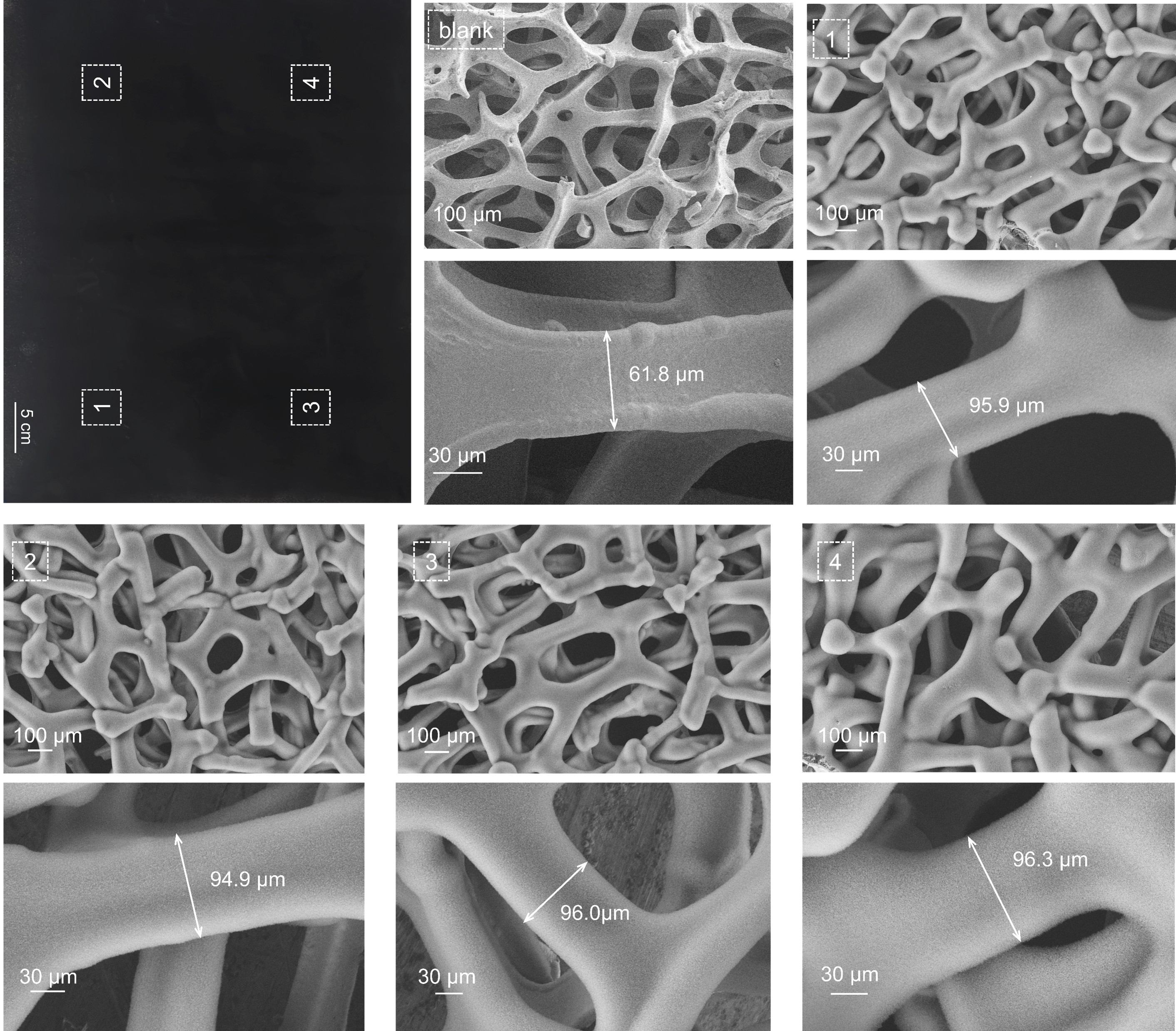


# **Figure S19. Digital picture of the SAC coating on the large-area Cu foam and the corresponding SEM images of the selected region.**


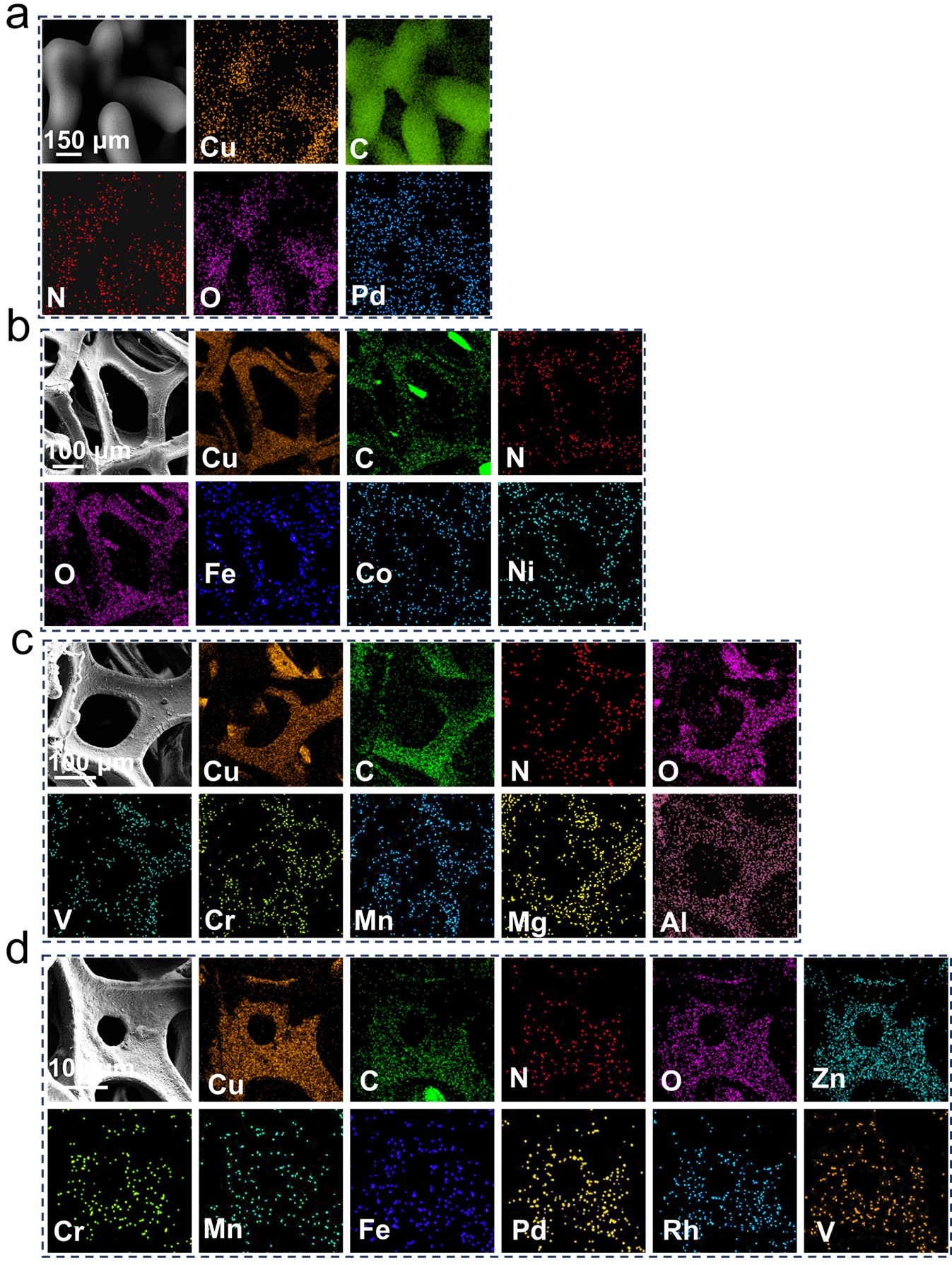


# **Figure S****20. SEM-EDS mapping of the various SACs coating.** a) (Pd) Unitary SACs coating, b) (Fe, Co, Ni) ternary SACs coating, c) (V, Cr, Mn, Mg, Al) quinary SACs coating, d) (Zn, Cr, Mn, Fe, Pd, Rh, V) septenary SACs coating.


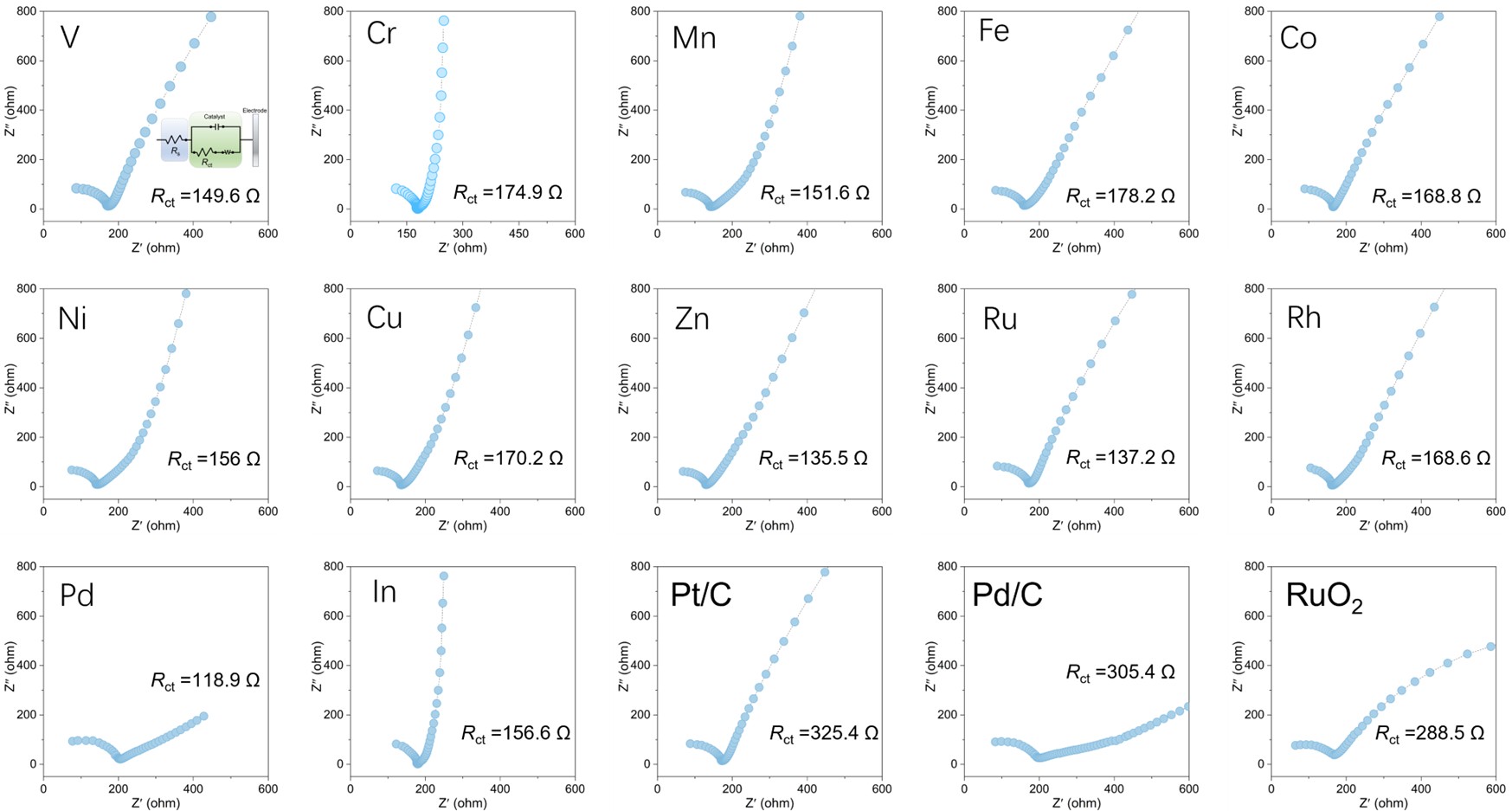


# **Figure S****21.** **Impedance measurements of the various SA-M-CS.**

The Nyquist plots obtained from EIS measurements are presented in **Figure S21**, with the inset showing an equivalent circuit model used for data fitting via "Z-view" software. The equivalent circuit depicted in the inset of the SA-V-CS Nyquist plots includes *R_s_* and *R_ct_*. *Rs*, representing the electrolyte resistance, accounts for the resistance to ion conduction within the electrolyte solution. Its value is influenced by factors such as ion concentration, conductivity, temperature, and the geometric properties of the electrodes. *R_ct_*, or charge transfer resistance, describes the resistance to charge transfer at the electrode/electrolyte interface. Each *Nyquist* plot consists of two distinct parts. The intercept with the real axis at high frequencies corresponds to *R_s_*. The calculated *R_ct_* values range from 110 to 180 Ω, much lower than those of the commercial catalysts (Pt/C, Pd/C, RuO_2_). The plots exhibit incomplete semicircles at high frequency and a straight line at low frequency, indicating that the electrochemical system is governed by both charge transfer and diffusion processes^7^.


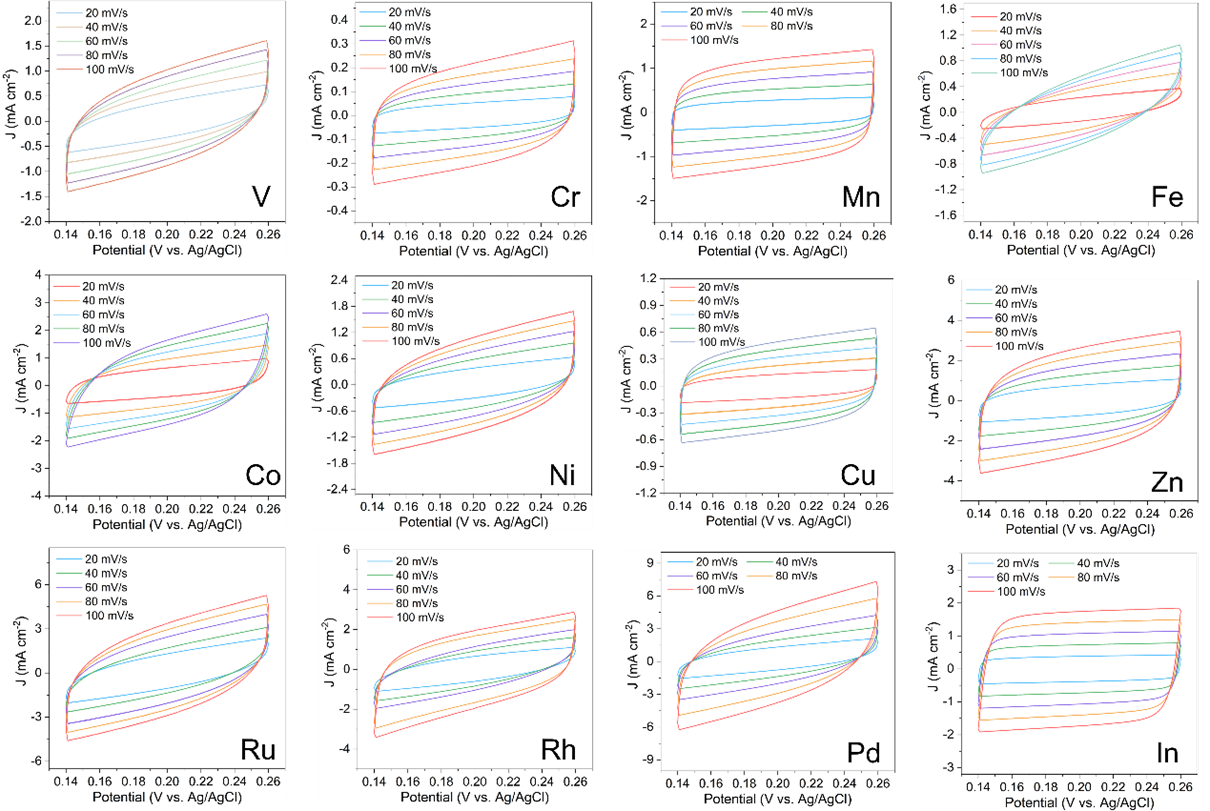


# **Figure S****22. CV curves of the SA-M-CS SACs at various scan rates.**


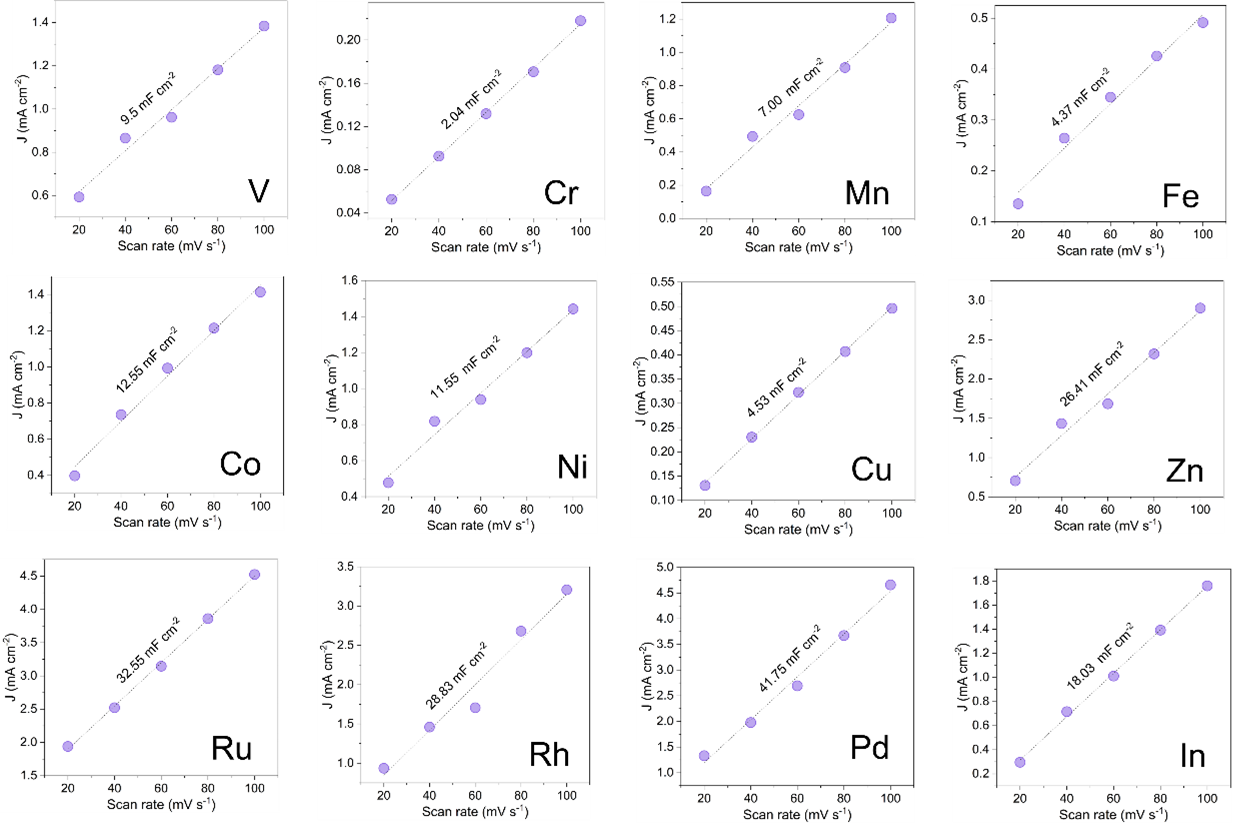


# **Figure S****23. Calculation of the ECSA values of the SA-M-CS SACs by fitting scan rates versus current of the CV measurements.**

To evaluate the effective electrochemical surface area (ECSA) during catalysis, cyclic voltammetry was employed to estimate the double-layer capacitance (*C_dl_*). **Table S4** compares the ECSA of single-atom catalysts reported in recent publications this year, highlighting that SA-M-CS exhibits a significantly larger ECSA, thereby offering more catalytically active sites.


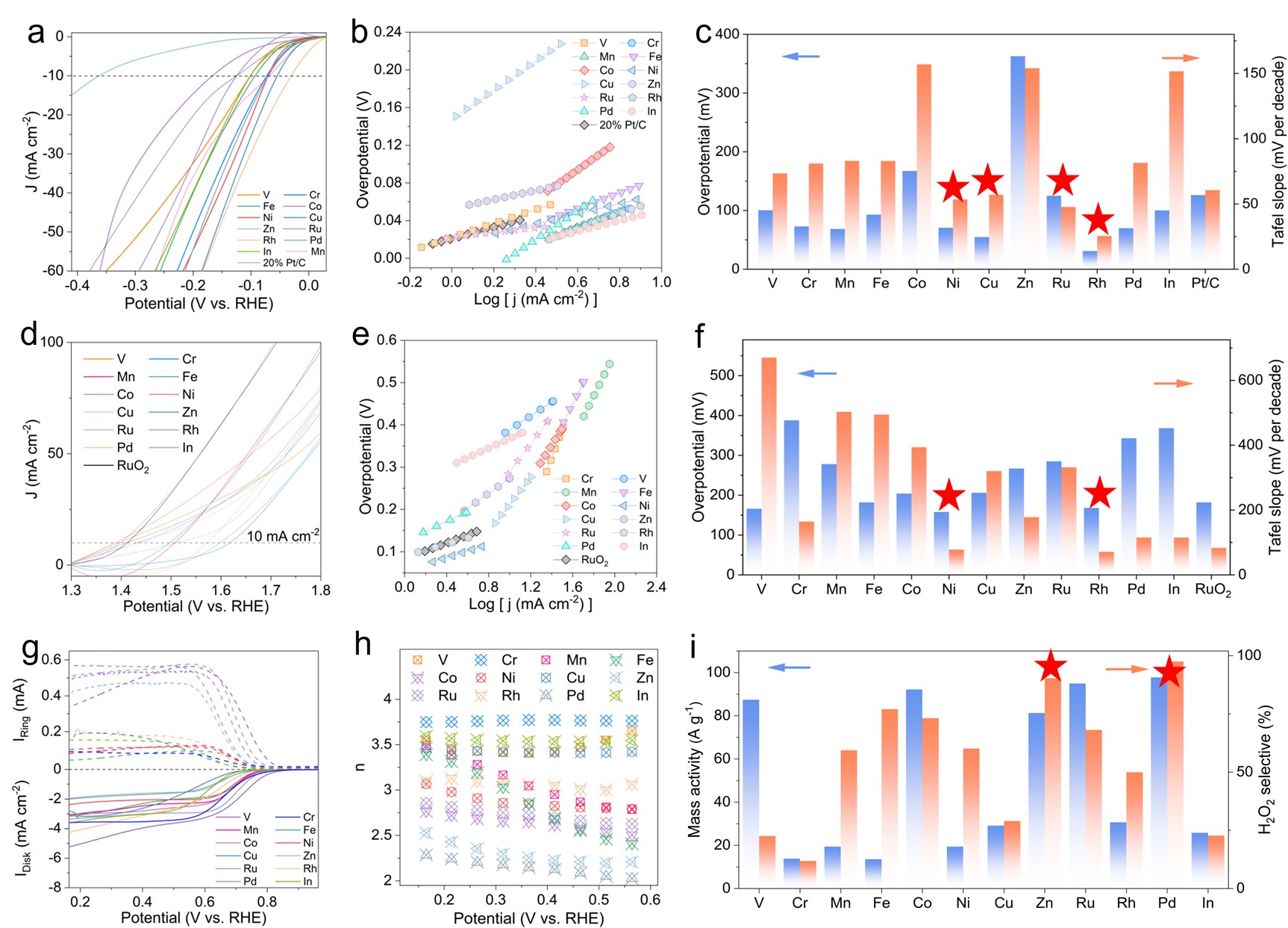


# **Figure S****24. Performances of the SA-M-CS SACs for electrocatalysis.** a-c) HER (in 0.1 M KOH), d-f) OER (in 1 M KOH) and g-i) ORR (in 0.1 M KOH) measurements.

The results in **Figures S23 a–c** demonstrate that SA-Ni-CS, SA-Cu-CS, SA-Ru-CS, and SA-Rh-CS exhibit comparable or lower HER overpotentials and Tafel slopes than the commercial Pt/C catalyst, confirming the superior HER activity of SA-M-CS. **Figures S23 d–f** shows that SA-Ni-CS and SA-Rh-CS achieve low OER overpotentials and Tafel slopes, comparable to commercial RuO_2_. Interestingly, SA-Ni-CS and SA-Rh-CS exhibit outstanding performance in both HER and OER, making them promising candidates for overall water splitting applications. **Figures S23 g–i** examine the ORR performance of SA-M-CS (tested in powder form). The results reveal distinct ORR pathways among the catalysts: SA-V-CS, SA-In-CS, and SA-Cu-CS favor a 4e^–^ ORR pathway, while SA-Ni-CS, SA-Ru-CS, and SA-Rh-CS tend towards a 3e^–^ pathway. Meanwhile, SA-Zn-CS and SA-Pd-CS predominantly follow a 2e^–^ ORR process. The 4e^–^ ORR is ideal for applications such as fuel cells and metal-air batteries^8^, while the 3e^–^ ORR facilitates in situ hydroxyl radical production for water treatment^9^. The 2e^–^ ORR is highly desirable for H_2_O_2_ production^10^. Additionally, comparisons of ORR limiting diffusion current density and H_2_O_2_ selectivity confirm that SA-Pd-CS and SA-Zn-CS exhibit the highest ORR activity and H_2_O_2_ selectivity.


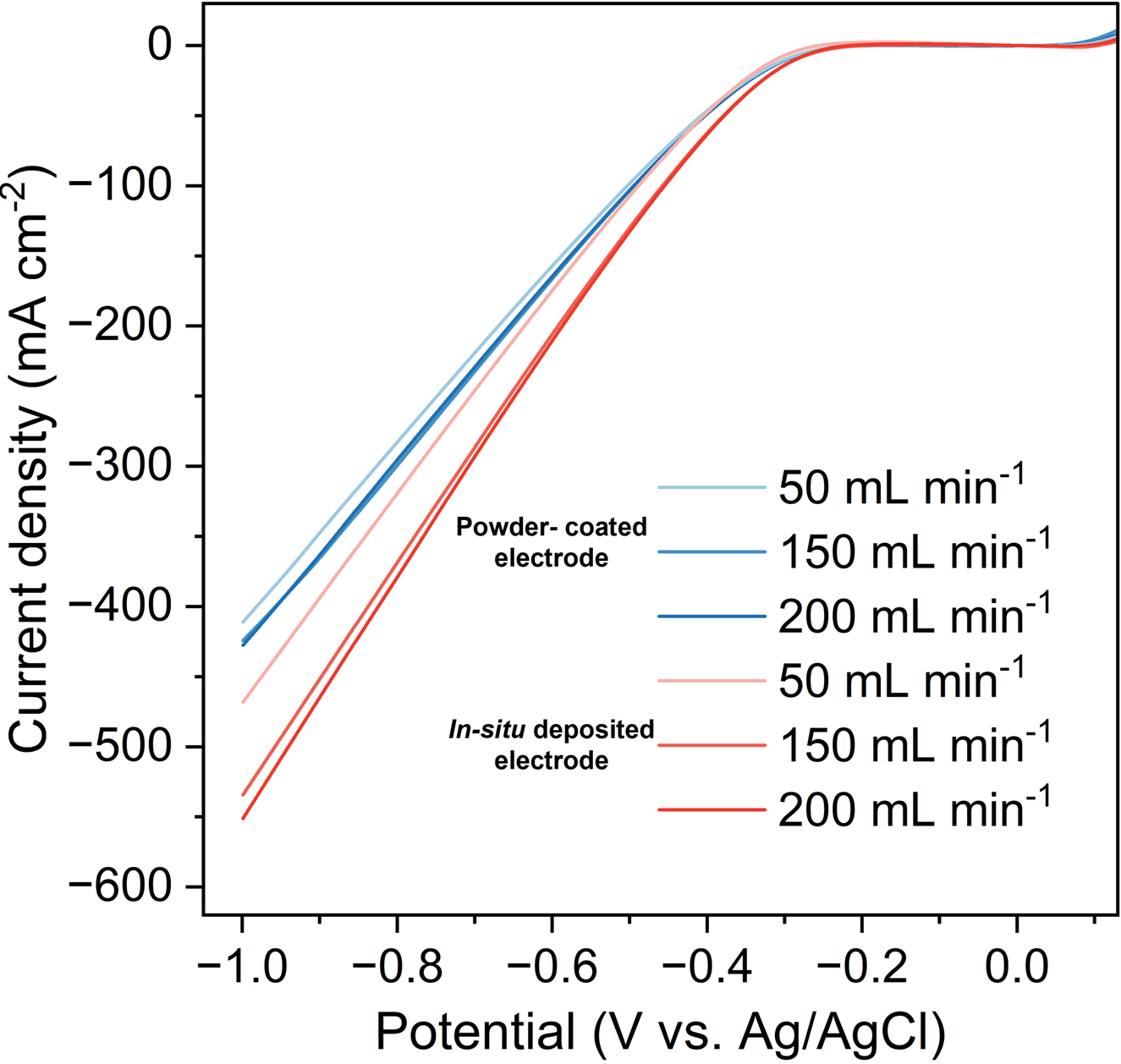


# **Figure S25. ORR polarization curves of the SA–Pd–CS powder coated electrode and the SA–Pd–CS in-situ deposited electrode under various O_2_ flow rate.** Catalyst loading: 0.5 mg cm^–2^, electrode area: 1 × 1 cm^2^, electrolyte: 0.1 M KOH.


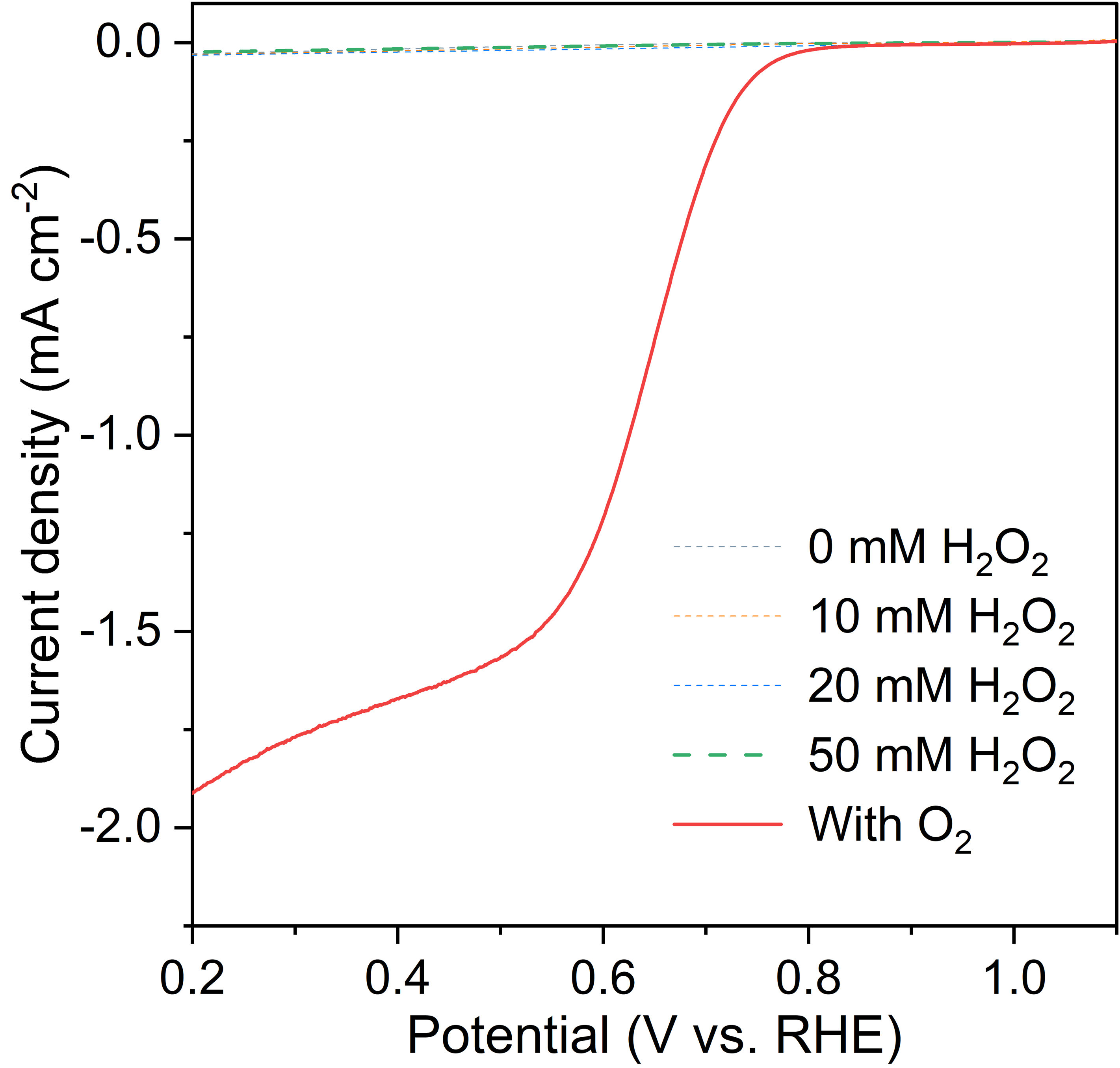


# **Figure S26. LSV for H_2_O_2_ reduction reaction under N_2_ and ORR under O_2_ in 0.1 M KOH.**


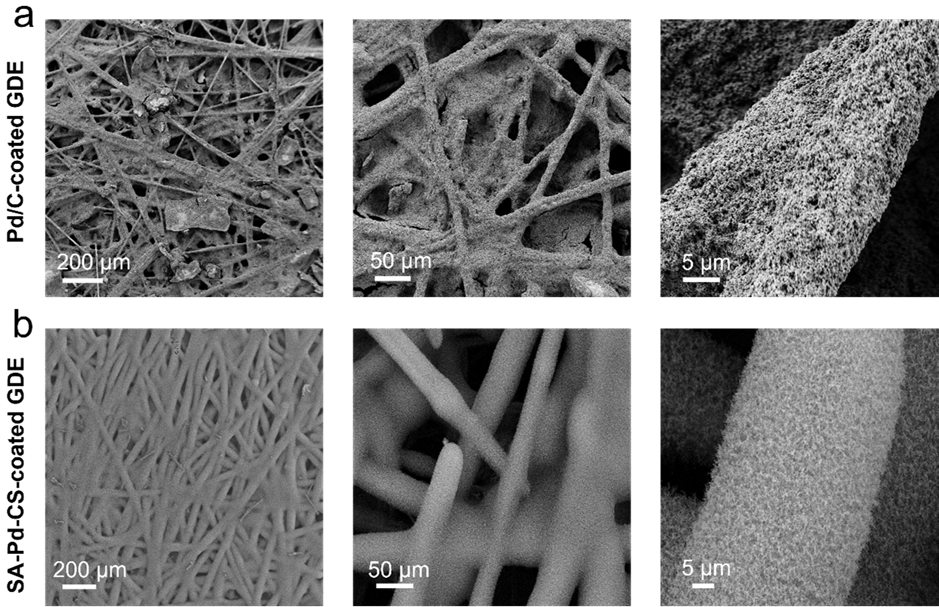


# **Figure S****27. SEM images of the catalysts-coated electrode.** a) Commercial powdery Pd/C catalyst-coated carbon paper electrode, b) SA-Pd-CS-coated carbon paper electrode.


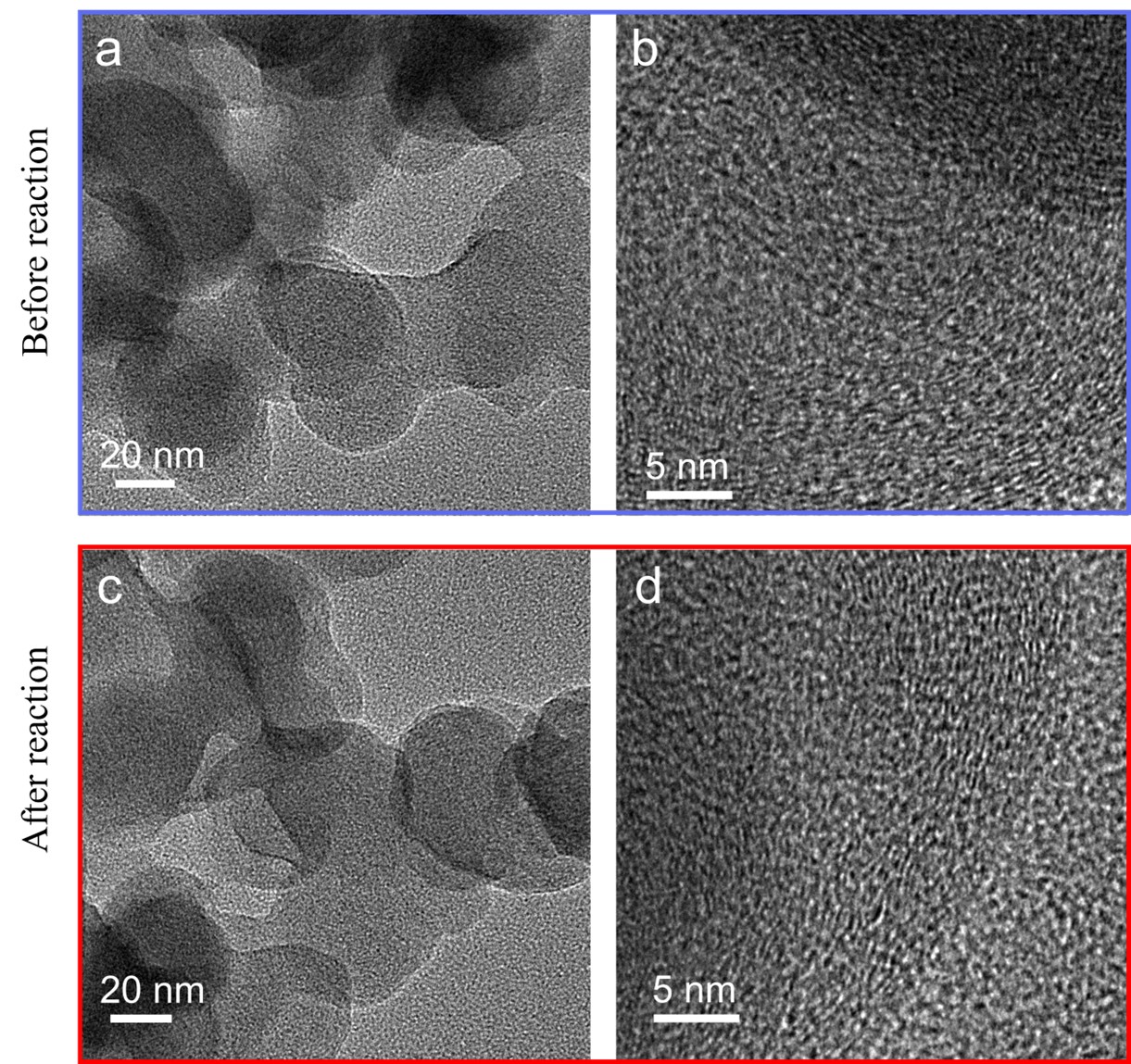


**Figure S28 TEM images of the SA-Pd-CS-coated electrode before and after reaction.** a,b) TEM images of SA-Pd-CS before reaction, c,d) TEM images of SA-Pd-CS after reaction.


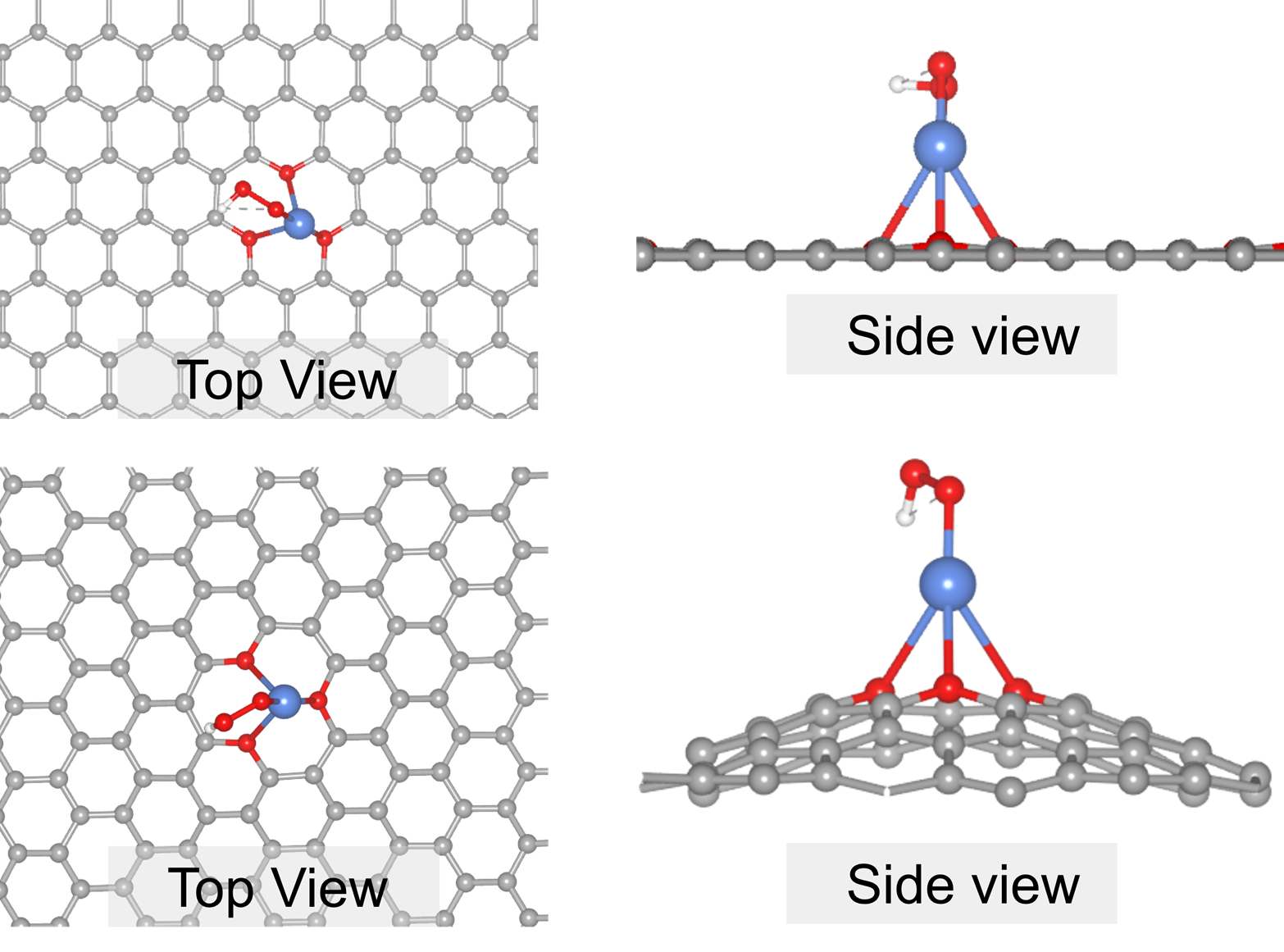


# **Figure S29. Configurations of the O_2_ intermediates on planar and bent Pd-O_3_ single sites.**

·


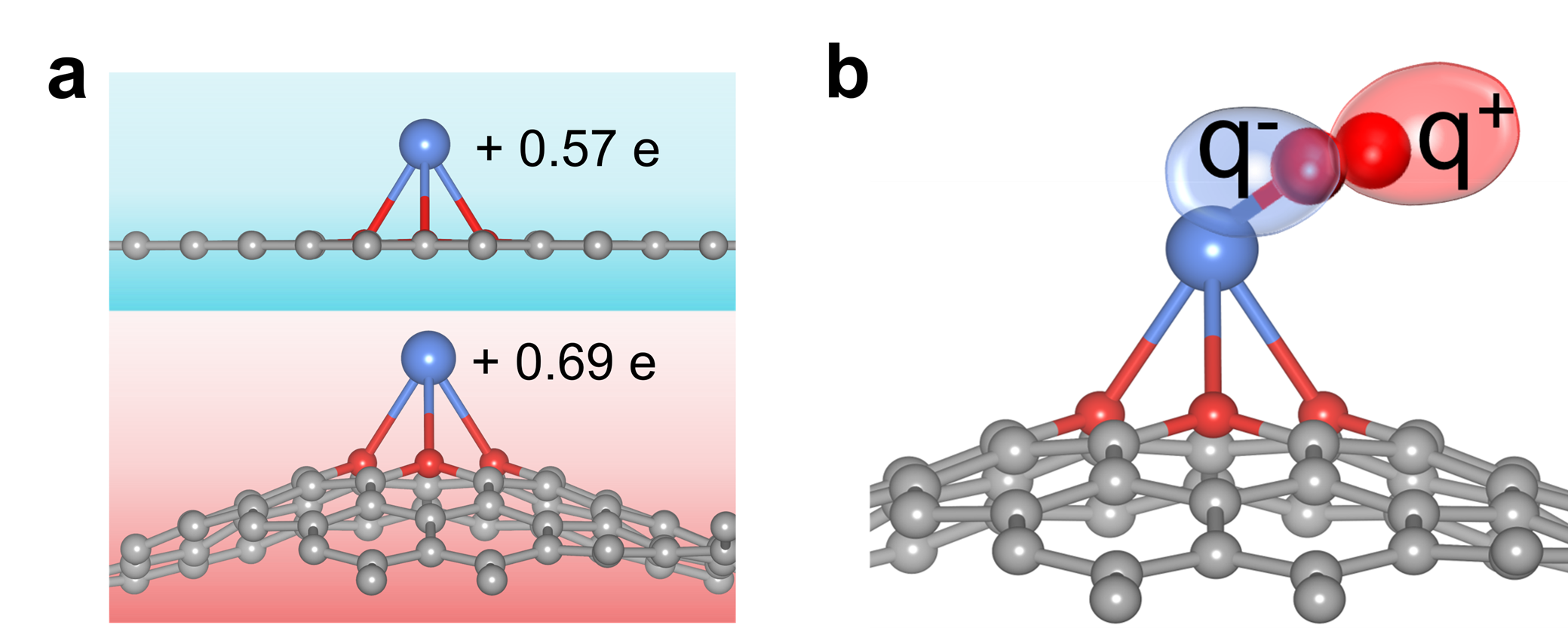


**Figure S30. Charge distribution of the O_2_-adsorbed single-atom Pd sites.** a) Barder charge on Pd for Pd-O_3_ sites anchored on bent and plane graphitic carbons. b) Schematic illustration of the O_2_ diploe moment on bent Pd-O_3_ sites.


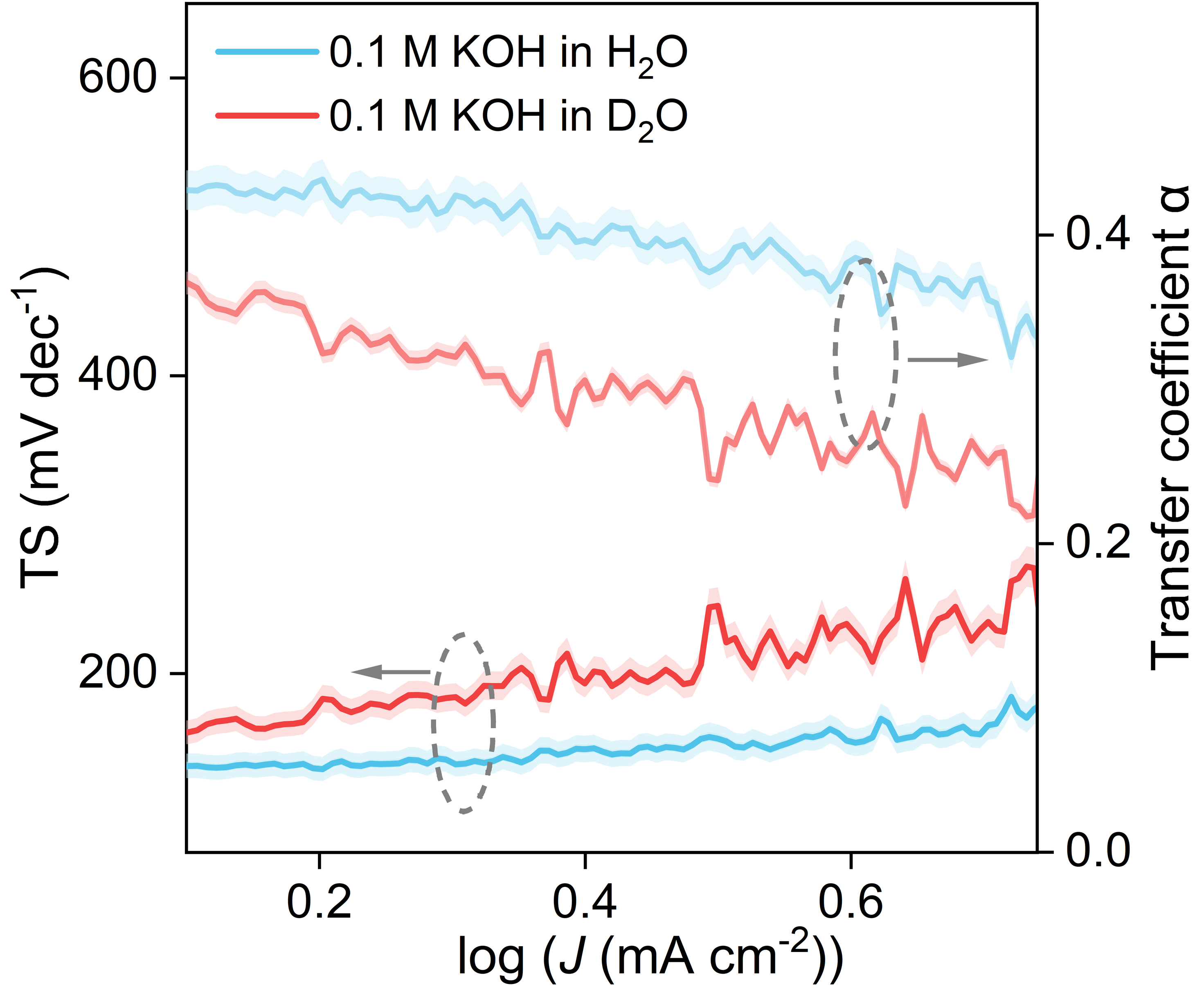


**Figure S31 TS and transfer coefficients *α* versus log(*J*) derived from the ORR polarization curves**

The Tafel slope (TS) is a fundamental parameter for characterizing reaction kinetics in electrocatalysis. Experimentally, it is obtained by linear fitting of the Tafel plot over an extensive range of log(J). Mathematically, it is defined by the simplified Butler-Volmer equation as the first derivative of the applied potential (E) with respect to the logarithm of the current density [log(J)]^11^:

In this equation, R, F, T, and α represent the ideal gas constant, Faraday constant, absolute temperature, and charge transfer coefficient, respectively.

For a multistep process featuring a single rate-determining step (RDS), the overall charge transfer coefficient (α) is given by α = n₁ + n₂β (2), where n₁ and n₂ are the number of electrons transferred before and during the RDS, respectively, and β is the symmetry factor of the elementary charge transfer step.


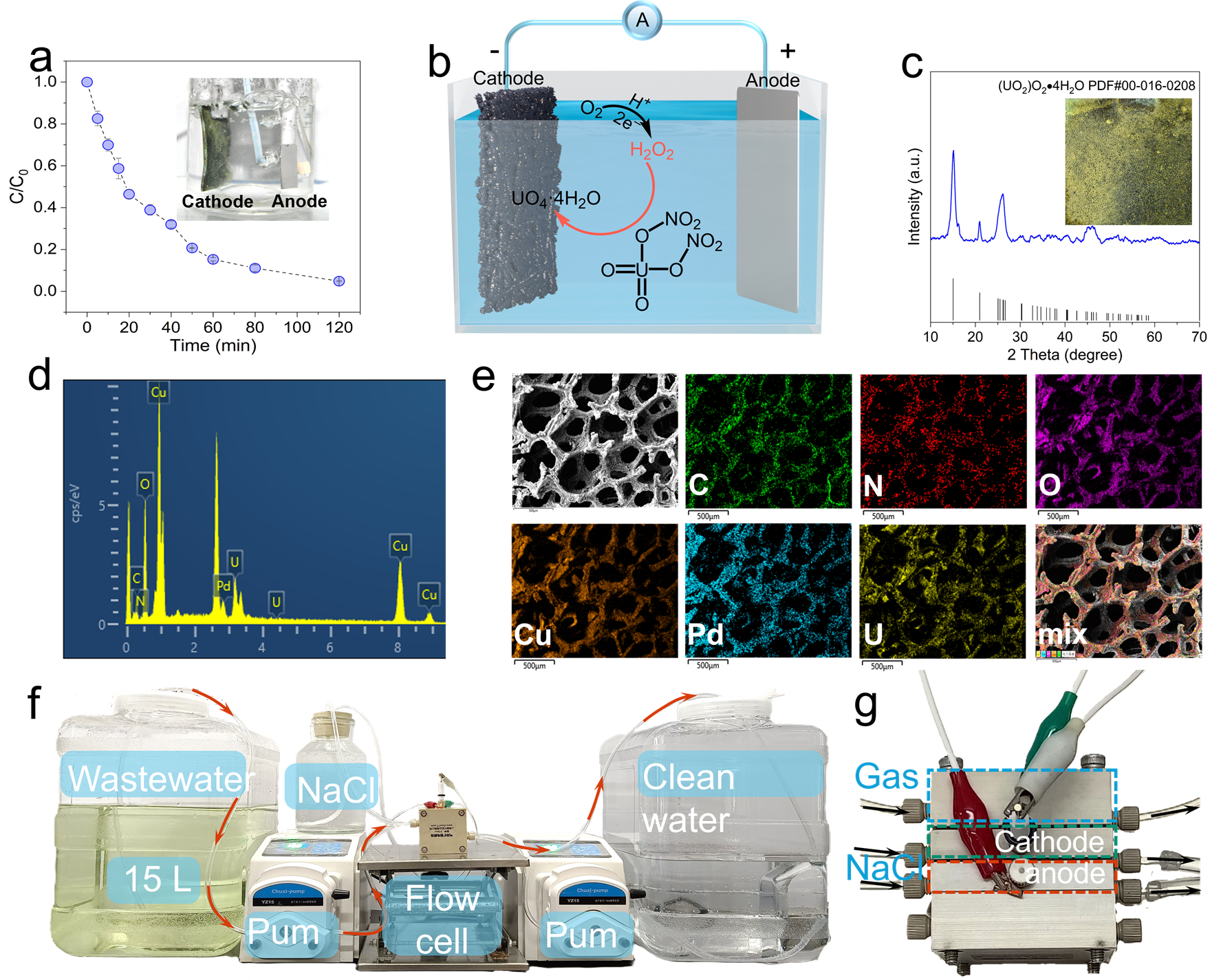


# **Figure S32.** **Treatment of U(VI)-containing wastewater using SA-Pd-CS cathodic catalyst.** a) U(VI) reduction and precipitation on the SA-Pd-CS cathode. b) Schematic illustration of the U(VI) precipitation on the cathodic catalyst. c) XRD pattern and digital picture of the yellow (UO_2_)O_2_🞄4H_2_O precipitated on the electrode. d) SEM-EDS spectra of the (UO_2_)O_2_🞄4H_2_O precipitated on the electrode, e) and the corresponding SEM-EDS mapping. f,g) Set-up for continuous flow treatment of U(VI) wastewater using the flow-cell device. Reaction conditions: Potential = ‒0.6 V, [NaCl] = 0.5 M, [UO_2_(NO_3_)_2_🞄6H_2_O] = 10 ppm, pH = 7.0

**Figures S32a** and **b** demonstrate that the SA-Pd-CS-coated cathode effectively eliminated U(VI) using in-situ-generated H_2_O_2_ in a single-cell system, achieving a high removal efficiency (95%) within 2 h. XRD and the SEM-EDS analyses (**Figures S32c-e**) confirmed that the U(VI) is precipitated on the electrode surface to form (UO_2_)O_2_🞄4H_2_O. **Figures S32f** and **g** show the setup for continuous-flow treatment of the U(VI) wastewater.


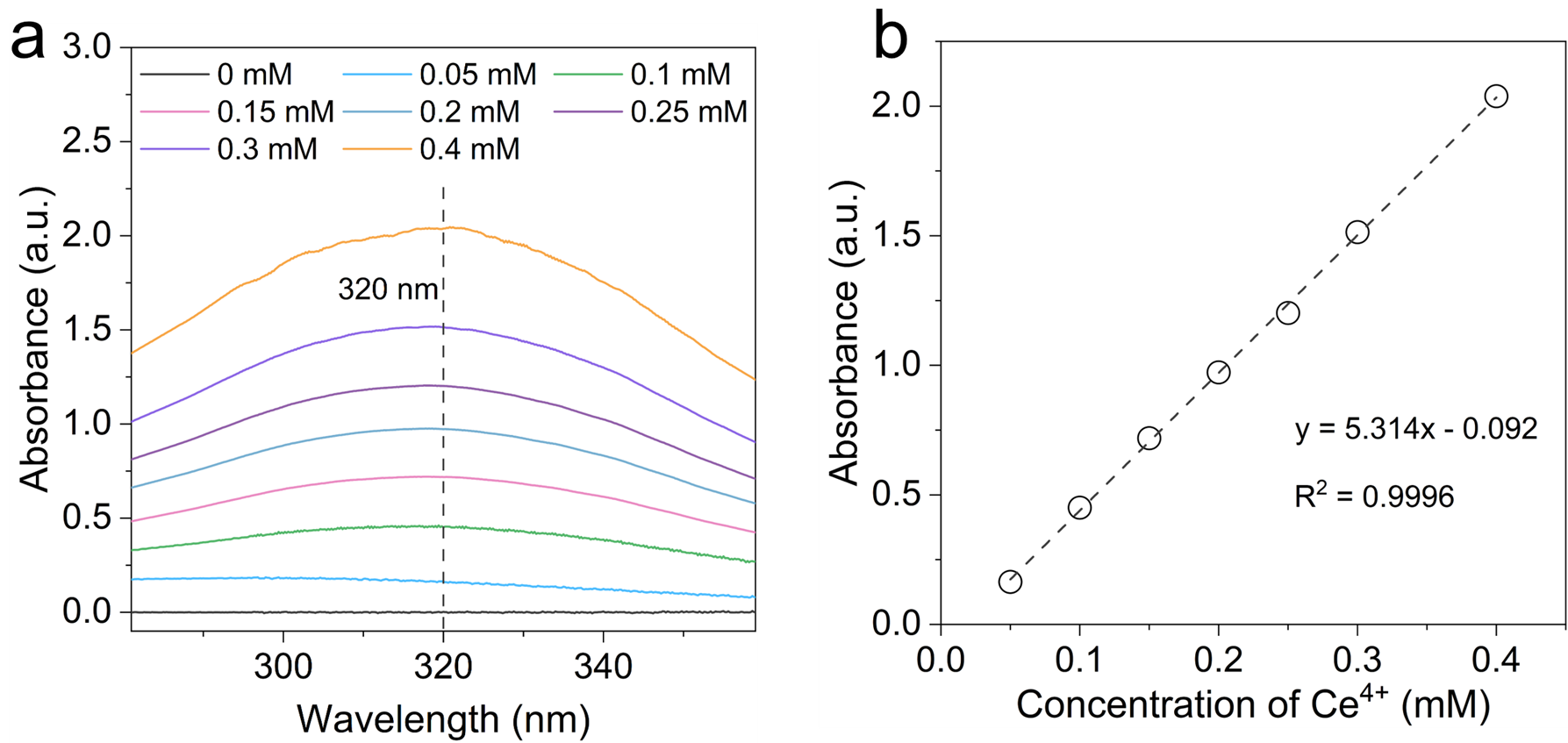


**Figure S****33. Calibration curve of Ce^4+^ for determination of H_2_O_2_ concentration.** a) UV-Vis absorption spectra of Ce^4+^. b) The corresponding calibration curve for Ce^4+^ concentrations versus absorbance at 320 nm.


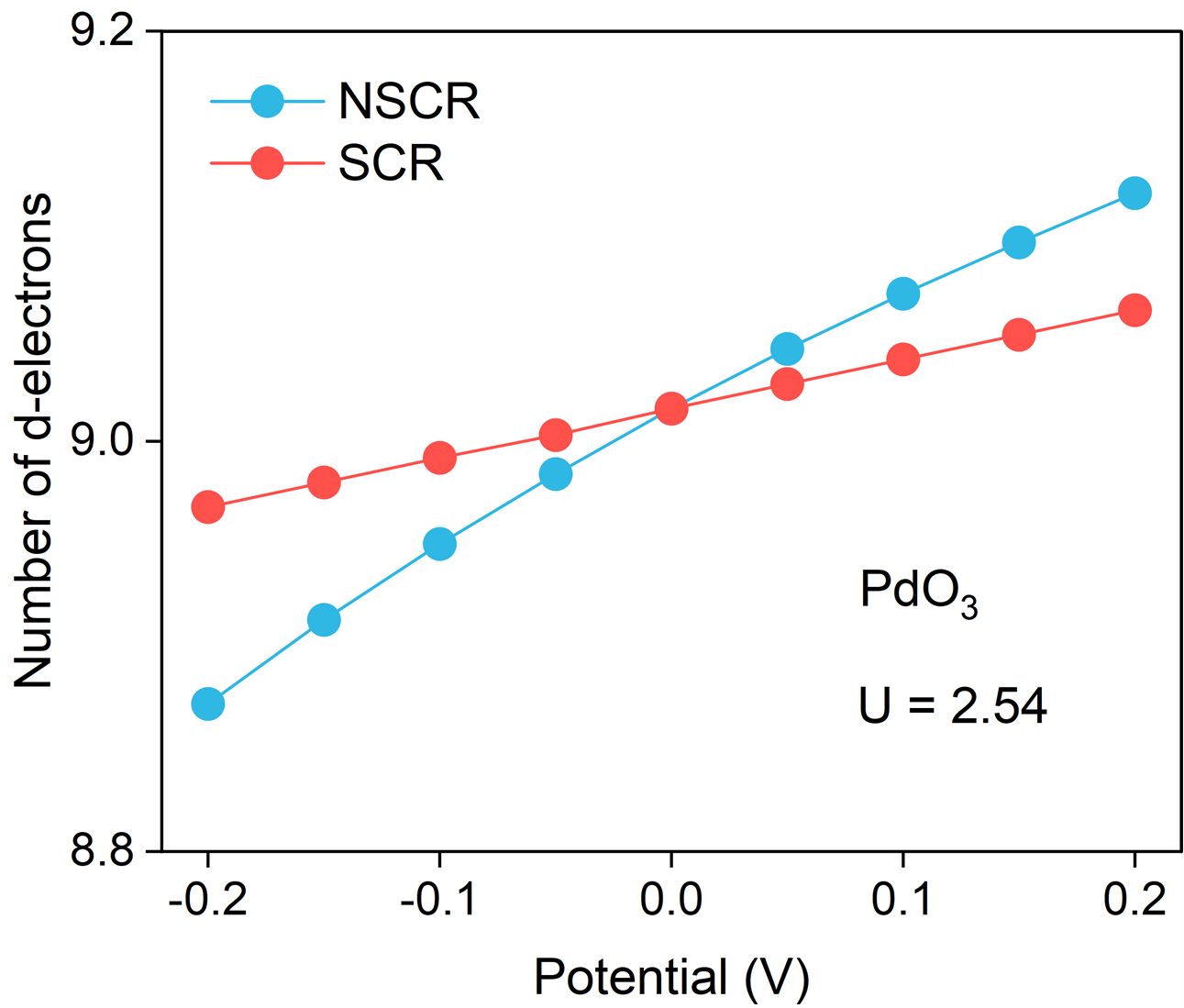


**Figure S34. Calculated Hubbard U parameter for Pd in PdO_3_ single-atom catalysts.**


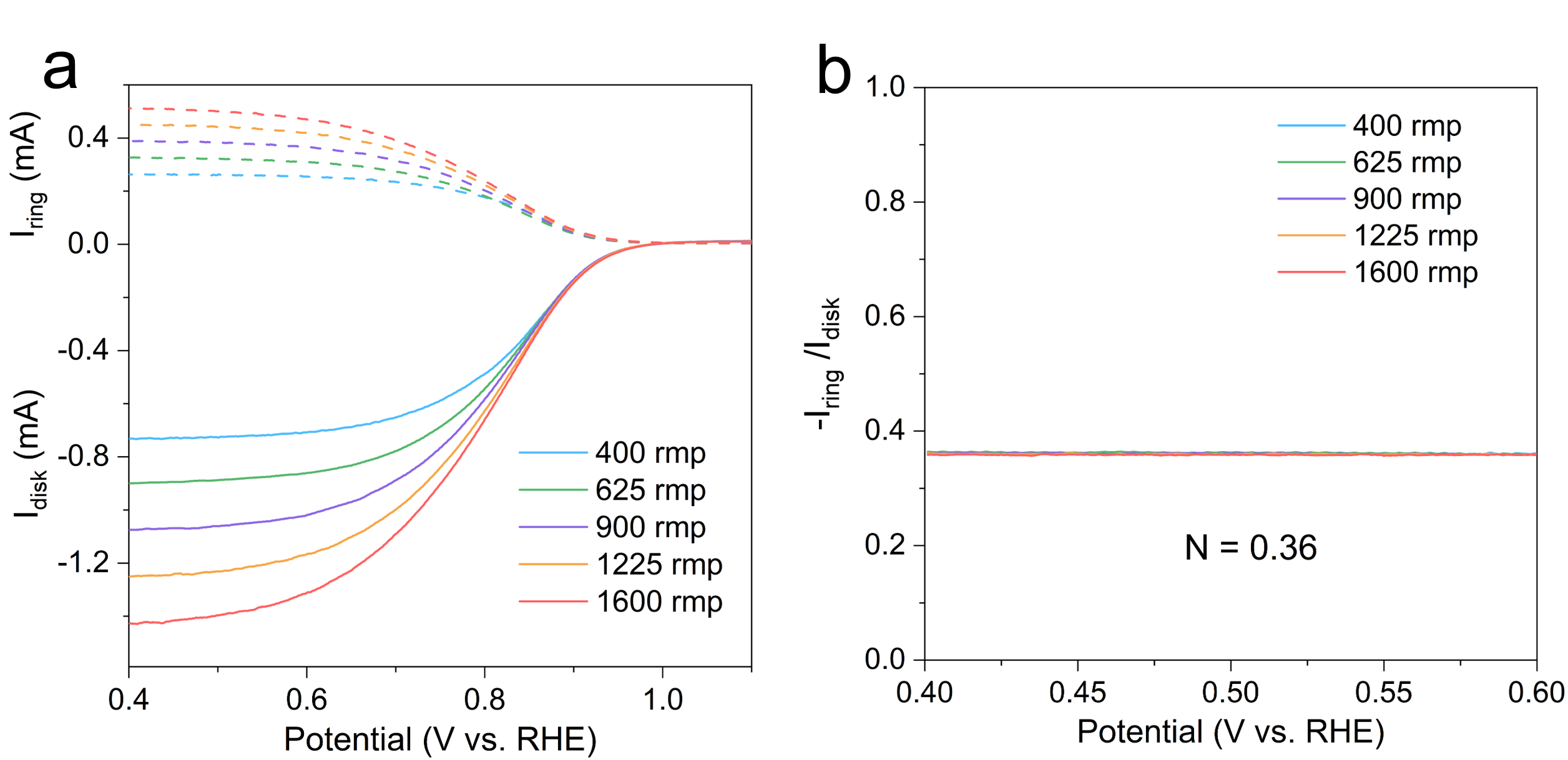


# **Figure S****35. Calculation of the collection efficiency**. a) LSV curves in 1 M KCl with 10 mM K_3_[Fe(CN)_6_] at different rotation speeds. b) Calculation of the collection efficiency (*N*).

# **Table S1 Metal loadings in SA-M-CS, determined by ICP-MS.**

| Sample | Loading (wt%) |
| --- | --- |
| SA-Zn-CS-1 | 0.29 |
| SA-Zn-CS-2 | 0.42 |
| SA-Zn-CS-3 | 0.53 |
| SA-Zn-CS-4 | 0.63 |
| SA-Zn-CS-5 | 0.67 |
| SA-Zn-CS-6 | 0.87 |
| SA-V-CS | 0.53 |
| SA-Cr-CS | 0.54 |
| SA-Mn-CS | 0.56 |
| SA-Fe-CS | 0.56 |
| SA-Co-CS | 0.57 |
| SA-Ni-CS | 0.54 |
| SA-Cu-CS | 0.56 |
| SA-Zn-CS | 0.53 |
| SA-Ru-CS | 0.60 |
| SA-Rh-CS | 0.62 |
| SA-Pd-CS | 0.57 |
| SA-In-CS | 0.60 |

# **Table S2 BET surface area and pore-size distribution of the various SA-M-CS samples**

| Sample | BET Surface Area (m^2^ g^-1^) | Pore Size  (nm) |
| --- | --- | --- |
| SA-V-CS | 65.38 | 6.96 |
| SA-Cr-CS | 59.06 | 12.25 |
| SA-Mn-CS | 54.16 | 6.32 |
| SA-Fe-CS | 66.09 | 7.40 |
| SA-Co-CS | 64.90 | 11.93 |
| SA-Ni-CS | 57.74 | 10.76 |
| SA-Cu-CS | 70.95 | 8.42 |
| SA-Zn-CS | 81.28 | 8.87 |
| SA-Ru-CS | 82.36 | 8.15 |
| SA-Rh-CS | 70.46 | 7.12 |
| SA-Pd-CS | 66.25 | 8.36 |
| SA-In-CS | 57.52 | 7.07 |

# **Table S3 EXAFS fitting parameters of the SA-M-CS SACs**

| Sample | Metal precursor | | Structure | Scattering Pair | CN | σ^2^  (Å^2^) | ΔE_0_  (ev) | R(Å) | R-factor |
| --- | --- | --- | --- | --- | --- | --- | --- | --- | --- |
| SA-V-CS*^a^* | 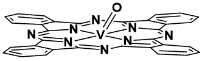 | VN_4_O | | V-O | 1.1 | 0.014±0.02 | -0.7±0.05 | 1.99 | 0.011 |
|  |  |  |  | V-N | 4.3 | 0.023±0.02 | 0.13±0.07 | 1.96 |  |
| SA-Cr-CS*^b^* | 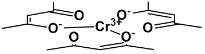 | CrO_4_ | | Cr-O | 4.0 | -0.003±0.002 | -0.07±0.01 | 1.96 | 0.013 |
| SA-Mn-CS*^c^* | 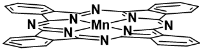 | MnN_4_ | | Mn-N | 4.0 | 0.006±0.003 | 0.7±0.07 | 2.14 | 0.010 |
| SA-Fe-CS*^d^* | 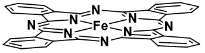 | FeN_4_ | | Fe-N | 4.0 | 0.013±0.01 | 0.25±0.04 | 2.09 | 0.006 |
| SA-Co-CS-1*^e^* | 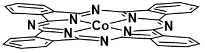 | CoN_4_ | | Co-N | 3.9 | 0.010±0.001 | 0.042±0.01 | 2.05 | 0.006 |
| SA-Co-CS-2*^f^* | 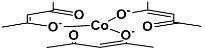 | CoO_4_ | | Co-O | 4.1 | 0.008±0.001 | -0.13±0.04 | 2.02 | 0.011 |
| SA-Co-CS-3*^g^* | 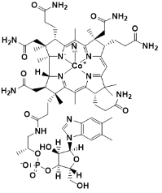 | CoN_5_ | | Co-N | 4.9 | 0.016±0.002 | 0.157±0.04 | 2.0 | 0.003 |
| SA-Ni-CS*^h^* | 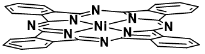 | NiN_4_ | | Ni-N | 3.8 | 0.006±0.003 | -0.2±0.02 | 2.0 | 0.015 |
| SA-Cu-CS*^i^* | 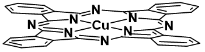 | CuN_4_ | | Cu-N | 4.1 | -0.002±0.0004 | -0.10±0.04 | 1.98 | 0.015 |
| SA-Zn-CS*^j^* | 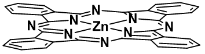 | ZnN_4_ | | Zn-N | 3.9 | 0.083±0.04 | -0.002±0.006 | 2.05 | 0.015 |
| SA-Ru-CS*^k^* | 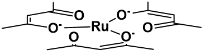 | RuO_3_ | | Ru-O | 3.0 | 0.002±0.001 | 0.09±0.02 | 2.06 | 0.012 |
| SA-Rh-CS*^l^* | 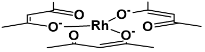 | RhO_3_ | | Rh-O | 3.1 | 0.002±0.001 | 0.06±0.02 | 2.04 | 0.016 |
| SA-Pd-CS*^m^* | 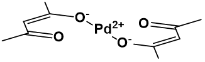 | PdO_3_ | | Pd-O | 3.0 | 0.004±0.001 | -0.072±0.03 | 2.00 | 0.014 |
| SA-In-CS*^n^* | 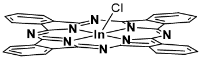 | InN_4_ | | In-N | 4.0 | 0.007±0.001 | -0.05±0.01 | 2.13 | 0.010 |

Note: S_0_^2^ is the amplitude reduction factor which was fixed to 0.85 during the fitting process, CN is the coordination number, R is interatomic distance (the bond length between central atoms and surrounding coordination atoms), σ^2^ is Debye-Waller factor (a measure of thermal and static disorder in absorber-scatterer distances), ΔE_0_ is edge-energy shift (the difference between the zero kinetic energy value of the sample and that of the theoretical model), R-factor is used to value the goodness of the fitting. The EXAFS fittings were performed using Artemis software.

# **Table S4 ECSA values of the SA-M-CS SACs.**

| Sample | ECSA (mF cm^-2^) | Rference |
| --- | --- | --- |
| SA-V-CS | 9.50 | This work |
| SA-Cr-CS | 2.04 |  |
| SA-Mn-CS | 7.00 |  |
| SA-Fe-CS | 4.37 |  |
| SA-Co-CS | 12.55 |  |
| SA-Ni-CS | 11.55 |  |
| SA-Cu-CS | 4.53 |  |
| SA-Zn-CS | 26.41 |  |
| SA-Ru-CS | 32.55 |  |
| SA-Rh-CS | 28.83 |  |
| SA-Pd-CS | 41.75 |  |
| SA-In-CS | 18.03 |  |
| cPI-CNF-Fe/Co | 20.2 | ^12^ |
| Pt@N-CTs-1.04 | 9.34 | ^13^ |
| Co-N-SAC_Mm_ | 7.3 | ^14^ |
| Co SACs/cBSC | 0.162 | ^15^ |
| Cu SA/NC | 26.4 | ^16^ |
| Fe_SA_-S/N-C | 17.2 | ^17^ |
| α-MoC/N-C/RuNSA | 33.4 | ^18^ |
| Ru-Co_3_O_4_ (4.49 wt%) | 26.42 | ^19^ |
| NiCd-HDAC | 1.36 | ^20^ |
| Ni_SA_-O/Mo_2_C | 0.22 | ^21^ |
| A-Fe_2_S_1_N_5_/SNC | 3.2 | ^22^ |
| CuPc | 0.028 | ^23^ |
| NGA-COF@Pt | 21.00 | ^24^ |
| NiSA/PCFM | 20.2 | ^25^ |

# **Table S5 Comparisons of the** **Faradic Efficiency for H_2_O_2_ production in the electrochemical cells using SA-Pd-CS-coated GDE and the recently reported state-of-the-art catalysts-coated electrodes.**

| Catalyst | Current density (mA cm^-2^) | Cell Type | Faradic Efficiency (%) | Ref. |
| --- | --- | --- | --- | --- |
| **SA-Pd-CS** | **50** | **Flow Cell**  **(0.1 M KOH)** | **99.3** | **This work** |
|  | **100** |  | **98.5** |  |
|  | **200** |  | **97.9** |  |
|  | **300** |  | **95.5** |  |
|  | **400** |  | **92.6** |  |
|  | **500** |  | **90.8** |  |
|  | **50** | **Flow Cell**  **(0.5 M K_2_SO_4_)** | **94.3** |  |
|  | **100** |  | **94.1** |  |
|  | **200** |  | **95.0** |  |
|  | **300** |  | **93.3** |  |
|  | **400** |  | **90.7** |  |
|  | **500** |  | **89.8** |  |
|  | **50** | **Flow Cell**  **(0.1 M HClO_4_)** | **97.0** |  |
|  | **100** |  | **93.0** |  |
|  | **200** |  | **93.3** |  |
|  | **300** |  | **90.9** |  |
|  | **400** |  | **88.0** |  |
|  | **500** |  | **87.9** |  |
| Sn_1_/C(O) | 100 | Flow Cell  (1 M KOH) | 98.9 | ^26^ |
|  | 200 |  | 98.3 |  |
|  | 300 |  | 96.1 |  |
|  | 400 |  | 94.2 |  |
| CoPc-OCNT | 100 | Flow Cell  (1 M KOH) | 93 | ^27^ |
|  | 200 |  | 99 |  |
|  | 300 |  | 98 |  |
| CoN_4_/VG | 10 | Flow Cell  (0.1 M HClO_4_) | 95 | ^28^ |
|  | 17 |  | 87 |  |
|  | 24 |  | 73 |  |
|  | 210 |  | 70 |  |
| CoPc-CNT(O) | 50 | Flow Cell  (1 M Na_2_SO_4_) | 95 | ^29^ |
|  | 300 |  | 91 |  |
|  | 450 |  | 89 |  |
| Co HSACs | 300 | Flow Cell  (0.5 M KOH) | 90 | ^23^ |
| CNB-ZIL | 40 | Flow Cell  (0.1 M KOH) | 90 | ^30^ |
| CoIn-N-C | 70 | Flow Cell  (1 M HClO_4_) | 85 | ^31^ |
|  | 100 |  | 84 |  |
|  | 125 |  | 46 |  |
| NiZnO_x_-C | 20 | Flow Cell  (1 M KOH) | ~29.5 | ^32^ |
|  | 50 |  | ~32 |  |
|  | 100 |  | ~32.1 |  |
|  | 150 |  | ~38.2 |  |
|  | 200 |  | 55.9 |  |
|  | 250 |  | ~34.2 |  |
|  | 300 |  | ~24.7 |  |
| CoPorF/CNT | 185 | Solid-state-electrolyte cell O_2_//SE/H_2_O | 87 | ^33^ |
|  | 260 |  | 80 |  |
|  | 340 |  | 75 |  |
|  | 400 |  | 80 |  |
| CB | 85 | Solid-state-electrolyte cell O_2_//SE/H_2_O | 95 | ^34^ |
|  | 110 |  | 94 |  |
|  | 140 |  | 93 |  |
|  | 170 |  | 91 |  |
|  | 200 |  | 88 |  |
| B-C | 100 | Solid-state-electrolyte cell O_2_//SE/H_2_O | 92 | ^35^ |
|  | 200 |  | 90 |  |
|  | 300 |  | 90 |  |
|  | 400 |  | 87 |  |
|  | 500 |  | 79 |  |
| N,S-TCNTs | 150 | Solid-state-electrolyte cell Air//SE/H_2_O | 95 | ^36^ |
|  | 200 |  | 90 |  |
|  | 250 |  | 87 |  |
|  | 300 |  | 78 |  |
| FTO | 100 | Fuel Cell  (5 M K_2_CO_3_) | ~83.5 | ^37^ |
|  | 150 |  | 87 |  |
|  | 200 |  | ~80.5 |  |
|  | 400 |  | ~83.9 |  |
|  | 600 |  | 87 |  |
| PtP_2_ NCs | 25 | Fuel Cell  (0.1 M HClO_4_) | 65.3 | ^38^ |
|  | 50 |  | 68.6 |  |
|  | 75 |  | 71.3 |  |
|  | 100 |  | 73.8 |  |
|  | 125 |  | 64.8 |  |
|  | 150 |  | 78.8 |  |
|  | 175 |  | 66.5 |  |
|  | 200 |  | 52.7 |  |
| utn-NiBDC | 25 | Flow Cell  (0.1 M KOH) | 82 | ^39^ |
|  | 50 |  | 92.7 |  |
|  | 75 |  | 88.7 |  |
|  | 100 |  | 84.4 |  |
|  | 150 |  | 84.1 |  |
|  | 200 |  | 91.9 |  |
| Co_2_ DAC | 50 | Flow Cell  (0.1 M HClO_4_) | 98.9 | ^40^ |
|  | 100 |  | 99.3 |  |
|  | 150 |  | 90.2 |  |
|  | 200 |  | 87.1 |  |
|  | 250 |  | 85.0 |  |
|  | 300 |  | 79.6 |  |
|  | 350 |  | 79.3 |  |
|  | 400 |  | 78.4 |  |
|  | 450 |  | 59.6 |  |
|  | 500 |  | 41.2 |  |

# **Table S6 Comparisons of the H_2_O_2_ production rates in the electrochemical cell using SA-Pd-CS-coated GDE and the recently reported single-atom catalysts-coated electrodes (Single-atom catalysts).**

| Catalyst | Current density (mA cm^-2^) | Cell Type | Production rate  (mol g_cat_^−1^ h^−1^) | Ref. |
| --- | --- | --- | --- | --- |
| SA−Pd−CS | 500 | Flow cell  (0.1 M KOH) | 15.9 | This work |
| Co-N SAC_Dp_ | 50 | Flow cell  (0.1 M HClO_4_) | 0.66 | ^14^ |
| CoN_4_/VG | 21 | Flow cell  (0.1 M HClO_4_) | 0.31 | ^28^ |
| Co_2_−DAC | 400 | Flow cell  (0.1 M HClO_4_) | 11.72 | ^40^ |
| CoPorF/CNT | 50 | Flow cell  (0.1 M HClO_4_) | 10.76 | ^33^ |
| Pt_1_/CoSe_2_ | ~8 | Flow cell  (0.1 M HClO_4_) | 0.11 | ^41^ |
| Au/F−GDY | ~150 | Flow cell  (0.1 M KOH) | ~13 | ^42^ |
| CoPc−OCNT | 300 | Flow cell  (1 M KOH) | 11.53 | ^27^ |
| Co-O_4_@PC | 0.29 | Flow cell  (0.1 M KOH) | 0.25 | ^43^ |
| Co_1_−NBC | 350 | Flow cell  (0.5 M H_2_SO_4_) | 5.96 | ^44^ |
| CoTPP@RGO−160 | 200 | Flow cell  (0.5 M H_2_SO_4_) | 3.15 | ^45^ |
| Pt−N−CNT_Gly | 300 | Flow cell  (0.05 M Na_2_SO_4_ + 0.05 M H_2_SO_4_) | 14.7 | ^46^ |
| Mn CD/C | 200 | Flow cell  (1 M KOH) | 8.68 | ^47^ |
| CB@Co-N-C | 50 | Flow cell  (0.5 M H_2_SO_4_) | 0.51 | ^48^ |
| Co-N-C | 50 | Flow cell  (0.1 M KOH) | 4.33 | ^49^ |
| In SAs/NSBC | 90 | PEMFC  (0.5 M Na_2_SO_4_) | 6.71 | ^50^ |
| Co SA/CC | 25 | Flow cell  (0.5 M H_2_SO_4_) | 0.68 | ^51^ |
| Ni-N_2_O_2_/C | 70 | Flow cell  (0.1 M KOH) | 5.9 | ^52^ |
| Fe-CNT | 20 | H cell  (0.1 M PBS) | 0.136 | ^53^ |
| Fe_SA_-NS/C-700 | 100 | Flow cell  (0.1 M HCl) | 4.95 | ^54^ |
| Ni/C-4 | 10 | Flow cell  (0.1 M KOH) | 0.91 | ^55^ |
| CSH-600 | 80 | Flow cell  (1 M KOH) | 6.92 | ^56^ |
| Sb-NSCF | ~50 | Flow cell  (1 M KOH) | 7.46 | ^57^ |
| Mn−NO−CH | 115 | Flow cell  (1 M KOH) | 15.1 | ^58^ |
| FeSAs/ACs-BCC | 150 | Flow cell  (1 M KOH) | 12.5 | ^59^ |
| Pt_0.21_/CN | ~4 | H cell  (0.1 M KOH) | 0.77 | ^60^ |
| CoN_4_-PC | 80 | Flow cell  (1 M KOH) | 11.2 | ^61^ |
| Sn_1_/C(O) | 400 | Flow cell  (1 M KOH) | 7 | ^62^ |
| Co-N-C | 50 | Flow cell  (0.5 M NaCl) | 4.5 | ^63^ |
| CoTPP/KB | 130 | PEMFC  (Solid-state-electrolyte cell O_2_//SE/H_2_O) | 0.475 | ^64^ |
| MnCl-OEP/AC  (823 K) | 56.1 | PEMFC  (0.6 M H_2_SO_4_) | 0.018 | ^65^ |
| N_4_-Ni_1_-O_2_ | 350 | Flow cell  (1 M KOH) | 6 | ^66^ |
| Co HSACs | 300 | Flow cell  (0.5 M KOH) | 10 | ^23^ |

# **Table S7 Comparisons of the H_2_O_2_ production rates in the electrochemical cell using SA-Pd-CS-coated GDE and the recently reported Metal compounds or particles-coated electrodes (Metal compounds/particles).**

| Catalyst | Current density (mA cm^-2^) | Cell Type | Production rate  (mol g_cat_^−1^ h^−1^) | Ref. |
| --- | --- | --- | --- | --- |
| D-PSFZ | ~70 | H cell  (1 M KHCO_3_) | 0.52 | ^67^ |
| ZnCo-ZIF-C3 | 80 | Flow cell  (0.1 M KOH) | 4.35 | ^68^ |
| Pd^δ+^-OCNT | ~10 | H cell  (0.1 M HClO_4_) | 1.70 | ^69^ |
| Fe-CeO_2_-3 | ~2 | H cell  (0.1 M KOH) | 1.80 | ^70^ |
| Pd/MCS-8 | ~250 | Flow cell  (0.5 M K_2_SO_4_) | 15.77 | ^71^ |
| NC-Ag/NHCS | ~3 | 0.1 M HClO_4_ | 0.41 | ^72^ |
| CuNW@8CoSx | ~5.4 | H cell  (0.1 M Na_2_SO_4_) | 0.24 | ^73^ |
| Pd NCs/NiTe_2_ | 100 | Flow cell  (0.1 M KOH) | 1.43 | ^74^ |
| CuZn-MOF(H) | 25 | Flow cell  (0.1 M KOH) | ~4.5 | ^75^ |
| Bi/PNC-4 | ~35 | Flow cell  (0.1 M KOH) | 5.29 | ^76^ |
| a-PdSe_2_ NPs | 50 | Flow cell  (0.1 M Na_2_SO_4_) | 0.361 | ^77^ |
| Co_NPs_@N/C | 1 | Single cell  (0.1 M KOH) | 3.8 | ^78^ |
| Ag-TCNQ/rGO | 20 | Flow cell  (0.1 M KOH) | 5.57 | ^79^ |
| Ca-COF-318 | 12.71 | H cell  (0.1 M KOH) | 2.27 | ^80^ |
| Br–Ni MOF | 25 | Flow cell  (0.1 M KOH) | 1.534 | ^81^ |
| h-Pt_1_-CuS_x_ | 10 | H cell  (0.5 M HClO_4_) | 0.546 | ^82^ |
| 6h-Pd/TiC | 22 | H cell  (1 M KOH) | 3.06 | ^83^ |
| Co-C | 30 | PEMFC  (Solid-state-electrolyte cell O_2_//SE/H_2_O) | 0.056 | ^84^ |

| Catalyst | Current density  (mA cm^-2^) | Cell Type | Production rate  (mol g_cat_^−1^ h^−1^) | Ref. |
| --- | --- | --- | --- | --- |

| CoTPP/VGCF  (1073 K) | 80 | PEMFC  (Solid-state-electrolyte cell O_2_//SE/H_2_O) | 0.1458 | ^85^ |
| --- | --- | --- | --- | --- |
| PtP_2_ | 150 | PEMFC  (Solid-state-electrolyte cell O_2_//SE/H_2_O) | 2.83 | ^38^ |
| h-SnO2 | 100 | Flow cell  (pure water) | 3.89 | ^86^ |
| Ov-Bi_2_O_3_-EO | ~125 | Flow cell  (0.1 M KOH + 2 M KHCO_3_) | 2.330 | ^87^ |
| ZnO@ZnO_2_ | ~120 | Flow cell  (0.1 M K_2_SO_4_) | 5.47 | ^88^ |
| Co_3_O_4_-350 | 80 | H cell  (0.5 M H_2_SO_4_) | 1.6 | ^89^ |
| Cu-800@C | ~33 | Flow cell  (0.1 M KOH) | 0.921 | ^90^ |
| NiB_2_ | 125 | Flow cell  (0.1 M KOH) | 4.753 | ^91^ |
| AgNbO_3_ | 250 | Flow cell  (1 M KOH) | 4.15 | ^92^ |
| α-Fe_2_O_3_ | 55 | H cell  (1 M KOH) | 0.454 | ^93^ |
| AD-Pt@AuCu-144 | 2 | H cell  (0.1 M HClO_4_) | 0.186 | ^94^ |
| *o*-CoSe_2_/CFP | ~0.7 | H cell  (0.05 M H_2_SO_4_) | 0.004 | ^95^ |
| Ni_2_Mo_6_S_8_ | 1.5 | H cell  (0.1 M KOH) | 0.09 | ^96^ |
| NiO*_x_*-C | 2.5 | H cell  (0.1 M KOH) | 0.145 | ^97^ |
| CuCo_0.8_Ni_1.2_S_4_ | ~2 | H cell  (0.05 M H_2_SO_4_) | 0.197 | ^98^ |
| BP/CoSe_2_ | 54 | Flow cell  (0.5 M H_2_SO_4_) | 0.9 | ^99^ |
| CoPc-S-COF | 125 | Flow cell  (1 M KOH) | 9.5 | ^100^ |

# **Table S8 Comparisons of the H_2_O_2_ production rates in the electrochemical cell using SA-Pd-CS-coated GDE and the recently reported metal-free catalysts-coated electrodes (Metal-free catalysts).**

| Catalyst | Current density (mA cm^-2^) | Cell Type | Production rate  (mol g_cat_^−1^ h^−1^) | Ref. |
| --- | --- | --- | --- | --- |
| CMK-3 | ~3 | H cell  (1 M KOH) | ~0.562 | ^101^ |
| HPC | ~2 | H cell  (0.5 M H_2_SO_4_) | 0.17 | ^102^ |
| Meso-BMP-800 | 10 | Single cell  (0.1 M KOH) | 0.122 | ^103^ |
| oxo-G | 0.4 | H cell  (0.1 M KOH) | 0.225 | ^104^ |
| OCG-800 | 60 | H cell  (0.1 M KOH) | 0.474 | ^105^ |
| O-CNTs | 20 | H cell  (0.1 M KOH) | 0.112 | ^106^ |
| N-FLG-8 | ~110 | Flow cell  (0.1 M KOH) | 9.66 | ^107^ |
| CB-10% | 300 | SEC  (Solid-state-electrolyte cell O_2_//SE/H_2_O) | 3.66 | ^34^ |
| TP-TD-COF | ~2 | H cell  (0.1 M KOH) | 0.158 | ^108^ |
| BUCT-COF-7 | 10 | H cell  (0.1 M KOH) | 0.367 | ^109^ |
| N-O-P-C-800 | ~10 | Flow cell  (0.1 M KOH) | 1.47 | ^110^ |
| FPC-800 | ~2 | H cell  (0.1 M Na_2_SO_4_ + 0.05 M H_2_SO_4_) | 0.793 | ^111^ |
| N,S-TCNTs | 150 | SEC  (Solid-state-electrolyte cell Air//SE/H_2_O) | 14.31 | ^36^ |
| NADE | 240 | Flow cell  (0.05 M Na_2_SO_4_) | 0.227 | ^112^ |
| AC+VGCF | 30 | SEC  (Solid-state-electrolyte cell Air//SE/H_2_O) | 0.036 | ^113^ |
| VGCF+XC72 | 100 | PEMFC  (Solid-state-electrolyte cell Air//SE/H_2_O) | 0.0325 | ^114^ |
| NPC950 | 100 | Flow cell  (1 M KOH) | 8.53 | ^115^ |

**Table S9 Fitting parameters for the Nyquist plots by the equivalent circuit and Z-view software.**

| Samples | CPE (×10^-5^ Ω^-1^ s^n^ cm^-2^) | Rs (Ω cm^2^) | Rct (Ω cm^2^) | W (×10^-3^ Ω^-1^ s^0.5^ cm^-2^) |
| --- | --- | --- | --- | --- |
| SA-V-CS | 7.2 | 2.4 | 149.6 | 5.0 |
| SA-Cr-CS | 8.5 | 2.1 | 174.9 | 4.7 |
| SA-Mn-CS | 7.8 | 2.5 | 151.6 | 5.2 |
| SA-Fe-CS | 8.1 | 2.0 | 178.2 | 4.7 |
| SA-Co-CS | 9.7 | 2.0 | 168.8 | 5.1 |
| SA-Ni-CS | 7.5 | 2.1 | 156 | 4.5 |
| SA-Cu-CS | 9.0 | 2.8 | 170.2 | 4.8 |
| SA-Zn-CS | 8.7 | 3.0 | 135.5 | 5.4 |
| SA-Ru-CS | 8.8 | 2.7 | 137.2 | 5.6 |
| SA-Rh-CS | 10.5 | 2.4 | 168.6 | 6.0 |
| SA-Pd-CS | 8.9 | 2.5 | 118.9 | 5.5 |
| SA-In-CS | 7.6 | 2.6 | 156.6 | 4.8 |
| Pt/C | 6.7 | 2.5 | 325.4 | 3.5 |
| Pd/C | 6.3 | 2.1 | 305.4 | 3.7 |
| RuO_2_ | 7.5 | 2.1 | 288.5 | 3.9 |

**References**

1. Ravel, B. & Newville, M. J. J. o. s. r. ATHENA, ARTEMIS, HEPHAESTUS: data analysis for X-ray absorption spectroscopy using IFEFFIT. **12**, 537-541 (2005).
2. Han, S. et al. Ultralow overpotential nitrate reduction to ammonia via a three-step relay mechanism. *Nat. Catal.* **6**, 402−414 (2023).
3. Li, X. et al. Lithium ferrocyanide catholyte for high-energy and low-cost aqueous redox flow batteries. *Angew. Chem. Inter. Ed.* **62**, e202304667 (2023).
4. Yu, Z. et al. Selective and durable H_2_O_2_ electrosynthesis catalyst in acid by selenization induced straining and phasing. *Nat. Commun.* **15**, 9346 (2024).
5. Chen, W. et al. Challenges in unravelling the intrinsic kinetics of gas reactions at rotating disk electrodes by koutecky-levich equation. *J. Phys. Chem. C* **127**, 16235-16248 (2023).
6. Fang, Y.-H. & Liu, Z.-P. Tafel Kinetics of Electrocatalytic Reactions: From Experiment to First-Principles. *ACS Catal.* **4**, 4364-4376 (2014).
7. Quevedo, M. C. *et al*. Role of turbulent flow seawater in the corrosion enhancement of an Al-Zn-Mg alloy: an electrochemical impedance spectroscopy (EIS) analysis of oxygen reduction reaction (ORR). *J. Mater. Res. Technol.* **7**, 149-157(2018).
8. Zhou, W. *et al.* In situ tuning of platinum 5d valence states for four-electron oxygen reduction. *Nat. Commun.* **15**, 6650 (2024).
9. Wang, Z., Hu, N., Wang, L., Zhao, H. & Zhao, G. In situ production of hydroxyl radicals via three-electron oxygen reduction: opportunities for water treatment. *Angew. Chem. Int. Ed.* **63**, e202407628 (2024).
10. Zhang, X. *et al.* Electrochemical oxygen reduction to hydrogen peroxide at practical rates in strong acidic media. *Nat. Commun.* **13**, 2880 (2022).
11. Zhu, J. *et al.* Hybrid nanoalloy-cluster-single atom sites based aerophilic carbon fiber membranes as binder-free cathodes for ultra-long-life Zn-air batteries. *Adv. Funct. Mater.* 2416422 (2024).
12. Huang, J. *et al.* Isotope-dependent Tafel analysis probes proton transfer kinetics during electrocatalytic water splitting. *Nat. Chem.,* 1-8 (2025).
13. Pan, Q. *et al.* Pt single-atoms on structurally-integrated 3D N-doped carbon tubes grid for ampere-level current density hydrogen evolution. *Small* **20**, 2309067 (2024).
14. Chen, S. *et al.* Identification of the highly active Co-N_4_ coordination motif for selective oxygen reduction to hydrogen peroxide. *J. Am. Chem. Soc.* **144**, 14505-14516 (2022).
15. Guo, B. *et al.* Confined cobalt single-atom catalysts with strong electronic metal-support interactions based on a biomimetic self-assembly strategy. *Carbon Energy* **6**, e554 (2024).
16. Jiang, X. *et al.* Cu single-atom catalysts for high-selectivity electrocatalytic acetylene semihydrogenation. *Angew. Chem. Inter. Ed.* **62**, e202307848 (2023).
17. Zhou, S. *et al.* FeN_3_S_1_-OH single-atom sites anchored on hollow porous carbon for highly efficient pH-universal oxygen reduction reaction. *Small* **20**, 2310224 (2024).
18. Li, Y. *et al.* Ruthenium nanoclusters and single atoms on α-MoC/N-doped carbon achieves low-input/input-free hydrogen evolution via decoupled/coupled hydrazine oxidation. *Angew. Chem. Inter. Ed.* **63**, e202316755 (2024).
19. Zuo, S. *et al.* Local compressive strain-induced anti-corrosion over isolated Ru-decorated Co_3_O_4_ for efficient acidic oxygen evolution. *Nat. Commun.* **15**, 9514 (2024).
20. Yao, Z. *et al.* Hydrogen radical-boosted electrocatalytic CO_2_ reduction using Ni-partnered heteroatomic pairs. *Nat. Commun.* **15**, 9881 (2024).
21. Hou, M. *et al.* Microenvironment reconstitution of highly active Ni single atoms on oxygen-incorporated Mo_2_C for water splitting. *Nat. Commun.* **15**, 1342 (2024).
22. Zhang, L. *et al.* High-density asymmetric iron dual-atom sites for efficient and stable electrochemical water oxidation. *Nat. Commun.* **15**, 9440 (2024).
23. Fan, W. *et al.* Rational design of heterogenized molecular phthalocyanine hybrid single-atom electrocatalyst towards two-electron oxygen reduction. *Nat. Commun.* **14**, 1426 (2023).
24. Zhang, Z. *et al.* Single-atom platinum with asymmetric coordination environment on fully conjugated covalent organic framework for efficient electrocatalysis. *Nat. Commun.* **15**, 2556 (2024).
25. Yang, H. *et al.* Carbon dioxide electroreduction on single-atom nickel decorated carbon membranes with industry compatible current densities. *Nat. Commun.* **11**, 593 (2020).
26. Gu, Y. et al. Industrial electrosynthesis of hydrogen peroxide over p-block metal single sites. *Nat. Synth.* **4**, 614-621 (2025).
27. Cao, P. *et al.* Metal single-site catalyst design for electrocatalytic production of hydrogen peroxide at industrial-relevant currents. *Nat. Commun.* **14**, 172 (2023).
28. Lin, Z. *et al.* Atomic Co decorated free-standing graphene electrode assembly for efficient hydrogen peroxide production in acid. *Energy Environ. Sci.* **15**, 1172-1182 (2022).
29. Lee, B.-H. et al. Supramolecular tuning of supported metal phthalocyanine catalysts for hydrogen peroxide electrosynthesis. *Nat. Catal.* **6**, 234-243 (2023).
30. Tian, Z. et al. Constructing interfacial boron-nitrogen moieties in turbostratic carbon for electrochemical hydrogen peroxide production. *Angew. Chem. Int. Ed.* **61**, e20220691 (2022).
31. Du, J. et al. CoIn dual-atom catalyst for hydrogen peroxide production via oxygen reduction reaction in acid. *Nat. Commun.* **14**, 4766 (2023).
32. Cai, X.-H. et al. Kirkendall effect-driven interface engineering facilitates water dissociation for dual-site H_2_O_2_ electrosynthesis simultaneously at the anode and cathode. *Angew. Chem. Int. Ed.* e202512046 (2025).
33. Liu, C. *et al.* Heterogeneous molecular Co-N-C catalysts for efficient electrochemical H_2_O_2_ synthesis. *Energy Environ. Sci.* **16**, 446-459 (2023).
34. Xia, C., Xia, Y., Zhu, P., Fan, L. & Wang, H. Direct electrosynthesis of pure aqueous H_2_O_2_ solutions up to 20% by weight using a solid electrolyte. *Science* **366**, 226-231 (2019).
35. Xia, Y. et al. Highly active and selective oxygen reduction to H_2_O_2_ on boron-doped carbon for high production rates. *Nat. Commun.* **12**, 4225 (2021).
36. Long, Y. *et al.* Tailoring the atomic-local environment of carbon nanotube tips for selective H_2_O_2_ electrosynthesis at high current densities. *Adv. Mater.* **35**, 2303905 (2023).
37. Fan, L. et al. CO_2_/carbonate-mediated electrochemical water oxidation to hydrogen peroxide. *Nat. Commun.* **13**, 2668 (2022).
38. Li, H. *et al.* Scalable neutral H_2_O_2_ electrosynthesis by platinum diphosphide nanocrystals by regulating oxygen reduction reaction pathways. *Nat. Commun.* **11**, 3928 (2020).
39. Zhang, T., Wang, W., Liu, W., Guo, Z. & Liu, J. Residual ligand-functionalized ultrathin Ni(OH)_2_ via reconstruction for high-rate HO_2_^−^ electrosynthesis. *Nat. Commun.* **16**, 5240 (2025).
40. Huang, H. *et al.* Enhancing H_2_O_2_ electrosynthesis at industrial-relevant current in acidic media on diatomic cobalt sites. *J. Am. Chem. Soc.* **146**, 9434-9443 (2024).
41. Zhu, X.-D. *et al.* CoSe_2_ supported single Pt site catalysts for hydrogen peroxide generation via two-electron oxygen reduction. *SusMat* **3**, 334-344 (2023).
42. Zou, H. *et al.* Pushing the limit of atomically dispersed Au catalysts for electrochemical H_2_O_2_ production by precise electronic perturbation of the active site. *Chem Catal.* **3**, 100583 (2023).
43. Zhang, S. *et al.* Single-atom Co-O_4_ sites embedded in a defective-rich porous carbon layer for efficient H_2_O_2_ electrosynthesis. *Small* **20**, 2310468 (2024).
44. Chen, S. *et al.* Tuning proton affinity on Co-N-C atomic interface to disentangle activity-selectivity trade-off in acidic oxygen reduction to H_2_O_2_. *Angew. Chem. Inter. Ed.* e202418713 (2024).
45. Chen, Y. *et al.* Oxygen functional groups regulate cobalt-porphyrin molecular electrocatalyst for acidic H_2_O_2_ electrosynthesis at industrial-level current. *Angew. Chem. Inter. Ed.* **63**, e202407163 (2024).
46. Ni, B. *et al.* Second-shell N dopants regulate acidic O_2_ Reduction pathways on isolated Pt sites. *J. Am. Chem. Soc.* **146**, 11181-11192 (2024).
47. Zeng, Y., Tan, X., Zhuang, Z., Chen, C. & Peng, Q. Nature-inspired N, O Co-coordinated manganese single-atom catalyst for efficient hydrogen peroxide electrosynthesis. *Angew. Chem. Inter. Ed.* e202416715 (2024).
48. Du, Y.-X. *et al.* Carbon black-supported single-atom Co-N-C as an efficient oxygen reduction electrocatalyst for H_2_O_2_ production in acidic media and microbial fuel cell in neutral media. *Adv. Funct. Mater.* **33**, 2300895 (2023).
49. Sun, Y. *et al.* Activity-selectivity trends in the electrochemical production of hydrogen peroxide over single-site metal-nitrogen-carbon catalysts. *J. Am. Chem. Soc.* **141**, 12372-12381 (2019).
50. Zhang, E. *et al.* Engineering the local atomic environments of indium single-atom catalysts for efficient electrochemical production of hydrogen peroxide. *Angew. Chem. Inter. Ed.* **61**, e202117347 (2022).
51. Zhang, J. *et al.* Design of hierarchical, three-dimensional free-standing single-atom electrode for H_2_O_2_ production in acidic media. *Carbon Energy* **2**, 276-282 (2020).
52. Wang, Y. *et al.* High-efficiency oxygen reduction to hydrogen peroxide catalyzed by nickel single-atom catalysts with tetradentate N_2_O_2_ coordination in a three-phase flow cell. *Angew. Chem. Inter. Ed.* **59**, 13057-13062 (2020).
53. Jiang, K. *et al.* Highly selective oxygen reduction to hydrogen peroxide on transition metal single atom coordination. *Nat. Commun.* **10**, 3997 (2019).
54. Li, Y. *et al.* Single-atom iron catalyst with biomimetic active center to accelerate proton spillover for medical-level electrosynthesis of H_2_O_2_ disinfectant. *Angew. Chem. Inter. Ed.* **62**, e202306491 (2023).
55. Shen, H. *et al.* Selective and continuous electrosynthesis of hydrogen peroxide on nitrogen-doped carbon supported nickel. *Cell. Rep. Phys. Sci.* **1**, 11 (2020).
56. Wei, G. *et al.* Single-atom zinc sites with synergetic multiple coordination shells for electrochemical H_2_O_2_ production. *Angew. Chem. Inter. Ed.* **62**, e202313914 (2023).
57. Yan, M. *et al.* Sb_2_S_3_-templated synthesis of sulfur-doped Sb-N-C with hierarchical architecture and high metal loading for H_2_O_2_ electrosynthesis. *Nat. Commun.* **14**, 368 (2023).
58. Dong, L.-Y. *et al.* Boundary-rich carbon-based electrocatalysts with manganese(ii)-coordinated active environment for selective synthesis of hydrogen peroxide. *Angew. Chem. Inter. Ed.* **63**, e202317660 (2024).
59. Xu, H. *et al.* Atomically dispersed iron regulating electronic structure of iron atom clusters for electrocatalytic H_2_O_2_ production and biomass upgrading. *Angew. Chem. Inter. Ed.* **62**, e202314414 (2023).
60. Yang, H. *et al.* Ultra-low single-atom Pt on g-C_3_N_4_ for electrochemical hydrogen peroxide production. *Carbon Energy* **5**, e337 (2023).
61. Liu, J. *et al.* Single-atom CoN_4_ sites with elongated bonding induced by phosphorus doping for efficient H_2_O_2_ electrosynthesis. *Appl. Catal. B Environ.* **324**, 122267 (2023).
62. Gu, Y. *et al.* Industrial electrosynthesis of hydrogen peroxide over p-block metal single sites. *Nat. Synth.* 1-8 (2025).
63. Zhao, Q. *et al.* Approaching a high-rate and sustainable production of hydrogen peroxide: oxygen reduction on Co-N-C single-atom electrocatalysts in simulated seawater. *Energy Environ. Sci.* **14**, 5444-5456 (2021).
64. Iwasaki, T., Masuda, Y., Ogihara, H. & Yamanaka, I. Direct synthesis of pure H_2_O_2_ aqueous solution by CoTPP/ketjen-black electrocatalyst and the fuel cell reactor. *Electrocatalysis* **9**, 236-242 (2018).
65. Yamanaka, I. *et al.* Study of direct synthesis of hydrogen peroxide acid solutions at a heat-treated MnCl-porphyrin/activated carbon cathode from H_2_ and O_2_. *J. Phys. Chem. C* **116**, 4572-4583 (2012).
66. Xiao, C. *et al.* Super-coordinated nickel N_4_Ni_1_O_2_ site single-atom catalyst for selective H_2_O_2_ electrosynthesis at high current densities. *Angew. Chem. Inter. Ed.* **61**, e202206544 (2022).
67. Kim, C. *et al.* Concurrent oxygen reduction and water oxidation at high ionic strength for scalable electrosynthesis of hydrogen peroxide. *Nat. Commun.* **14**, 5822 (2023).
68. Zhang, C. *et al.* Crystal engineering enables cobalt-based metal–organic frameworks as high-performance electrocatalysts for H_2_O_2_ production. *J. Am. Chem. Soc.* **145**, 7791-7799 (2023).
69. Chang, Q. *et al.* Promoting H_2_O_2_ production via 2-electron oxygen reduction by coordinating partially oxidized Pd with defect carbon. *Nat. Commun.* **11**, 2178 (2020).
70. Mei, X. *et al.* Highly efficient H_2_O_2_ production via two-electron electrochemical oxygen reduction over Fe-doped CeO_2_. *ACS Sustain. Chem Eng.* **11**, 15609-15619 (2023).
71. Jing, L. *et al.* Efficient neutral H_2_O_2_ electrosynthesis from favorable reaction microenvironments via porous carbon carrier engineering. *Angew. Chem. Inter. Ed.* **63**, e202403023 (2024).
72. Jin, M. *et al.* Highly dispersed Ag clusters for active and stable hydrogen peroxide production. *Nano Res.* **15**, 5842-5847 (2022).
73. Chen, Z. *et al.* Amorphous low-coordinated cobalt sulphide nanosheet electrode for electrochemically synthesizing hydrogen peroxide in acid media. *Appl. Cataly. B Environ.* **334**, 122825 (2023).
74. Li, Y. *et al.* Accelerated proton-coupled electron transfer via engineering palladium sub-nanoclusters for scalable electrosynthesis of hydrogen peroxide. *Angew. Chem. Inter. Ed.* **64**, e202413159 (2025).
75. Pei, Z. *et al.* Low-coordinated conductive ZnCu Metal-organic frameworks for highly selective H_2_O_2_ electrosynthesis. *Small* **20**, 2403808 (2024).
76. Bao, Z. *et al.* Tuning the ratio of Bi/Bi_2_O_3_ in Bi/PNC nanosheet for high-efficiency electrosynthesis hydrogen peroxide. *Nano Res.* **16**, 9050-9058 (2023).
77. Yu, Z. *et al.* Low-coordinated Pd site within amorphous palladium selenide for active, selective, and stable H_2_O_2_ electrosynthesis. *Adv. Mater.* **35**, 2208101 (2023).
78. Wu, J. *et al.* Highly selective O_2_ reduction to H_2_O_2_ catalyzed by cobalt nanoparticles supported on nitrogen-doped carbon in alkaline solution. *ACS Catal.* **11**, 5035-5046 (2021).
79. Xia, B. *et al.* Dynamic gas-diffusion electrodes for oxygen electroreduction to hydrogen peroxide. *AIChE J.* **69**, e18022 (2023).
80. Liu, M. *et al.* Construction of atomic metal-N_2_ sites by interlayers of covalent organic frameworks for electrochemical H_2_O_2_ synthesis. *Small* **18**, 2204757 (2022).
81. Liu, M. *et al.* Self-nanocavity-confined halogen anions boosting the high selectivity of the two-electron oxygen reduction pathway over Ni-based MOFs. *J. Phy. Chem. Lett.* **12**, 8706-8712 (2021).
82. Shen, R. *et al.* High-concentration single atomic Pt sites on hollow CuS_x_ for selective O_2_ reduction to H_2_O_2_ in acid solution. *Chem* **5**, 2099-2110 (2019).
83. Zhang, J. *et al.* Strong metal-support interaction boosts activity, selectivity, and stability in electrosynthesis of H_2_O_2_. *J. Am. Chem. Soc.* **144**, 2255-2263 (2022).
84. Li, W., Bonakdarpour, A., Gyenge, E. & Wilkinson, D. P. Design of bifunctional electrodes for co-generation of electrical power and hydrogen peroxide. *J. Appl. Electrochem.* **48**, 985-993 (2018).
85. Yamanaka, I., Tazawa, S., Murayama, T., Iwasaki, T. & Takenaka, S. Catalytic synthesis of neutral hydrogen peroxide at a CoN_2_C cathode of a polymer electrolyte membrane fuel cell (PEMFC). *ChemSusChem* **3**, 59-62 (2010).
86. Zhang, Y. *et al.* Metastable hexagonal phase SnO_2_ nanoribbons with active edge sites for efficient hydrogen peroxide electrosynthesis in neutral media. *Angew. Chem. Inter. Ed.* **62**, e202218924 (2023).
87. Zhang, Q. *et al.* Bifunctional oxygen-defect bismuth catalyst toward concerted production of H_2_O_2_ with over 150% cell faradaic efficiency in continuously flowing paired-electrosynthesis system. *Adv. Mater.* **36**, 2408341 (2024).
88. Zhou, Y. *et al.* The operation active sites of O_2_ reduction to H_2_O_2_ over ZnO. *Energy Environ. Sci.* **16**, 3526-3533 (2023).
89. Yan, L. *et al.* Exsolved Co_3_O_4_ with tunable oxygen vacancies for electrocatalytic H_2_O_2_ production. *Mater. Today Energy* **24**, 100931 (2022).
90. Sun, X. *et al.* Active-site-transformation-promoted electrochemical H_2_O_2_ production on carbon-wrapped copper oxides. *CCS Chem.* **0**, 1-12 (2024).
91. Wu, J. *et al.* Composition engineering of amorphous nickel boride nanoarchitectures enabling highly efficient electrosynthesis of hydrogen peroxide. *Adv. Mater.* **34**, 2202995 (2022).
92. Chen, G. *et al.* Isolated active sites in perovskite lattice for efficient production of hydrogen peroxide. *Matter* **7**, 2265-2277 (2024).
93. Gao, R. *et al.* Engineering facets and oxygen vacancies over hematite single crystal for intensified electrocatalytic H_2_O_2_ production. *Adv. Funct. Mater.* **30**, 1910539 (2020).
94. Shi, Q. *et al.* Highly dispersed platinum atoms on the surface of AuCu metallic aerogels for enabling H_2_O_2_ production. *ACS Appl. Energy Mater.* **2**, 7722-7727 (2019).
95. Sheng, H. *et al.* Stable and selective electrosynthesis of hydrogen peroxide and the electro-Fenton process on CoSe_2_ polymorph catalysts. *Energy Environ. Sci.* **13**, 4189-4203 (2020).
96. Xia, F. *et al.* Carbon free and noble metal free Ni_2_Mo_6_S_8_ electrocatalyst for selective electrosynthesis of H_2_O_2_. *Adv. Funct. Mater.* **31**, 2104716 (2021).
97. Wu, Z., Wang, T., Zou, J.-J., Li, Y. & Zhang, C. Amorphous nickel oxides supported on carbon nanosheets as high-performance catalysts for electrochemical synthesis of hydrogen peroxide. *ACS Catal.* **12**, 5911-5920 (2022).
98. Ross, R. D., Sheng, H., Parihar, A., Huang, J. & Jin, S. Compositionally tuned trimetallic thiospinel catalysts for enhanced electrosynthesis of hydrogen peroxide and built-in hydroxyl radical generation. *ACS Catal.* **11**, 12643-12650 (2021).
99. Zheng, Y.-R. *et al.* Black phosphorous mediates surface charge redistribution of CoSe_2_ for electrochemical H_2_O_2_ production in acidic electrolytes. *Adv. Mater.* **34**, 2205414 (2022).
100. Zhi, Q. *et al.* Dithiine-linked metalphthalocyanine framework with undulated layers for highly efficient and stable H_2_O_2_ electroproduction. *Nat. Commun.* **15**, 678 (2024).
101. Sun, Y. *et al.* Efficient electrochemical hydrogen peroxide production from molecular oxygen on nitrogen-doped mesoporous carbon catalysts. *ACS Catal.* **8**, 2844-2856 (2018).
102. Liu, Y., Quan, X., Fan, X., Wang, H. & Chen, S. High-yield electrosynthesis of hydrogen peroxide from oxygen reduction by hierarchically porous carbon. *Angew. Chem. Inter. Ed.* **54**, 6837-6841 (2015).
103. Fellinger, T.-P., Hasché, F., Strasser, P. & Antonietti, M. Mesoporous nitrogen-doped carbon for the electrocatalytic synthesis of hydrogen peroxide. *J. Am. Chem. Soc.* **134**, 4072-4075 (2012).
104. Han, L. *et al.* In-plane carbon lattice-defect regulating electrochemical oxygen reduction to hydrogen peroxide production over nitrogen-doped graphene. *ACS Catal.* **9**, 1283-1288 (2019).
105. Lee, K. *et al.* Structure-controlled graphene electrocatalysts for high-performance H_2_O_2_ production. *Energy Environ. Sci.* **15**, 2858-2866 (2022).
106. Lu, Z. *et al.* High-efficiency oxygen reduction to hydrogen peroxide catalysed by oxidized carbon materials. *Nat. Catal.* **1**, 156-162 (2018).
107. Li, L. *et al.* Tailoring selectivity of electrochemical hydrogen peroxide generation by tunable pyrrolic-nitrogen-carbon. *Adv. Energy Mater.* **10**, 2000789 (2020).
108. Huang, S. *et al.* Covalent organic frameworks with molecular electronic modulation as metal-free electrocatalysts for efficient hydrogen peroxide production. *Small Struct.* **4**, 2200387 (2023).
109. Zhang, Y. *et al.* Multicomponent synthesis of imidazole-linked fully conjugated 3D covalent organic framework for efficient electrochemical hydrogen peroxide production. *Angew. Chem. Inter. Ed.* **62**, e202314539 (2023).
110. Zhang, H.-X. *et al.* Electrocatalyst derived from fungal hyphae and its excellent activity for electrochemical production of hydrogen peroxide. *Electrochim. Acta* **308**, 74-82 (2019).
111. Zhao, K. *et al.* Enhanced H_2_O_2_ production by selective electrochemical reduction of O_2_ on fluorine-doped hierarchically porous carbon. *J. Catal.* **357**, 118-126 (2018).
112. Zhang, Q. *et al.* Highly efficient electrosynthesis of hydrogen peroxide on a superhydrophobic three-phase interface by natural air diffusion. *Nat. Commun.* **11**, 1731 (2020).
113. Yamanaka, I. & Murayama, T. Neutral H_2_O_2_ synthesis by electrolysis of water and O_2_. *Angew. Chem. Inter. Ed.* **47**, 1900-1902 (2008).
114. Yamanaka, I. Direct synthesis of H_2_O_2_ by a H_2_/O_2_ fuel cell. *Catal. Surv. from Asia* **12**, 78-87 (2008).
115. Cao, P. *et al.* Durable and selective electrochemical H_2_O_2_ synthesis under a large current enabled by the cathode with highly hydrophobic three-phase architecture. *ACS Catal.* **11**, 13797-13808 (2021).
